# ANGPTL4 negatively regulates the progression of osteosarcoma by remodeling branched-chain amino acid metabolism

**DOI:** 10.1038/s41420-022-01029-x

**Published:** 2022-04-23

**Authors:** Shanyi Lin, Yu Miao, Xu Zheng, Yang Dong, Qingcheng Yang, Quanjun Yang, Silin Du, Jun Xu, Shumin Zhou, Ting Yuan

**Affiliations:** 1 Department of Orthopaedic Surgery, Shanghai Jiao Tong University Affiliated Sixth People’s Hospital, Shanghai, China; 2 Institute of Microsurgery on Extremities, Shanghai Jiao Tong University Affiliated Sixth People’s Hospital, Shanghai, China; 3 Department of Pharmacy, Shanghai Jiao Tong University Affiliated Sixth People’s Hospital, Shanghai, China; 4 University Hospital, Shanghai Jiao Tong University, Shanghai, China

**Keywords:** Bone cancer, TOR signalling

## Abstract

Angiopoietin-like-4 (ANGPTL4), a secreted glycoprotein that is mainly known as a regulator in lipid metabolism, now, is also indicated to be involved in the regulation of cancer progression and metastasis. However, little is known about not only biological functions, but also underlying mechanism of ANGPTL4 in the progression of osteosarcoma (OS). Here, we discovered that ANGPTL4 is downregulated in OS, and is associated with branched-chain amino acid (BCAA) metabolism. The BCAAs (valine, leucine, and isoleucine) are essential amino acids that play an important role in metabolic regulation. Aberrant BCAA metabolism is also found in various cancers and is associated with tumor progression, including proliferation, invasion, and metastasis. In this study, we indicated that the negative relation between the expression of ANGPTL4 and BCAA catabolism in OS samples and cell lines. The knockdown of *ANGPTL4* in OS cells resulted in the accumulation of BCAAs, which in turn activated the mTOR signaling pathway, enhancing OS cell proliferation. Thus, reduced expression of ANGPTL4 is associated with the progression of OS. Taken together, our results demonstrated that the ANGPTL4/BCAA/mTOR axis is an important pathway in OS progression and may be a potential therapeutic target to slow OS progression.

## Introduction

Osteosarcoma (OS) is a major health burden and cause of cancer-related death in adolescents worldwide [1]. Patients diagnosed with OS have a risk of amputation and even death after tumor cells metastasize to the lungs [2]. Currently, OS treatment includes complete tumor removal and two rounds of chemotherapy (i.e., preoperative and postoperative chemotherapy), which continue for at least 6 months [2]. However, these systematic therapies only cure 60–70% of patients. The 5-year survival rate of OS patients has not increased, although surgical techniques have improved substantially in recent decades [2, 3]. To improve the prognosis of OS patients, researchers are conducting clinical trials of second-line or third-line drugs [4, 5]. Elucidation of the mechanisms underlying the occurrence and progression of OS is important for the development of more drugs against OS.

Angiopoietin-like proteins (ANGPTLs) are a protein family with eight members that are structurally similar to angiogenin [6]. ANGPTL4, a member of the ANGPTL family that is mainly found in the liver, adipose tissue, and skeletal muscle, is well known as an inhibitor of lipoprotein lipase [7, 8]. Due to the organ-specific expression of ANGPTL4, it was initially regarded as a metabolic regulator that maintains metabolic homeostasis [8, 9]. Recently, ANGPTL4 was detected in diverse tumor cells and associated with malignant phenotypes. Chen et al. found that the expression of ANGPTL4 was higher in gastric cancer cells and that the knockdown of ANGPTL4 could suppress the development of gastric cancer [10]. However, Cai et al. demonstrated that ANGPTL4 was a favorable prognostic factor in breast cancer and that upregulation of ANGPTL4 expression induced the suppression of adhesion and migration in cancer cells [11]. In addition, ANGPTL4 was shown to be involved in temozolomide resistance in glioblastoma by promoting cancer stemness [12]. Although researchers have started to explore the role of ANGPTL4 in OS [13], the underlying fine molecular mechanism remains unclear.

In the present study, decreased expression of ANGPTL4 was found in both clinical OS tissue samples and OS cell lines compared to the normal controls. Knockdown of *ANGPTL4* accelerated proliferation in OS cells. RNA sequencing (RNA-seq) analysis showed that *ANGPTL4* expression resulted in the remodeling of branched-chain amino acid (BCAA) metabolism. BCAAs are essential amino acids (leucine, isoleucine and valine) that play a crucial role in protein synthesis and energy supply and are indispensable for cell growth [14, 15]. When the accumulation of BCAAs was increased in OS cell lines due to knockdown of *ANGPTL4*, the mTOR signaling pathway was activated, which resulted in enhanced growth of OS cells. Taken together, our results indicated that low expression of ANGPLT4 promoted the progression of OS via mTOR signaling by remodeling BCAA metabolism.

## Results

### ANGPTL4 was expressed at low levels in clinical OS samples and OS cells

**Fig. 1:**
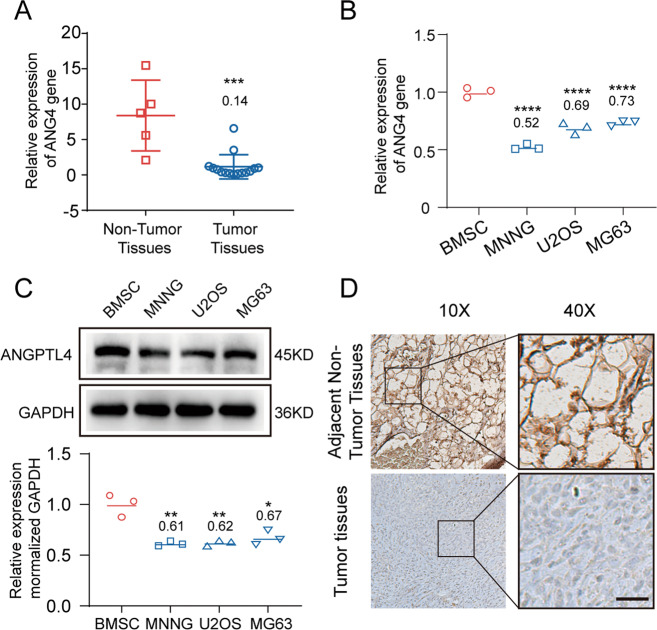
ANGPTL4 expression is lower in osteosarcoma tissue and osteosarcoma cell lines. **A** Relative mRNA expression of *ANGPTL4* in clinical osteosarcoma (OS) tissue samples (*N* = 15) and adjacent nontumor cancellous bone tissue samples (*N* = 5) (***p < 0.001 vs. the normal tissue group). **B** Relative mRNA expression of *ANGPTL4* in OS cell lines (MNNG, U2OS, MG63) and bone marrow-derived stromal cells (BMSCs). (*N* = 3, ****p < 0.0001 vs. the BMSC group). **C** ANGPTL4 expression (upper panels) and quantitation of protein levels (lower bar graphs) in OS cell lines and BMSCs were detected by western blot (*N* = 3, *p < 0.05, **p < 0.01 vs. the BMSC group). **D** Representative immunohistochemical images of ANGPTL4 in clinical OS sample and adjacent nontumor bone marrow sample (**D**, scale bars, 100 μm).

To investigate the role of ANGPTL4 in the development of OS, we determined the mRNA expression levels of *ANGPTL4* in clinical OS tissue samples and control samples (normal cancellous bone). As shown in Fig. 1A, *ANGPTL4* expression was lower in the OS tissues than in the normal bone tissues (the ratio of OS tissues vs. nontumor tissues was 0.14 ± 0.19). Then, we further analyzed the expression of ANGPTL4 protein by IHC staining of both the OS tissue sections and the adjacent nontumor sections. Similar to the RT-qPCR results, the OS tissues also showed a smaller ANGPTL4-positive area than the control tissues (Fig. 1D). In addition, the expression of *ANGPTL4* was verified in three OS cell lines, MNNG, U2OS, and MG63, and one control cell line, BMSC, at both the mRNA and protein levels. The mRNA and protein expression levels of *ANGPTL4* in the MNNG, U2OS, and MG63 cells were 0.52 ± 0.02-, 0.69 ± 0.04-, and 0.73 ± 0.02- and 0.61 ± 0.02-, 0.62 ± 0.04-, and 0.67 ± 0.06-fold lower than those in BMSCs, respectively (Fig. 1B, C). These data confirmed that the OS tissues and cell lines had lower expression of ANGPTL4 than the cancellous bones and BMSCs at both the mRNA and protein levels.

### Downregulation of ANGPTL4 expression promoted the growth of OS cells in vitro

**Fig. 2:**
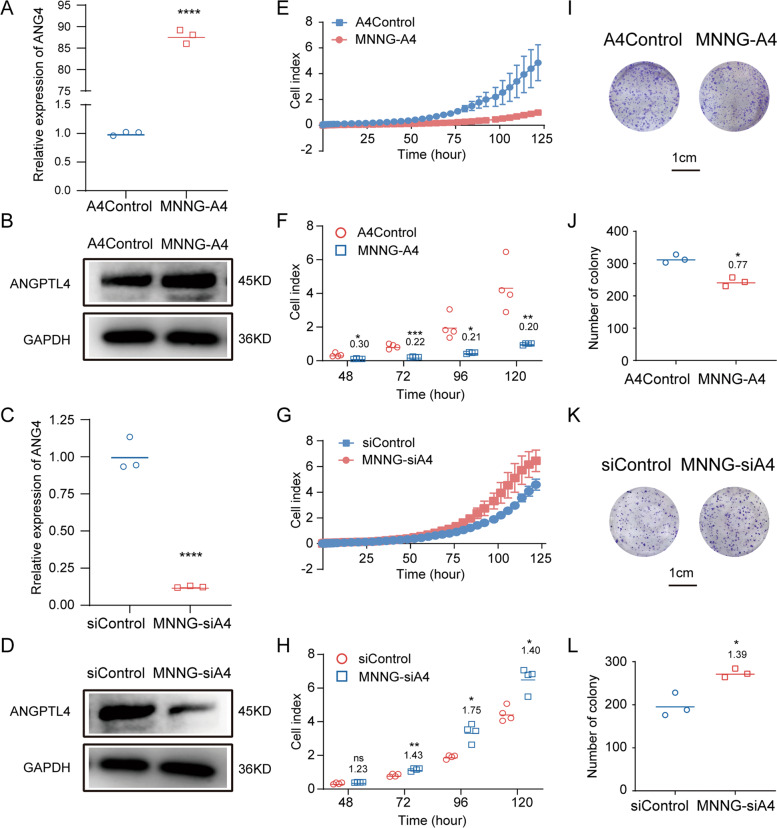
Knockdown of *ANGPTL4* promotes the growth of MNNG cells in vitro. **A**–**D** Quantitation of *ANGPTL4* mRNA and ANGPTL4 protein expression in MNNG-A4, MNNG-siA4, and control cell lines by RT-qPCR and western blot, respectively (*N* = 3, ****p < 0.0001 vs. the control group). **E**, **G** In vitro measurement of cell proliferation in two ANGPTL4-regulated MNNG cell lines (MNNG-A4, MNNG-siA4) and control cell lines. The cell index results indicate that cell proliferation is enhanced in the MNNG-siA4 cells but reduced in the MNNG-A4 cells compared to the control cells (*N* = 4). **F**, **H** Cell index at 48, 72, 96, and 120 h (*N* = 4, ns *p* > 0.05, *p < 0.05, **p < 0.01, ***p < 0.001 vs. the control group). **I**, **K** Representative images of colony formation assays of the MNNG-A4, MNNG-siA4, and control cells. **J**, **L** Quantitation of colony counts after 2 weeks of in vitro maintenance (*N* = 3, *p < 0.05 vs. the control group).

Our previous results suggested that ANGPTL4 may have a negative correlation with the progression of OS. Therefore, how ANGPTL4 affects OS growth was investigated. Among the three OS cell lines we tested, MNNG cells showed the lowest ANGPTL4 expression compared to BMSCs. Thus, the MNNG cell line was selected as an in vitro model for validation. Using a lentivirus system, we constructed stable OS cell lines with upregulated (MNNG-A4) and downregulated (MNNG-siA4) expression of *ANGPTL4*, as well as their control cell lines (A4Control and siControl) to further determine the biological role of ANGPTL4 in MNNG cell growth. As shown in Fig. 2A–D, the successful construction of MNNG-A4 (*ANGPTL4-*overexpressing stable OS cell lines) and MNNG-siA4 (*ANGPTL4-*lowexpressing stable OS cell lines) was confirmed at both the mRNA and protein levels by RT-qPCR and western blot.

We then evaluated the cell proliferation of these two *ANGPTL4*-regulated MNNG cell lines with their control cells by RTCA assays [16]. The results showed that the cell growth was significantly suppressed in the MNNG cells overexpressing *ANGPTL4* (the ratio of MNNG-A4 vs. A4Control was 0.30 ± 0.01, 0.22 ± 0.01, 0.21 ± 0.02, and 0.20 ± 0.01 at 48, 72, 96, and 120 h, respectively). In contrast the knockdown of *ANGPTL4* in MNNG cells enhanced their growth (the ratio of MNNG-siA4 vs. siControl was 1.23 ± 0.04, 1.43 ± 0.09, 1.75 ± 0.30, and 1.40 ± 0.15 at 48, 72, 96, and 120 h, respectively) (Fig. 2E–H). Accordingly, the colony formation assays were also consistent with the cell proliferation assays (the ratio of MNNG-A4 vs. A4Control and MNNG-siA4 vs. siControl was 0.77 ± 0.04 and 1.39 ± 0.04, respectively) (Fig. 2I–L). Taken together, these results demonstrated that the expression of ANGPTL4 was negatively correlated with the growth of OS cells in vitro.

### ANGPTL4 activated the mTOR signal pathway by remodeling BCAA metabolism

**Fig. 3:**
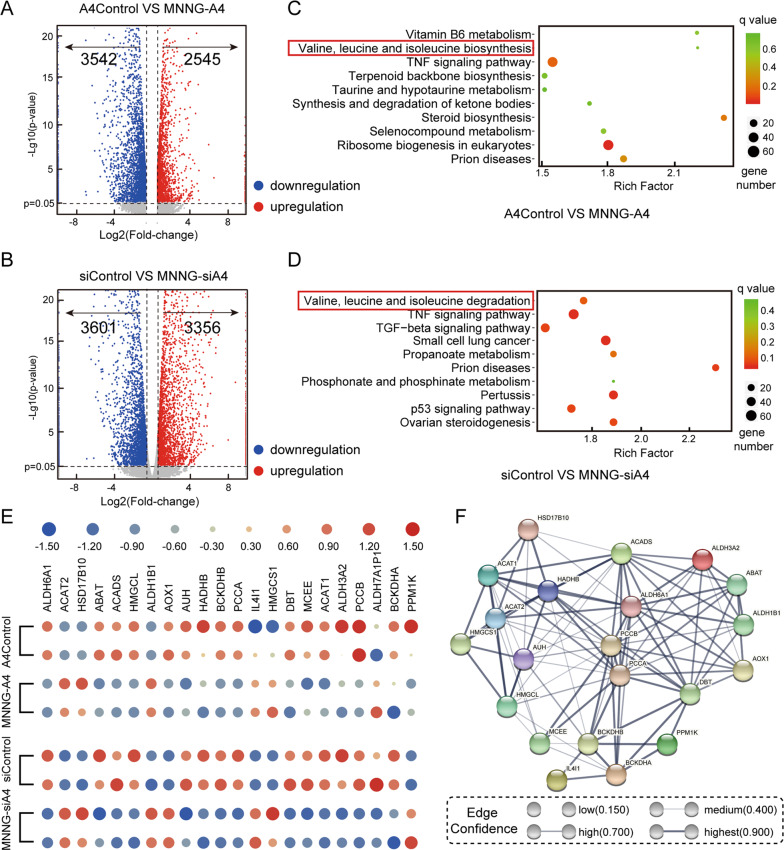
The signaling mechanism of the effects of ANGPTL4 on OS growth by RNA-seq. **A**, **B** Volcano plots of the differentially expressed genes (DEGs) in the MNNG-A4 cells, MNNG-siA4 cells, and control cells. Fold change values on the *X*-axis were log2-transformed while *p* values on the *Y*-axis were −log10 transformed. The screening criteria for DEGs were *p* value <0.05 and expression level higher than 1.5-fold or lower than 0.67-fold. **C**, **D** KEGG pathway enrichment analysis for DEGs in the MNNG-A4 cells, MNNG-siA4 cells, and control cells. **E** Heatmap of the BCAA-associated DEGs between the ANGPTL4-regulated cells and the control cells. **F** The protein-protein interaction network provided interactive information for the branched-chain amino acid (BCAA)-associated DEGs.

To explore the mechanism underlying the effects of ANGPTL4 on cell growth of MNNG cells, we employed mRNA sequencing to determine the altered signaling cascades by identifying DEGs between the *ANGPTL4*-regulated MNNG cells and their control cells. The DEG numbers are shown in Fig. 3A,B based on a cutoff value of a change in expression higher than 1.5- or lower than 0.67-fold. There were 6087 DEGs (2545 upregulated, 3542 downregulated) between the control and the ANGPTL4-overexpressing cell line, whereas in the ANGPTL4-lowexpressing group, the number was 6957 (3356 upregulated, 3601 downregulated).

Then, Kyoto Encyclopedia of Genes and Genomes analysis was performed on these DEGs to more detail the underlying mechanisms. As presented in Figs. 3C, D and S1, the results suggested that the DEGs in both *ANGPTL4*-regulated MNNG (overexpression and knockdown) cells were highly related to the metabolism of BCAAs. There were 22 of 46 genes that were differentially expressed in this gene cluster, and these genes are shown in Fig. 3E. To further elucidate the molecular mechanism of these genes, we then introduced these genes into the STRING (https://string-db.org/) database to construct the protein-protein interaction network. As shown in Fig. 3F, these genes are closely clustered together and form a complex network, which shows the precise inter-regulatory relationship of these genes that are highly involved in BCAA metabolism.

**Fig. 4:**
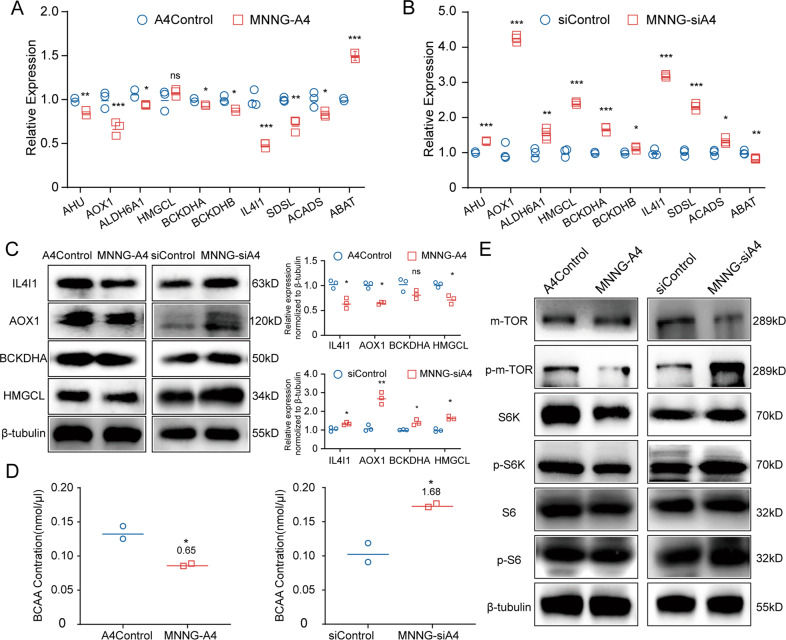
Low expression of ANGPTL4 promotes the accumulation of BCAAs and activates mTOR signaling. **A**, **B** Among the DEGs, genes that are highly related to BCAA metabolism were verified by RT-qPCR in the ANGPTL4-regulated cells and their controls (*N* = 3, ns *p* > 0.05, *p < 0.05, **p < 0.01, ***p < 0.001 vs. the control group). **C** Proteins that are highly related to the BCAA metabolism were verified by western blot in the ANGPTL4-regulated cells and their controls. Quantitation of relative expression (*N* = 3, ns *p* > 0.05, *p < 0.05, **p < 0.01 vs. the control group). **D** The relative levels of BCAAs in MNNG-A4 cells, MNNG-siA4 cells, and their control cells (*N* = 2, *p < 0.05, vs. the control group). **E** Western blot detection of proteins in the mTOR signaling pathway showed that this pathway was activated in the MNNG-siA4 cells but inhibited in the MNNG-A4 cells.

To verify the results of RNA-seq, we then detected the expressions of these genes in the *ANGPTL4*-regulated MNNG cells. The RT-qPCR results were consistent with the RNA-seq results. As shown in Fig. 4A, B, the expression of *HMGCL* and *ABAT* was upregulated while the expression of *AHU*, *AOX-1*, *ALDH6A1*, *BCKDHA*, *BCKDHB, IL4I-1, SDSL*, and *ACADS* was downregulated in the MNNG-A4 cells. These genes showed the opposite pattern in the MNNG-siA4 cells. Furthermore, the western blot results confirmed that the protein expression levels of some important genes (AOX-1, BCKDHA, IL4I-1, and HMGCL) were consistent with their mRNA levels (Fig. 4C). To further confirm our hypothesis, we measured the BCAA concentrations in both the MNNG-A4 and MNNG-siA4 cells and their control cells. As shown in Fig. 4D, the MNNG-A4 cells displayed a marked decrease in BCAAs compared with the control cells (MNNG-A4 vs. A4Control was 0.65 ± 0.01), and the BCAA content was significantly increased in the MNNG-siA4 cells (MNNG-siA4 vs. siControl was 1.68 ± 0.02).

According to previous studies, BCAAs can be catabolized into BCKAs and enter the TCA cycle [14, 15] and can also activate the mTOR signaling pathway and promote cell growth [17]. Thus, we then examined the activation of mTOR signaling in the *ANGPTL-*regulated MNNG cells and the control cells. As shown in Fig. 4E, the phosphorylation levels of mTOR together with the downstream effectors S6 kinase and S6 were upregulated in the MNNG-siA4 cells, while opposite expression patterns were found in the MNNG-A4 cells compared with the control cells. Overall, these data demonstrated that low expression of ANGPTL4 in MNNG cells increased the concentration of BCAAs, which in turn activated the mTOR pathway and promoted the progression of MNNG cells.

### The inhibition of BCATs attenuated the elevated proliferation in the *ANGPTL4*-silenced MNNG cells

**Fig. 5:**
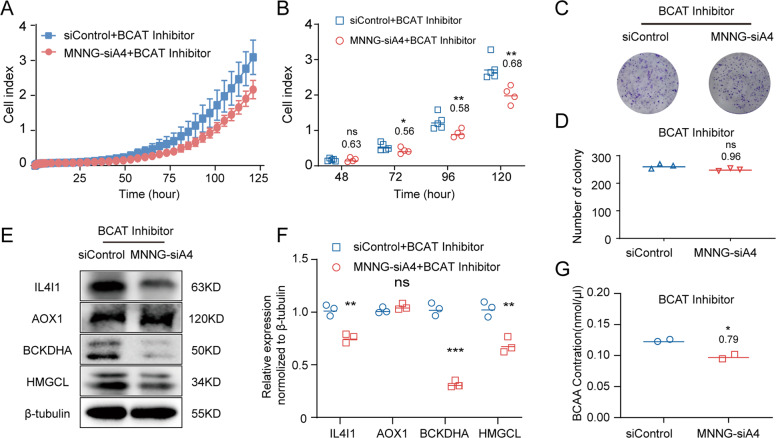
A BCAT inhibitor attenuates the elevated growth of MNNG-siA4 cells. **A** In vitro measurement of cell attachment and cell proliferation in the MNNG-siA4 and control cell lines with 5 μM BCAT inhibitor treatment. **B** Cell index at 48, 72, 96, and 120 h with 5 μM BCAT inhibitor treatment. Mean ± SD (*N* = 4, ns *p* > 0.05, *p < 0.05, **p < 0.01 vs. the control group). **C** Representative images of colony formation assays of the MNNG-siA4 and control cells after 5 μM BCAT inhibitor treatment. **D** Quantitation of colony counts with 5 μM BCAT inhibitor treatment. Mean ± SD (*N* = 3, ns *p* > 0.05 vs. the control group). **E**, **F** Proteins that are highly related to the BCAA metabolism were verified by western blot in the MNNG-siA4 cells and the control cells after 5 μM BCAT inhibitor treatment (*N* = 3, ns *p* > 0.05, **p < 0.01, ***p < 0.001 vs. the control group). **G** The relative levels of BCAAs in the MNNG-siA4 cells and the control cells after 5 μM BCAT inhibitor treatment (*N* = 2, *p < 0.05 vs. the control group).

As described above, when BCAAs are imported into the cells, they are first converted to branched-chain α-keto acids (BCKAs) by branched-chain amino acid transaminases (BCATs). This catalytic reaction is reversible and also produces BCAAs via BCKAs [15]. Although the RNA-seq results did not indicate that the expression levels of BCATs were directly regulated by ANGPTL4, the previous data we obtained suggested that ANGPTL4 affected BCAA metabolism by enhancing the activities of BCATs. To verify our hypothesis, we treated the MNNG-siA4 and siControl cells with a BCAT inhibitor, BCATc inhibitor 2 [18]. As expected, 5 μM of BCATc inhibitor 2 reduced the cell proliferation (the ratio of MNNG-siA4 + inhibitor vs. siControl + inhibitor was 0.63 ± 0.24, 0.56 ± 0.13, 0.58 ± 0.08, and 0.68 ± 0.07 at 48, 72, 96, and 120 h, respectively) and colony formation (the ratio of MNNG-siA4 + inhibitor vs. siControl + inhibitor was 0.96 ± 0.02) of the MNNG-siA4 cells (Fig. 5A–D). Mechanistically, the expression levels of BCAA metabolic signaling pathway-related proteins (AOX-1, BCKDHA, IL4I-1, and HMGCL) were also reversed in the MNNG-siA4 and siControl cells after 5 μM BCAT inhibitor treatment (Fig. 5E, F). Moreover, the BCAA concentrations were measured in the MNNG-siA4 and siControl cells after BCAT inhibitor treatment. The results showed that the high level of BCAAs induced by knockdown of *ANGPTL4* in OS cells was reduced by BCATc inhibitor 2 (MNNG-siA4 + inhibitor vs. siControl + inhibitor was 0.79 ± 0.03) (Fig. 5G). In summary, these data confirmed that downregulation of ANGPTL4 expression promotes MNNG growth by regulating the metabolism of BCAAs.

**Fig. 6:**
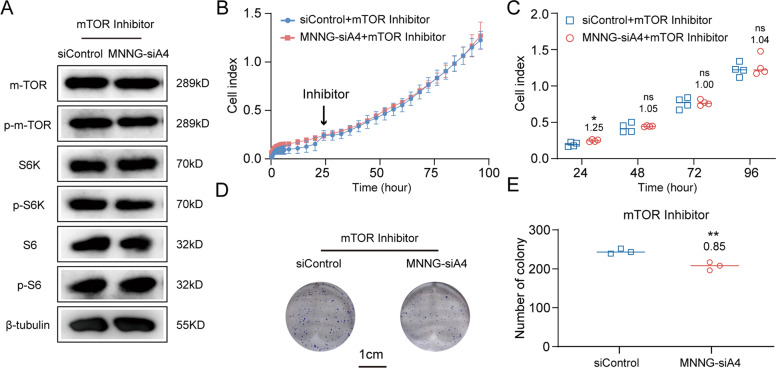
A mTOR inhibitor abolishes the elevated growth of MNNG-siA4 cells. **A** Western blot detection of proteins in the mTOR signaling pathway showed that activation of this pathway in MNNG-siA4 cells was reduced by mTOR inhibitor. **B** In vitro measurement of cell attachment and cell proliferation in the MNNG-siA4 and control cell lines with 0.2 μM mTOR inhibitor treatment. **C** Cell index at 24, 48, 72, and 96 h with 0.2 μM mTOR inhibitor treatment. Mean ± SD (*N* = 4, ns *p* > 0.05, *p < 0.05, vs. the control group). **D** Representative images of colony formation assays of the MNNG-siA4 and control cells after 0.2 μM mTOR inhibitor treatment. **E** Quantitation of colony counts with 0.2 μM mTOR inhibitor treatment. Mean ± SD (*N* = 3, **p < 0.01 vs. the control group).

To further confirm our hypothesis, we treated MNNG-siA4 and siControl cells with mTOR inhibitor. The activated mTOR signaling due to under-regulated ANGPTL4 was rescued by 0.2 μM mTOR inhibitor. As shown in Fig. 6A, the activation levels of mTOR, S6 kinase and S6 in MNNG-siA4 cells were as similar as those in siControl under mTOR inhibitor treatment. The results of RTCA assays also indicated that the high proliferation ability of MNNG-siA4 (before treated with mTOR inhibitor) could be attenuated in the same condition (inhibitor is added at 24 h). The ratios of the cell index of MNNG-siA4 + mTOR inhibitor vs. siControl + mTOR inhibitor was 1.25 ± 0.06, 1.05 ± 0.02, 1.00 ± 0.04, and 1.04 ± 0.10 at 24, 48, 72, and 96 h, respectively (Fig. 6B, C). Moreover, the clone formation test gives the further evidence (Fig. 6D, E). The ratio of clone formation abilities of MNNG-siA4 vs. siControl under mTOR inhibitor treatment was even reversed when compared to that before the treatment (the ratio of MNNG-siA4 + inhibitor vs. siControl + inhibitor was 0.85 ± 0.04, whereas the ratio of MNNG-siA4 vs. siControl was 1.39 ± 0.04) (Fig. 2L).

### ANGPTL4 attenuated OS progression via the BCAA/mTOR axis in vivo

**Fig. 7:**
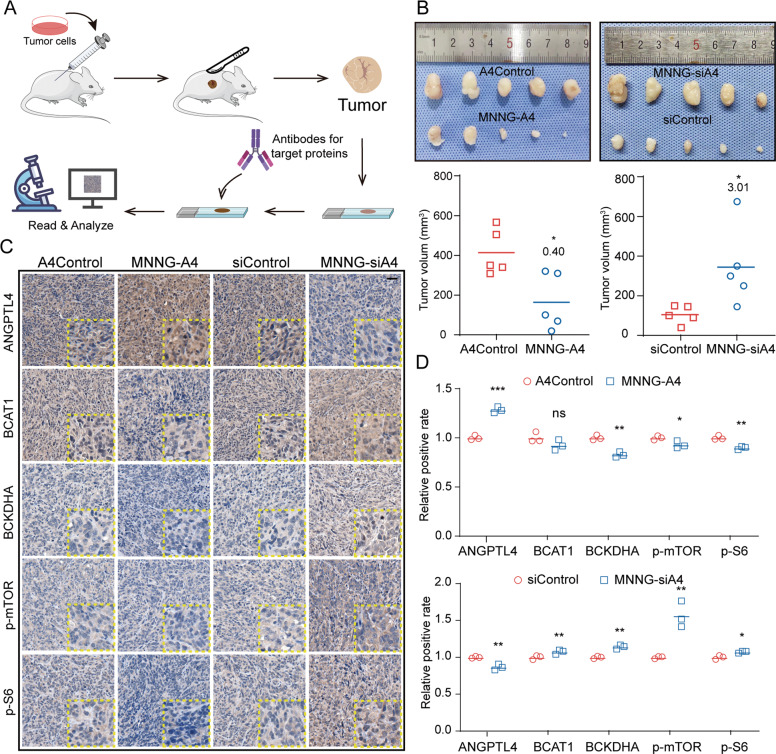
ANGPTL4 negatively regulates OS progression via the BCAA/mTOR axis in vivo. **A** Diagram illustrating the method for constructing the subcutaneous tumor model and IHC analysis. **B** Images of excised tumors from the nude mouse subcutaneous tumor model and quantitation of tumor volume (*N* = 5, *p < 0.05 vs. the control group). **C**, **D** Immunohistochemical staining of ANGPTL4, BCAT1, BCKDHA, p-mTOR, and p-S6 in xenografts of OS tissue from the subcutaneous tumor nude mouse model (**C**, scale bars, 25 μm) (*N* = 3, ns *p* > 0.05, *p < 0.05, **p < 0.01, ***p < 0.001 vs. the control group).

Based on the in vitro results we obtained, the downregulation of ANGPTL4 expression led to the accumulation of BCAAs in cells by enhancing the activities of BCATs, which triggered mTOR signaling pathway, and ultimately promoted the proliferation of OS cells. To examine this signaling axis in vivo, we then generated a subcutaneous implantation tumor model in nude mice. Briefly, 1 × 10^6^ cells were injected into the nude mouse flank. When the longest diameter of the largest tumor reached 200 mm, the mice were sacrificed and the tumors were excised, measured and recorded. After the samples were embedded and cut into sections, the OS cell implants were detected with antibodies against ANGPTL4, BCAT1, BCKDHA, p-mTOR, and p-S6. The intensities of the IHC staining of these proteins were analyzed (Fig. 7A).

As shown in Fig. 7B, the growth of the OS implants was significantly promoted in the MNNG-siA4 cells (the ratio of MNNG-siA4 vs. siControl was 3.01 ± 1.40), while the MNNG cells overexpressing *ANGPTL4* resulted in attenuated growth (the ratio of MNNG-A4 vs. A4Control was 0.40 ± 0.30). Then, we detected ANGPTL4/BCAA/mTOR signaling in the OS tissues obtained from these nude mouse models by IHC staining. As shown in Fig. 7C, downregulated *ANGPTL4* expression in the OS tissue resulted in high expression levels of these proteins (the ratio of MNNG-siA4 vs. siControl for ANGPTL4, BCAT1, BCKDHA, p-mTOR, and p-S6 was 0.87 ± 0.03, 1.07 ± 0.02, 1.14 ± 0.02, 1.57 ± 0.15, and 1.07 ± 0.01, respectively). In contrast, in the tissue with upregulated ANGPTL4 expression, the expression patterns of these key proteins showed an opposite pattern except BCAT1 (the ratio of MNNG-A4 vs. A4Control for ANGPTL4, BCAT1, BCKDHA, p-mTOR, and p-S6 was 1.28 ± 0.02, 0.92 ± 0.04, 0.83 ± 0.02, 0.92 ± 0.03, and 0.90 ± 0.01, respectively) (Fig. 7D).

## Discussion

BCAAs, essential amino acids for humans, are important components of metabolic regulation. BCAAs are crucial in protein synthesis and energy supply, which provide a strong foundation for unrestricted division and durable growth of tumor cells [15, 19]. However, it seems not all tumor cells possess the same pattern of BCAA metabolism. An increased level of circulating BCAAs as a result of the protein breakdown or BCKA amination was observed in cancer patients with pancreatic adenocarcinoma and leukemia [20, 21]. These results do not indicate that the BCAA level is always elevated in cancer patients. Indeed, a decrease in circulating BCAAs caused by increased tumor cell uptake and breakdown was found in lung tumor cells [22]. Mayers et al. attributed these distinct phenotypes to differences in mutations and the origin of cancer tissues [22]. Furthermore, BCAAs were recently identified as an upstream signal input of the mTOR signaling pathway, which activates the pathway regulating the growth and proliferation of cancer cells [17, 23–25]. Several recent studies have discovered that the accumulation of BCAAs in cancer cells due to the reduction in catabolism may enhance the activity of mTOR signaling pathway and promote cancer progression [20, 21, 26–28]. However, little is known about the functions of BCAAs in the progression of OS.

**Fig. 8:**
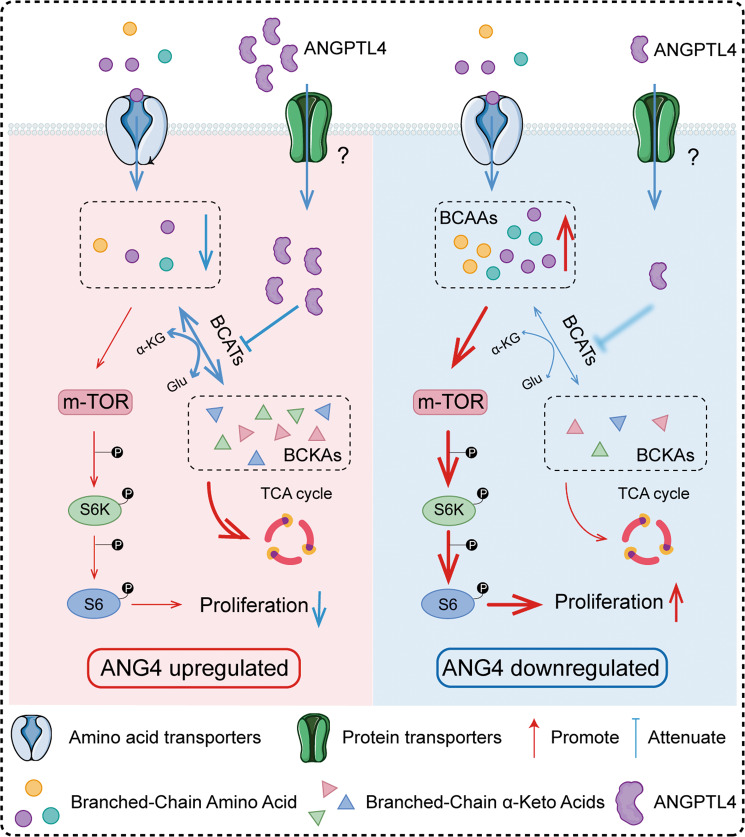
ANGPTL4 negatively regulates OS cell growth by remodeling BCAA metabolism. Diagram illustrating one mechanism that illustrates how ANGPTL4 influences OS cell progression through the ANGPTL4/BCAAs/mTOR axis.

In our present study, we demonstrated that ANGPLT4 triggers the BCAA/mTOR signaling axis in OS cells. Knockdown of *ANGPTL4* in OS cell lines changed the metabolism of BCAAs and then enhanced the accumulation of BCAAs in these cells. This change led to the activation of the mTOR signaling pathway, which resulted in the promotion of OS cell growth (Fig. 8). Our findings provided further evidence for the theory that BCAA metabolism is involved in the progression of various tumors and confirmed that BCAA metabolism can be subject to regulation by ANGPTL4.

Although the role of ANGPTL4 in the metabolic regulation was compelling, the impacts of ANGPTL4 on tumor progression were confusing. Previous studies have shown that ANGPTL4 had higher expression in tumor patients and induced a malignant phenotype of tumor cells, i.e., proliferation, migration, and drug resistance [10, 12, 29, 30]. However, some subsequent studies demonstrated that ANGPTL4 showed an antiangiogenic effect as well as antitumor cell invasion and migration effects during tumor progression. Thus, this molecule should be regarded as a tumor suppressor and a favorable prognostic marker of patients [11, 31–33]. In a previous OS study, Zhang et al. showed that ANGPTL4 promoted tumor progression, which is inconsistent with our findings [13]. However, there was no more detailed mechanism research that can be referred to in Zhang’s study, which remains no definite explanation for these contradictory phenomena to date. Mechanically, ANGPTL4 can be processed and cleaved into two main functional domains, the *N*-terminal fragment (nANGPTL4) and the *C*-terminal fragment (cANGPTL4) [34, 35]. The difference in function of the full-length ANGPTL4 (flANGPTL4) and the cleaved form may contribute to the contrary experimental results. Except for being directly cleaved, as a secreted glycoprotein, ANGPTL4 has diverse post-translational modifications, which affect the functions of ANGPTL4. For example, abnormal sialylation of ANGPTL4 was observed in nephrotic syndrome [36]. Furthermore, available data have suggested that the different experimental models, different microenvironments, and even different cell lines could be the reasons why discrepancies were observed in different studies.

As mentioned above, more research is needed to determine the overall mechanism by which ANGPTL4 affects tumors, but the present results revealed major components of the mechanism. Recent research has shed new light on lipid metabolism and progression in tumors. Pascual et al. noted that a high-fat diet promoted the metastasis of oral squamous cell carcinoma and melanoma in an animal model through epigenetic regulation [37]. In addition, the immune microenvironment of the intestinal tract was validated and could be regulated by a high-fat diet, which enhanced intestinal tumorigenesis [38]. Interestingly, previous studies found a mutual effect between BCAAs and lipid metabolism [39, 40]. Supplementation with BCAAs in a diet-induced obese mouse model resulted in significant hepatic metabolic dysfunction that promoted gluconeogenesis and inhibited lipogenesis [23]. In contrast, the suppression of BCAA catabolism was also observed under high-fat conditions, and the expression of BCAA catabolic enzymes was reduced in mice fed a high-fat diet [41]. Conceivably, although the underlying mechanism is unknown to date, the interaction between BCAA metabolism and lipid metabolism could affect tumor progression. According to our results, ANGPTL4 serves as not only a lipid regulator but also a bridge between BCAA metabolism and lipid metabolism.

Although our current results have partly clarified the organization and relationship of ANGPTL4, BCAA metabolism, and the mTOR signaling pathways in the progression of OS, the precise mechanisms involved in this loop remain unclear and need to be further studied. In particular, we demonstrated how ANGPTL4 regulates the metabolism of BCAAs, thereby regulating tumor progression of OS through the activation of mTOR signaling pathway, but we do not know exactly how various receptors are involved. The precise mechanisms involved in this loop, especially the cascade between ANGPTL4 and the BCATs, are unclear and need to be further studied. Furthermore, the present results demonstrated the important role of ANGPTL4 in the progression of OS. However, no more details were obtained, and it is still unclear whether it is the flANGPTL4, the truncated ANGPTL4 (nANGPTL4, cANGPTL4) or the cooperation of all three molecules that plays a role in tumor progression. Thus, additional studies are needed to further clarify the exact mechanism underlying the BCAA metabolic alteration after ANGPTL4 regulation during OS progression.

In summary, we discovered that low levels of ANGPTL4 regulated the metabolism of BCAAs to activate the mTOR signaling pathway, leading to accelerated proliferation in OS, as presented in Fig. 8. ANGPTL4 may be a reasonable and not previously reported bridge that links BCAA metabolism and lipid metabolism to promote OS progression. Given that overexpression of ANGPTL4 reduced the progression of OS, increasing the expression of ANGPTL4 in tumor cells of patients with OS may be a promising therapeutic strategy for OS in the future.

## Materials and methods

### Clinical samples

The clinical specimens, including OS tissue and normal cancellous bone tissue, were collected from the Department of Bone Oncology and Department of Emergency, Shanghai Jiao Tong University Affiliated Sixth People’s Hospital. Information on patients in both the OS group and the control group is shown in Supplementary Tables 1 and 2. Ethical approval was obtained from the Ethics Committee of Shanghai Sixth People’s Hospital (YS-2016-064, February 24, 2016).

### RNA extraction and real-time quantitative PCR (RT-qPCR)

Total RNA from both clinical specimens and cell lines was extracted by TRIzol reagent (Invitrogen, USA) and then reverse transcribed to cDNA according to the instructions of the cDNA Synthesis Kit (Invitrogen, USA). The relative gene expression levels were measured on an ABI Prism 7900HT real-time system (Applied Biosystems) and calculated by the 2^−ΔΔCt^ approach. All primers are shown in Supplementary Table 3.

### Western blot analysis and reagents

Total proteins were extracted by RIPA solution (EpiZyme, PC102) according to standard procedures. Then, the collected proteins were separated by electrophoresis and transferred to a PVDF membrane. The nonspecific binding sites of the PVDF membrane were blocked with 5% milk at room temperature (RT) for 60 min. Target proteins were detected by incubation in the primary antibody solution at 4 °C overnight. Finally, protein bands were detected by chemiluminescence. All primary antibodies are shown in Supplementary Table 4.

### Cell culture

Human MNNG/HOS (MNNG is used), U2OS, and MG63 cells were purchased from the American Type Culture Collection. Human bone marrow stromal cells (BMSCs) were harvested from patients with open fractures who underwent debridement at Shanghai Jiao Tong University Affiliated Sixth People’s Hospital. The culture media for MNNG, MG63, and U2OS cells and BMSCs were DMEM (Corning, USA), RPMI 1640 (Corning, USA), and α-MEM (Corning, USA), respectively. All culture media contained 10% fetal bovine serum (Gibco, USA). All cells were cultured at 37 °C in an atmosphere containing 5% CO_2_.

### Stable cell line construction

For stable cell line construction, HEK293T cells were co-transfected with lentivirus packing vectors and LV shuttle plasmids containing full-length ANGPTL4 and siRNA against ANGPTL4. Forty-eight hours later, the supernatant containing lentivirus was collected by centrifugation at 400 × *g* for 10 min and purified, and the titer was determined. Then, the lentivirus was added to the culture medium of MNNG cells at a multiplicity of infection of 10.0 to infect the cells. After 72 h, the culture medium was changed to a new medium that contained puromycin at a concentration of 1.0 µg/ml to select the positive cells. Finally, stable cells overexpressing (MNNG-A4) and with knockdown of ANGPTL4 (MNNG-siA4) were verified by RT-qPCR and Western blot. All the information on the vector and sequences of full-length ANGPTL4 and siRNA against ANGPTL4 is provided in Supplementary Table 5.

### Cell proliferation assay

Real-time cellular analysis (RTCA) (ACEA Biosciences, USA) was used to evaluate cell proliferation [16]. First, the baseline value was measured in 100 μl of culture medium preincubated at 37 °C in a cell incubator for 1 h. Then, the cells were seeded in wells at a density of 2000 cells per well. The attachment and proliferation of cells were measured by the RTCA system for 6 and 168 h, respectively.

### Colony formation assay

The cells were seeded into a 6-well plate at 1000 cells per well. After incubation for 14 days, the cells were fixed with 4% paraformaldehyde and then immersed in crystal violet for half an hour. The cells in each well were photographed and recorded, and colonies containing more than 50 cells were counted by ImageJ software.

### Subcutaneous tumor model

Female nude mice at 4–6 weeks of age were purchased from the Laboratory Animal Research Center of the Shanghai Sixth People’s Hospital, and all operations were approved by the Animal Research Committee of the Shanghai Sixth People’s Hospital. After anesthesia with pentobarbital sodium, 200 μl of cell suspension containing 1 × 10^6^ cells were injected into the nude mouse flank [42]. Tumors were measured by researchers until the longest diameter of the largest tumor reached 200 mm. The volume of tumors was calculated as length (mm) × width (mm)^2^/2.

### RNA-seq and analysis

TRIzol (Invitrogen, USA) was used to extract total RNA from the MNNG-A4, MNNG-siA4, and control cell lines. Then, standard guidelines were followed to construct paired-end libraries with a TruSeq™ RNA Sample Preparation Kit (Illumina, USA). The mRNA was cleaved into small pieces and reverse transcribed into first strand cDNA. Then, DNA polymerase I and RNase H were used to generate second strand cDNA. These cDNAs then underwent the addition of a single “A” base and ligation of the adapters. The products were purified and enriched with PCR to create the final cDNA library. Library construction and sequencing were performed by Sinotech Genomics Co., Ltd. (Shanghai, PRC). Differentially expressed genes were selected based on a false discovery rate <5% and changed expression higher than 1.5-fold or lower than 0.67-fold. All cell lines were tested three times. The raw RNA-seq data has been uploaded to the NCBI SRA database. The SRA accession number: PRJNA822527.

### BCAA assay

Cells (2 × 10^6^) were harvested from a T75 cell culture flask. The cells were lysed, and the levels of BCAAs were measured by a BCAA assay kit according to the manufacturer’s instructions (Sigma-Aldrich, USA). Briefly, cells were lysed in 100 ml of cold BCAA assay buffer to obtain the lysate, 10 μl of lysate was added to a 96-well plate, and BCAA buffer was added to bring the volume to 50 ml. Next, 50 μl reaction mixes containing 46 μl of assay buffer, 2 μl of BCAA enzyme mix, and 2 μl of WST substrate mix were added to each well. The blank wells contained the cell lysate, BCAA assay buffer, and substrate mix but omitted the enzyme. Then, the reaction was incubated for 30 min at RT, and the absorbance was measured at 450 nm (A450). The blank absorbances were subtracted from the lysate absorbances. A standard curve was generated through the above method, but the cell lysate was replaced with a leucine standard. Each sample was measured in duplicate.

### Immunohistochemical (IHC) analysis

The clinical OS specimens and OS tissues excised from the subcutaneous tumor model were embedded in paraffin, cut into 4 μm sections and deparaffinized. The sections were blocked with 5% bovine serum albumin at 37 °C for 30 min after antigen retrieval. Next, specific primary antibodies were added to the samples and incubated overnight at 4 °C. Then, the cells were washed three times with PBS and incubated with HRP-linked anti-IgG at 37 °C for 30 min. The cells were washed with PBS again and stained with DAB for 10 min. Finally, the samples were counterstained, dehydrated, covered with cover glass and photographed with a DM6B system (Leica, BRD).

### Statistical analyses

The data were analyzed by SPSS 25.0 software and are presented as the mean ± SD. The differences between the experimental and control groups were analyzed by two-tailed Student’s *t* test, and the differences between the tumor tissues and nontumor tissues were analyzed by Welch’s *t* test. ns indicates *p* > 0.05, * indicates *p* < 0.05, ** indicates *p* < 0.01, and *** indicates *p* < 0.001.

## Supplementary information

Supplementary Fig. 1

Supplementary table 1

Supplementary table 2

Supplementary table 3

Supplementary table 4

Supplementary table 5

Original Data File

Original Data File

## Data Availability

The datasets used and/or analyzed during the current study are available from the corresponding author on reasonable request.

## References

[1] Gianferante DM, Mirabello L, Savage SA (2017). Germline and somatic genetics of osteosarcoma—connecting aetiology, biology and therapy. Nat Rev Endocrinol.

[2] Ritter J, Bielack SS (2010). Osteosarcoma. Ann Oncol: Off J Eur Soc Med Oncol.

[3] Kansara M, Teng MW, Smyth MJ, Thomas DM (2014). Translational biology of osteosarcoma. Nat Rev Cancer.

[4] Song BS, Seo J, Kim DH, Lim JS, Yoo JY, Lee JA (2014). Gemcitabine and docetaxel for the treatment of children and adolescents with recurrent or refractory osteosarcoma: Korea Cancer Center Hospital experience. Pediatr Blood Cancer.

[5] Duffaud F, Egerer G, Ferrari S, Rassam H, Boecker U, Bui-Nguyen B (2012). A phase II trial of second-line pemetrexed in adults with advanced/metastatic osteosarcoma. Eur J Cancer.

[6] Santulli G (2014). Angiopoietin-like proteins: a comprehensive look. Front Endocrinol.

[7] Aryal B, Price NL, Suarez Y, Fernández-Hernando C (2019). ANGPTL4 in metabolic and cardiovascular disease. Trends Mol Med.

[8] La Paglia L, Listì A, Caruso S, Amodeo V, Passiglia F, Bazan V (2017). Potential role of ANGPTL4 in the cross talk between metabolism and cancer through PPAR signaling pathway. PPAR Res.

[9] Fernández-Hernando C, Suárez Y (2020). ANGPTL4: a multifunctional protein involved in metabolism and vascular homeostasis. Curr Opin Hematol.

[10] Chen JW, Luo YJ, Yang ZF, Wen LQ, Huang L (2018). Knockdown of angiopoietin-like 4 inhibits the development of human gastric cancer. Oncol Rep.

[11] Cai YC, Yang H, Wang KF, Chen TH, Jiang WQ, Shi YX (2020). ANGPTL4 overexpression inhibits tumor cell adhesion and migration and predicts favorable prognosis of triple-negative breast cancer. BMC Cancer.

[12] Tsai YT, Wu AC, Yang WB, Kao TJ, Chuang JY, Chang WC, et al. ANGPTL4 Induces TMZ Resistance of Glioblastoma by Promoting Cancer Stemness Enrichment via the EGFR/AKT/4E-BP1 Cascade. Int J Mol Sci. 2019;20:5625. 10.3390/ijms20225625 PMC6888274 31717924

[13] Zhang T, Kastrenopoulou A, Larrouture Q, Athanasou NA, Knowles HJ (2018). Angiopoietin-like 4 promotes osteosarcoma cell proliferation and migration and stimulates osteoclastogenesis. BMC Cancer.

[14] Zhang S, Zeng X, Ren M, Mao X, Qiao S (2017). Novel metabolic and physiological functions of branched chain amino acids: a review. J Anim Sci Biotechnol.

[15] Peng H, Wang Y, Luo W (2020). Multifaceted role of branched-chain amino acid metabolism in cancer. Oncogene..

[16] Lebourgeois S, Fraisse A, Hennechart-Collette C, Guillier L, Perelle S, Martin-Latil S (2018). Development of a real-time cell analysis (RTCA) method as a fast and accurate method for detecting infectious particles of the adapted strain of hepatitis A virus. Front Cell Infect Microbiol.

[17] Wolfson RL, Chantranupong L, Saxton RA, Shen K, Scaria SM, Cantor JR (2016). Sestrin2 is a leucine sensor for the mTORC1 pathway. Science..

[18] Hu LY, Boxer PA, Kesten SR, Lei HJ, Wustrow DJ, Moreland DW (2006). The design and synthesis of human branched-chain amino acid aminotransferase inhibitors for treatment of neurodegenerative diseases. Bioorg Med Chem Lett.

[19] Ananieva EA, Wilkinson AC (2018). Branched-chain amino acid metabolism in cancer. Curr Opin Clin Nutr Metab care.

[20] Mayers JR, Wu C, Clish CB, Kraft P, Torrence ME, Fiske BP (2014). Elevation of circulating branched-chain amino acids is an early event in human pancreatic adenocarcinoma development. Nat Med.

[21] Hattori A, Tsunoda M, Konuma T, Kobayashi M, Nagy T, Glushka J (2017). Cancer progression by reprogrammed BCAA metabolism in myeloid leukaemia. Nature..

[22] Mayers JR, Torrence ME, Danai LV, Papagiannakopoulos T, Davidson SM, Bauer MR (2016). Tissue of origin dictates branched-chain amino acid metabolism in mutant Kras-driven cancers. Science..

[23] Zhao H, Zhang F, Sun D, Wang X, Zhang X, Zhang J (2020). Branched-chain amino acids exacerbate obesity-related hepatic glucose and lipid metabolic disorders via attenuating Akt2 signaling. Diabetes..

[24] Zhang S, Lin X, Hou Q, Hu Z, Wang Y, Wang Z (2021). Regulation of mTORC1 by amino acids in mammalian cells: a general picture of recent advances. Anim Nutr.

[25] Shao D, Villet O, Zhang Z, Choi SW, Yan J, Ritterhoff J (2018). Glucose promotes cell growth by suppressing branched-chain amino acid degradation. Nat Commun.

[26] Ericksen RE, Lim SL, McDonnell E, Shuen WH, Vadiveloo M, White PJ (2019). Loss of BCAA catabolism during carcinogenesis enhances mTORC1 activity and promotes tumor development and progression. Cell Metab.

[27] Gu Z, Liu Y, Cai F, Patrick M, Zmajkovic J, Cao H (2019). Loss of EZH2 reprograms BCAA metabolism to drive leukemic transformation. Cancer Disco.

[28] Lei MZ, Li XX, Zhang Y, Li JT, Zhang F, Wang YP (2020). Acetylation promotes BCAT2 degradation to suppress BCAA catabolism and pancreatic cancer growth. Signal Transduct Target Ther.

[29] Shen CJ, Chang KY, Lin BW, Lin WT, Su CM, Tsai JP (2020). Oleic acid-induced NOX4 is dependent on ANGPTL4 expression to promote human colorectal cancer metastasis. Theranostics..

[30] Nie D, Zheng Q, Liu L, Mao X, Li Z (2019). Up-regulated of angiopoietin-like protein 4 predicts poor prognosis in cervical cancer. J Cancer.

[31] Galaup A, Cazes A, Le Jan S, Philippe J, Connault E, Le Coz E (2006). Angiopoietin-like 4 prevents metastasis through inhibition of vascular permeability and tumor cell motility and invasiveness. Proc Natl Acad Sci USA.

[32] Ito Y, Oike Y, Yasunaga K, Hamada K, Miyata K, Matsumoto S (2003). Inhibition of angiogenesis and vascular leakiness by angiopoietin-related protein 4. Cancer Res.

[33] Okochi-Takada E, Hattori N, Tsukamoto T, Miyamoto K, Ando T, Ito S (2014). ANGPTL4 is a secreted tumor suppressor that inhibits angiogenesis. Oncogene..

[34] Ge H, Yang G, Huang L, Motola DL, Pourbahrami T, Li C (2004). Oligomerization and regulated proteolytic processing of angiopoietin-like protein 4. J Biol Chem.

[35] Lei X, Shi F, Basu D, Huq A, Routhier S, Day R (2011). Proteolytic processing of angiopoietin-like protein 4 by proprotein convertases modulates its inhibitory effects on lipoprotein lipase activity. J Biol Chem.

[36] Clement LC, Avila-Casado C, Macé C, Soria E, Bakker WW, Kersten S (2011). Podocyte-secreted angiopoietin-like-4 mediates proteinuria in glucocorticoid-sensitive nephrotic syndrome. Nat Med.

[37] Pascual G, Domínguez D, Elosúa-Bayes M, Beckedorff F, Laudanna C, Bigas C (2021). Dietary palmitic acid promotes a prometastatic memory via Schwann cells. Nature..

[38] Beyaz S, Chung C, Mou H, Bauer-Rowe KE, Xifaras ME, Ergin I (2021). Dietary suppression of MHC class II expression in intestinal epithelial cells enhances intestinal tumorigenesis. Cell Stem Cell.

[39] Gannon NP, Schnuck JK, Vaughan RA (2018). BCAA metabolism and insulin sensitivity—dysregulated by metabolic status?. Mol Nutr Food Res.

[40] Newgard CB (2012). Interplay between lipids and branched-chain amino acids in development of insulin resistance. Cell Metab.

[41] Estrada-Alcalde I, Tenorio-Guzman MR, Tovar AR, Salinas-Rubio D, Torre-Villalvazo I, Torres N (2017). Metabolic fate of branched-chain amino acids during adipogenesis, in adipocytes from obese mice and C2C12 myotubes. J Cell Biochem.

[42] Shimosato Y, Kameya T, Nagai K, Hirohashi S, Koide T, Hayashi H (1976). Transplantation of human tumors in nude mice. J Natl Cancer Inst.

