# Supplementary material for: ANGPTL4 negatively regulates the progression of osteosarcoma by remodeling branched-chain amino acid metabolism
Source: Cell Death Discov. 2022 Apr 23;8:225. doi: 10.1038/s41420-022-01029-x (PMC9035178; doi:10.1038/s41420-022-01029-x)
Supplement: Supplementary file 1 — Supplementary Fig. 1 [file 41420_2022_1029_MOESM1_ESM.docx]

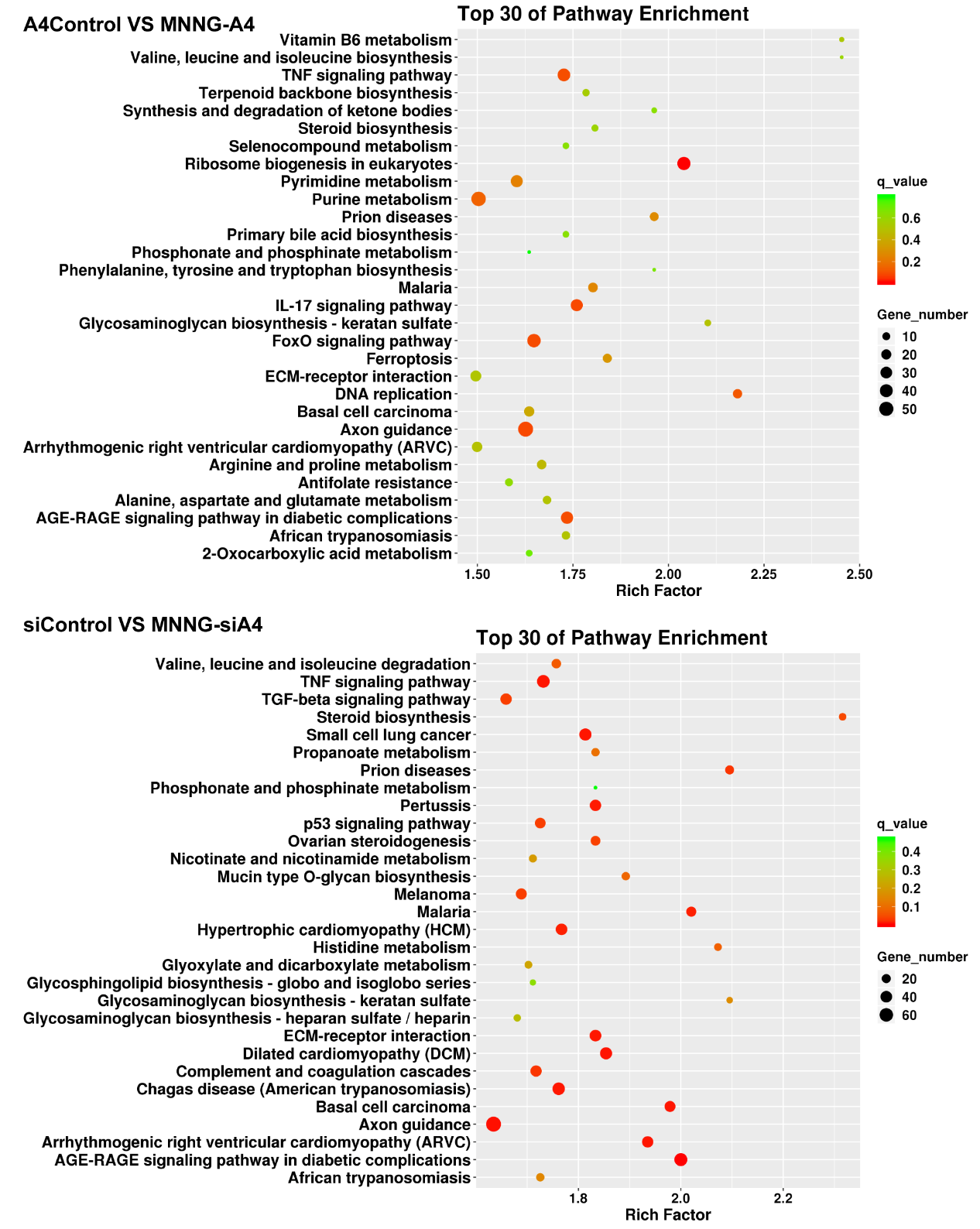


**Supplementary Fig. 1 Transcriptome analysis of MNNG-A4 cells, MNNG-siA4 cells and their control cells.** KEGG pathway enrichment analysis for DEGs in MNNG-A4 cells, MNNG-siA4 cells, and their control cells.
