# Supplementary material for: ANGPTL4 negatively regulates the progression of osteosarcoma by remodeling branched-chain amino acid metabolism
Source: Cell Death Discov. 2022 Apr 23;8:225. doi: 10.1038/s41420-022-01029-x (PMC9035178; doi:10.1038/s41420-022-01029-x)
Supplement: Supplementary file 2 — Supplementary table 1 [file 41420_2022_1029_MOESM2_ESM.docx]

**Supplementary table 1.** The information of patients in osteosarcoma group

| **NO.** | **AGE (YEARS)** | **GENDER** | **PRIMARY TUMOR SITE** | **DISEASE AT DIAGNOSIS** | **METASTASIS SITE** |
| --- | --- | --- | --- | --- | --- |
| 01 | 49 | Male | Left pelvis | Metastatic | Vertebral (T9, T11) |
| 02 | 36 | Male | Left distal radius | Non-metastatic | no |
| 03 | 17 | Male | Left proximal tibia | Non-metastatic | no |
| 04 | 29 | Female | Middle left fibula | Non-metastatic | no |
| 05 | 23 | Male | Left proximal tibia | Non-metastatic | no |
| 06 | 10 | Male | Right proximal tibia | Non-metastatic | no |
| 07 | 60 | Female | Right pelvis | Metastatic | Vertebral (T8) |
| 08 | 54 | Female | Left distal tibia | Non-metastatic | no |
| 09 | 14 | Female | Left proximal femur | Non-metastatic | no |
| 10 | 21 | Male | Left distal femur | Non-metastatic | no |
| 11 | 30 | Male | Right proximal fibula | Non-metastatic | no |
| 12 | 13 | Female | Left distal femur | Metastatic | Lung |
| 13 | 58 | Male | Left proximal humerus | Non-metastatic | no |
| 14 | 19 | Female | Right distal femur | Non-metastatic | no |
| 15 | 9 | Male | Right proximal fibula | Non-metastatic | no |
