# Supplementary material for: ANGPTL4 negatively regulates the progression of osteosarcoma by remodeling branched-chain amino acid metabolism
Source: Cell Death Discov. 2022 Apr 23;8:225. doi: 10.1038/s41420-022-01029-x (PMC9035178; doi:10.1038/s41420-022-01029-x)
Supplement: Supplementary file 3 — Supplementary table 2 [file 41420_2022_1029_MOESM3_ESM.docx]

**Supplementary table 2.** The information of patients in control group

| **NO.** | **AGE** | **GENDER** | **SAMPLE SITE** | **DISEASE AT DIAGNOSIS** | **OPERATION** |
| --- | --- | --- | --- | --- | --- |
| 01 | 22 | Male | Left femur | Open fractures | Debridement |
| 02 | 47 | Female | Left tibia | Open fractures | Debridement |
| 03 | 36 | Male | Left tibia | Open fractures | Debridement |
| 04 | 31 | Female | Right radius | Open fractures | Debridement |
| 05 | 60 | Male | Left tibia | Open fractures | Debridement |
