# Supplementary material for: ANGPTL4 negatively regulates the progression of osteosarcoma by remodeling branched-chain amino acid metabolism
Source: Cell Death Discov. 2022 Apr 23;8:225. doi: 10.1038/s41420-022-01029-x (PMC9035178; doi:10.1038/s41420-022-01029-x)
Supplement: Supplementary file 4 — Supplementary table 3 [file 41420_2022_1029_MOESM4_ESM.docx]

**Supplementary table 3.** The primer sequences used for RT-PCR analysis

| **Gene name** | **Forward Primer Sequence (5’-3’)** | **Reverse Primer Sequence (5’-3’)** |
| --- | --- | --- |
| ANGPTL4 | GATGGCTCAGTGGACTTCAACC | TGCTATGCACCTTCTCCAGACC |
| AHU | TTGCAGCAATAGATGGACTCG | CAGCCCATTTTTGCAGAGG |
| AOX-1 | ATGCCTGTCTGATTCCATCT | CATGACACTTGGCAATCCTCT |
| ALDH6A1 | GGCAGACACTTCAGTATTAAGCC | AGAGGCAGACGGTAGGAATAAA |
| HMGCL | GTGTCTCCTAAGTGGGTTCCC | TGGGTAGTTGATGCCAGGAAA |
| BCKDHA | GGTGTGCTGATGTATCGGGAC | CTTGCAGCCGTAGTGGACA |
| BCKDHB | GATTTGGAATCGGAATTGCGG | CAGAGCGATAGCGATACTTGG |
| IL4I1 | GCCAAGACCCCTTCGAGAAAT | CCGATCCTGTTATCTGCCTCC |
| SDSL | GACGGCTGGGAGAATGTCC | ATGGCCGCATTGAAGCAGT |
| ACADS | CGGCAGTTACACACCATCTAC | GCAATGGGAAACAACTCCTTCTC |
| ABAT | TGGGTTGCTTAGCGACCAC | TCCAGAGGGTATTTCAGCCGT |
