# Supplementary material for: ANGPTL4 negatively regulates the progression of osteosarcoma by remodeling branched-chain amino acid metabolism
Source: Cell Death Discov. 2022 Apr 23;8:225. doi: 10.1038/s41420-022-01029-x (PMC9035178; doi:10.1038/s41420-022-01029-x)
Supplement: Supplementary file 5 — Supplementary table 4 [file 41420_2022_1029_MOESM5_ESM.docx]

**Supplementary table 4.** The antibody used for western blot analysis

| **Gene Name** | **Manufacturer** | **Lot. No** | **Source** | **Dilution** |
| --- | --- | --- | --- | --- |
| ANGPT4 | Affinity | DF9209 | Rabbit | 1:1000 |
| BCKDHA | Affinity | DF13663 | Rabbit | 1:1000 |
| IL4I1 | Affinity | DF13745 | Rabbit | 1:1000 |
| HMGCL | Affinity | DF13063 | Rabbit | 1:1000 |
| AOX1 | Affinity | DF3756 | Rabbit | 1:1000 |
| RPS6 | Affinity | AF7831 | Rabbit | 1:1000 |
| phospho-RPS6 | Affinity | AF8310 | Rabbit | 1:1000 |
| p70 S6 kinase | Affinity | AF6226 | Rabbit | 1:1000 |
| phospho-p70 S6 kinase | Affinity | AF3228 | Rabbit | 1:1000 |
| m-TOR | Cell Signaling Technology | #2983 | Rabbit | 1:1000 |
| phospho-mTOR | Cell Signaling Technology | #5536 | Rabbit | 1:1000 |
| GAPDH | Affinity | AF7021 | Rabbit | 1:1500 |
| β-Tubulin | Affinity | AF7011 | Rabbit | 1:1500 |
