# Supplementary material for: ANGPTL4 negatively regulates the progression of osteosarcoma by remodeling branched-chain amino acid metabolism
Source: Cell Death Discov. 2022 Apr 23;8:225. doi: 10.1038/s41420-022-01029-x (PMC9035178; doi:10.1038/s41420-022-01029-x)
Supplement: Supplementary file 7 — Original Data File [file 41420_2022_1029_MOESM7_ESM.pdf]

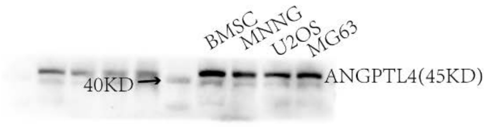

ANGPTL4_Fig.1

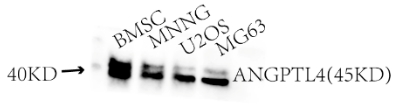

ANGPTL4_Fig.1_1

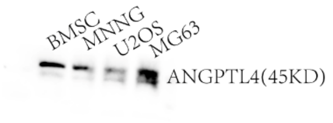

ANGPTL4_Fig.1_2

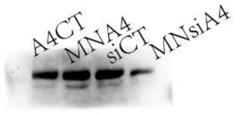

ANGPTL4_Fig.2

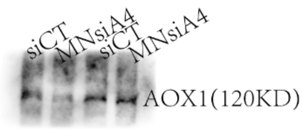

AOX1_5μm

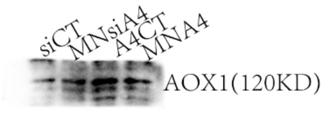

AOX1_a4 & si4

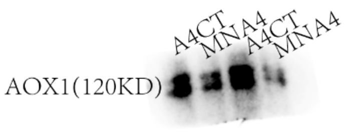

AOX1_a4

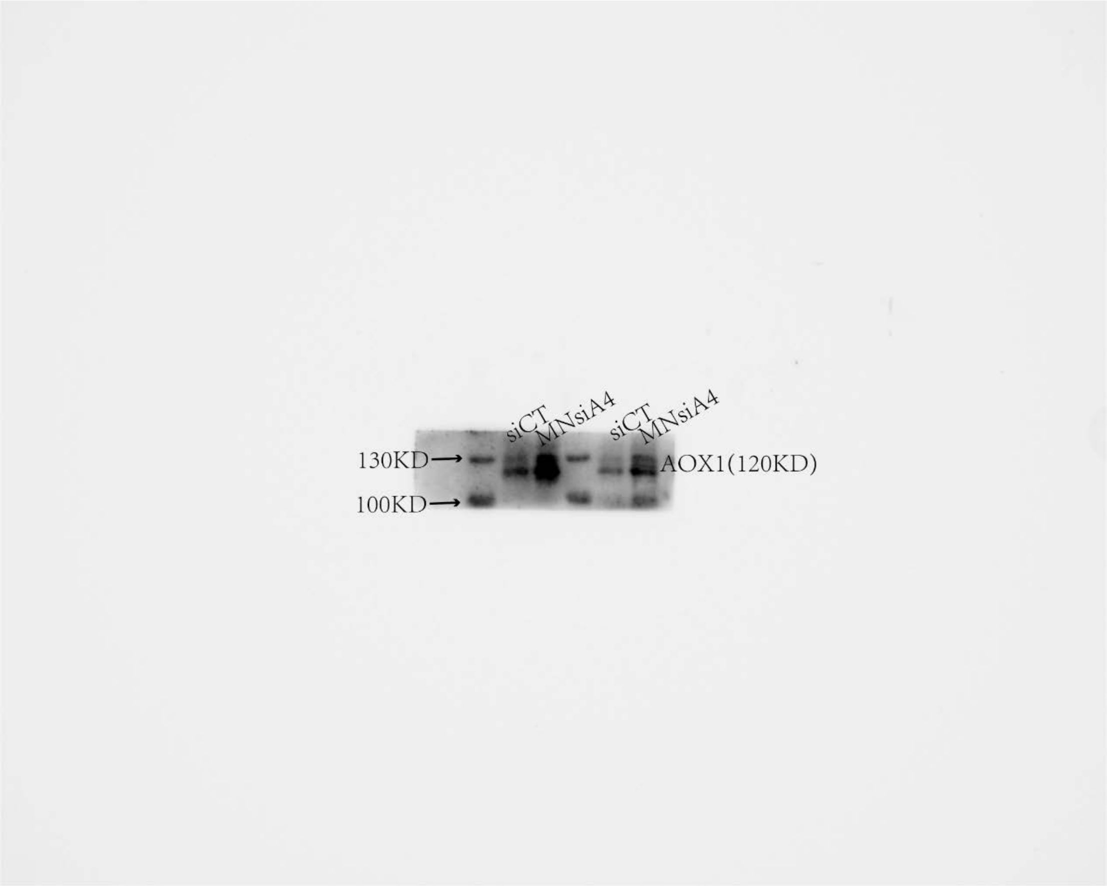

AOX1_sia4

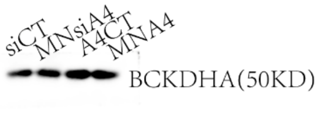

A4 & siA4

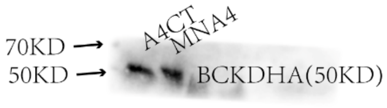

BCKDHA_A4

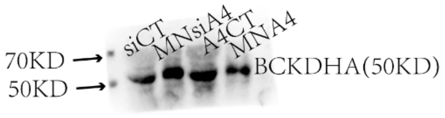

BCKDHA_sia4 & a4

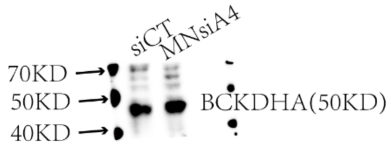

BCKDHA_sia4

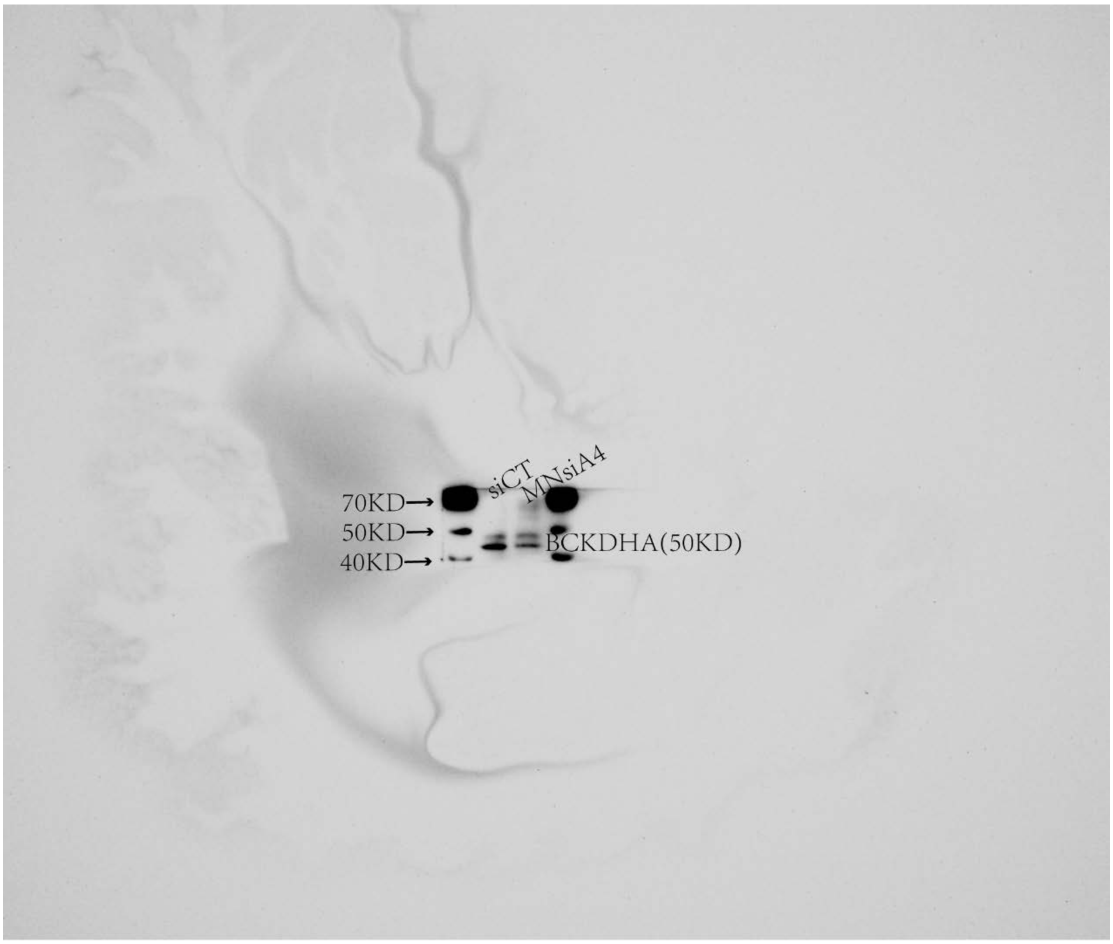

BCKDHA_sia4_5μm

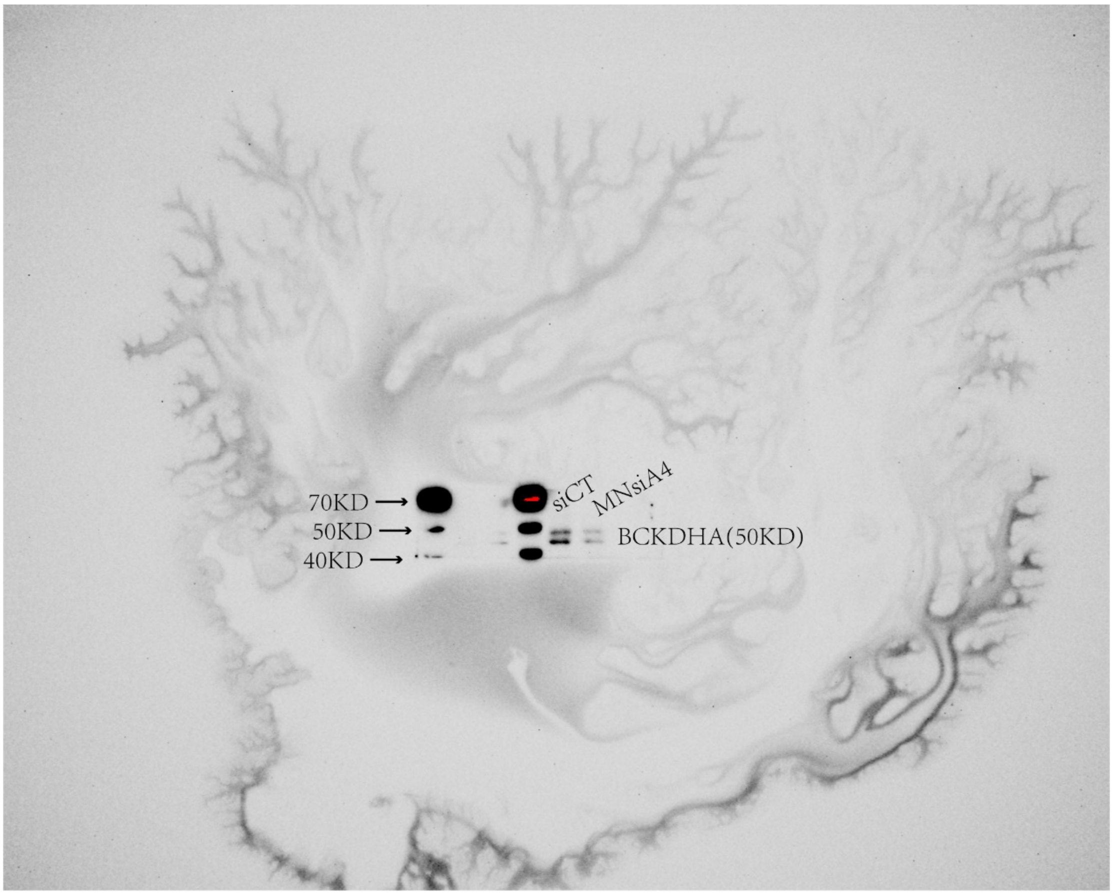

BCKDHA_sia4_5μm_1

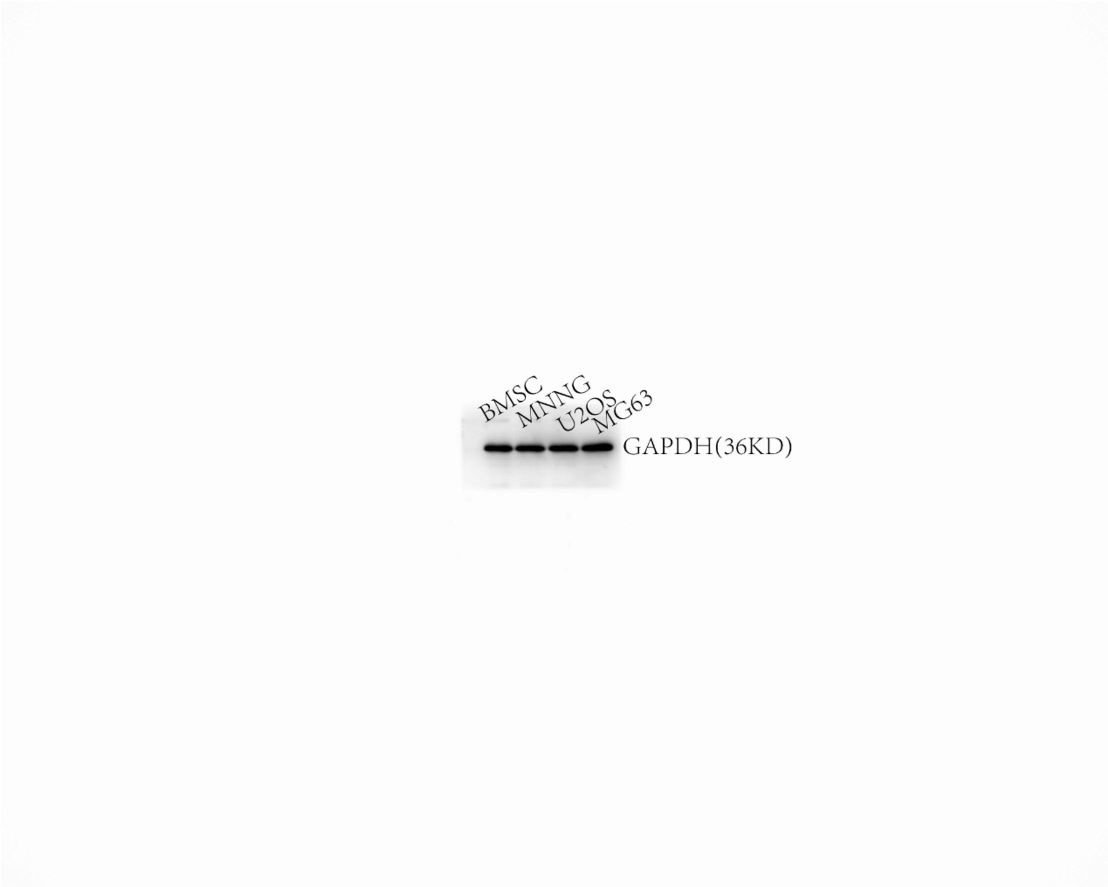

GAPDH_Fig.1

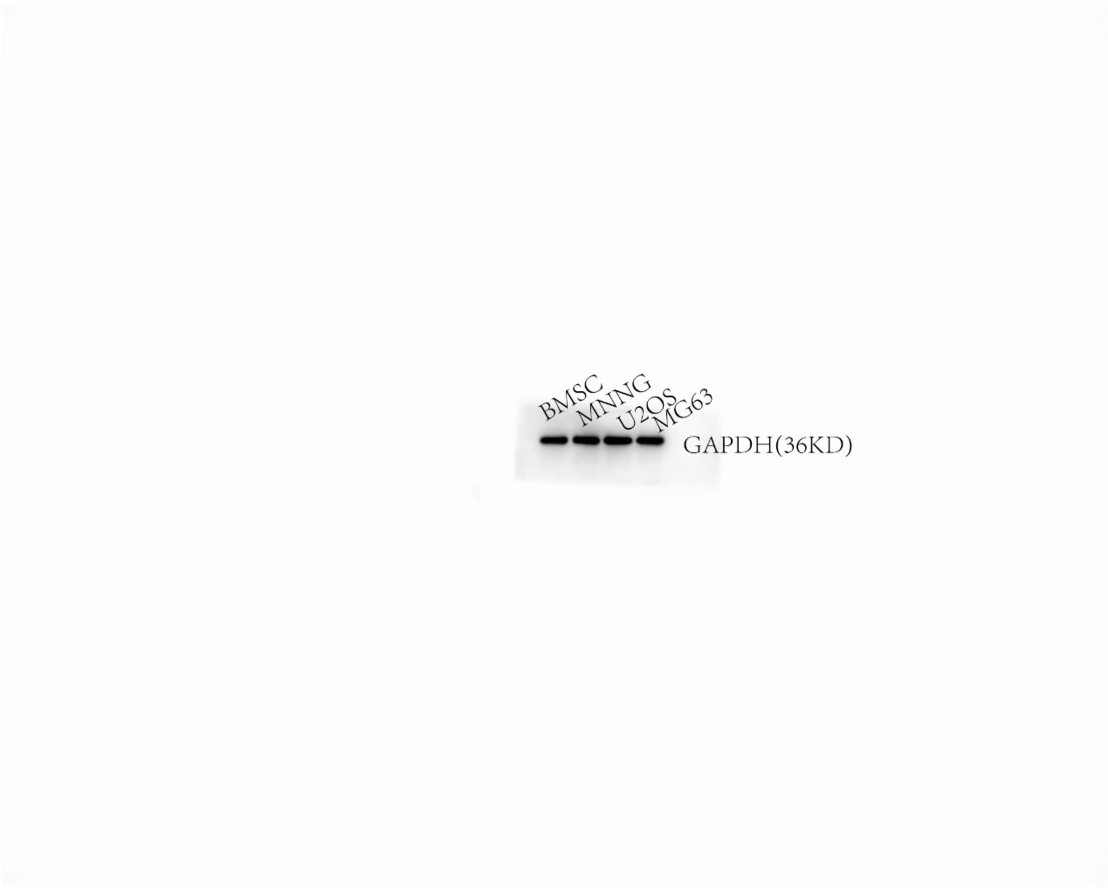

GAPDH_Fig.1_1

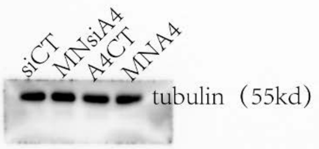

tubulin_Fig.4_1

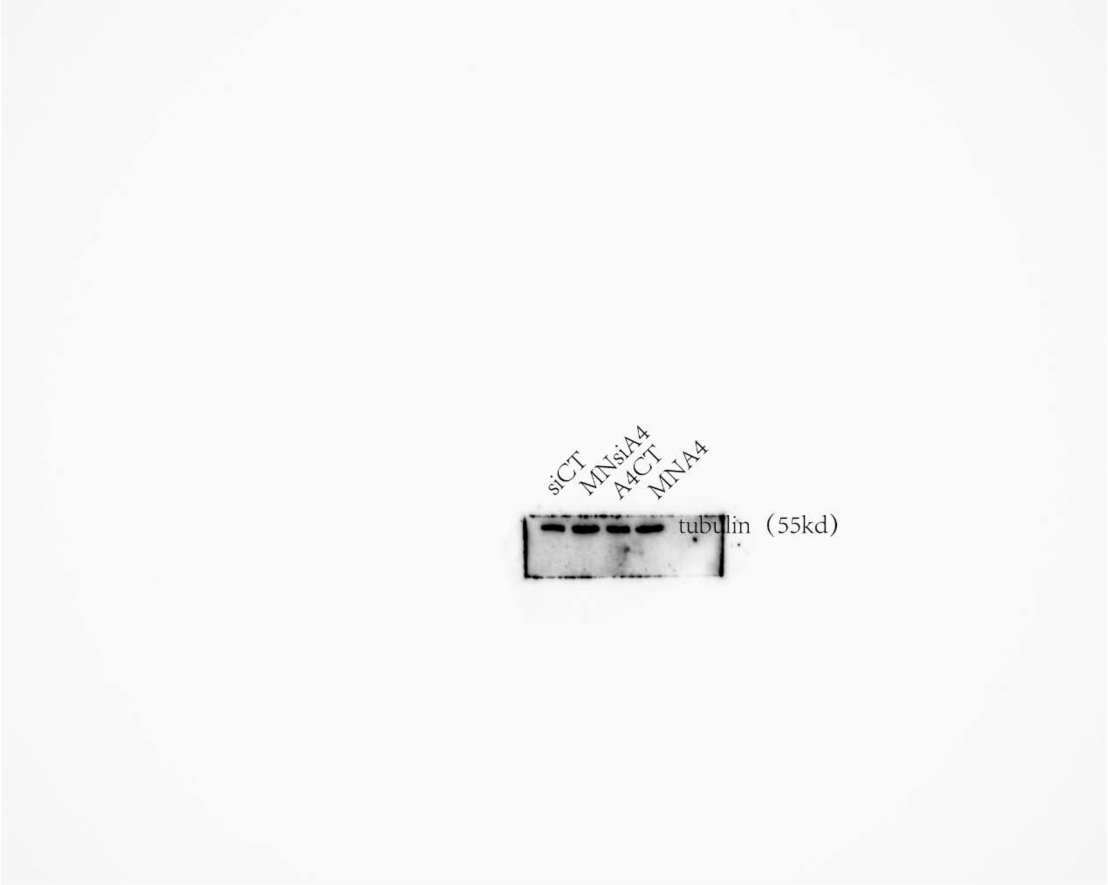

tubulin_Fig.4_2

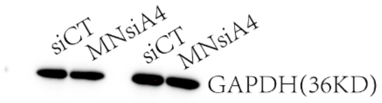

GAPDH_Fig.5

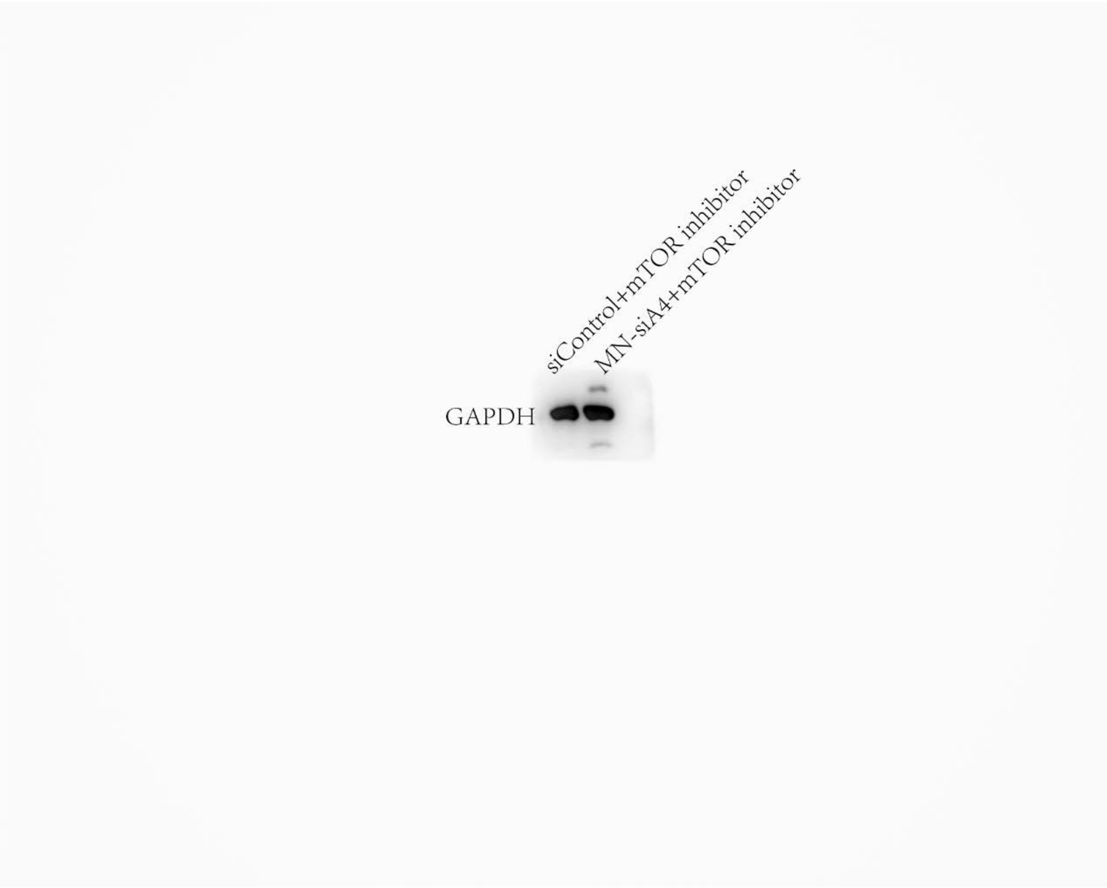

GAPDH_Fig.6

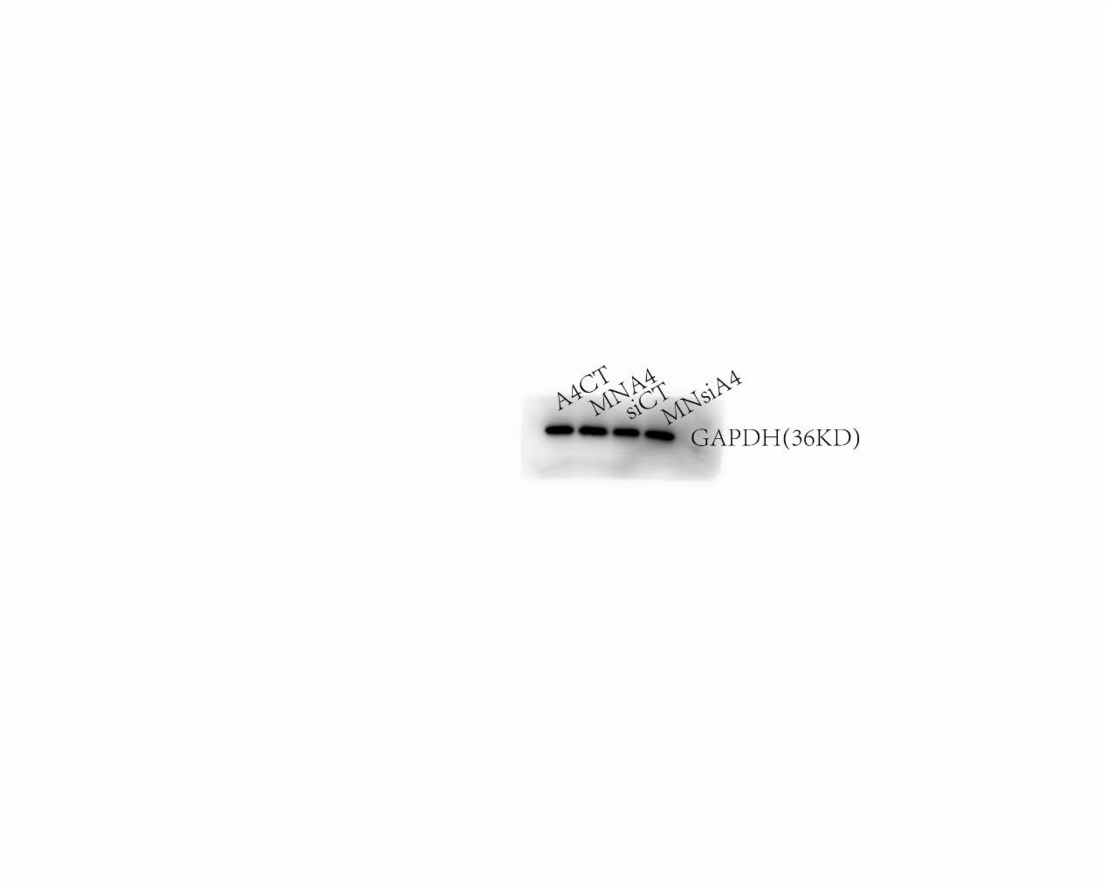

GAPDH_Fig.2

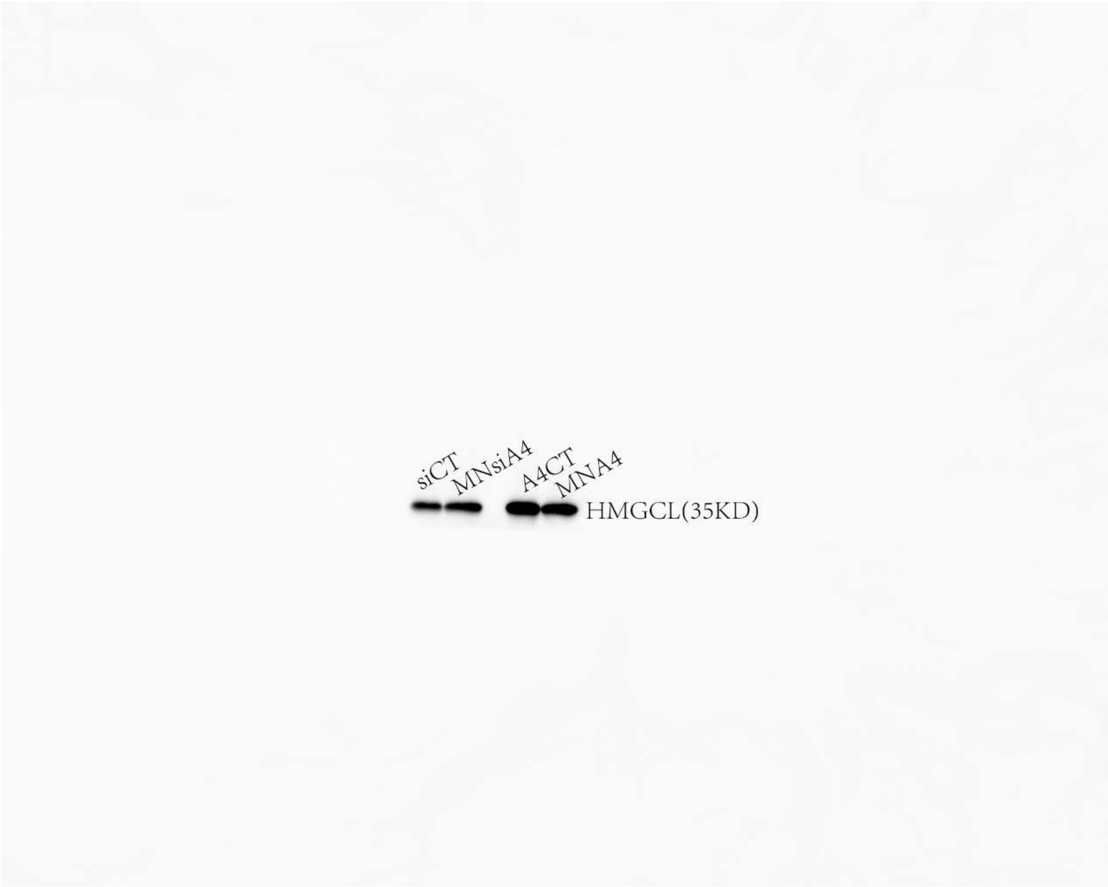

HMGCL_a4 & sia4

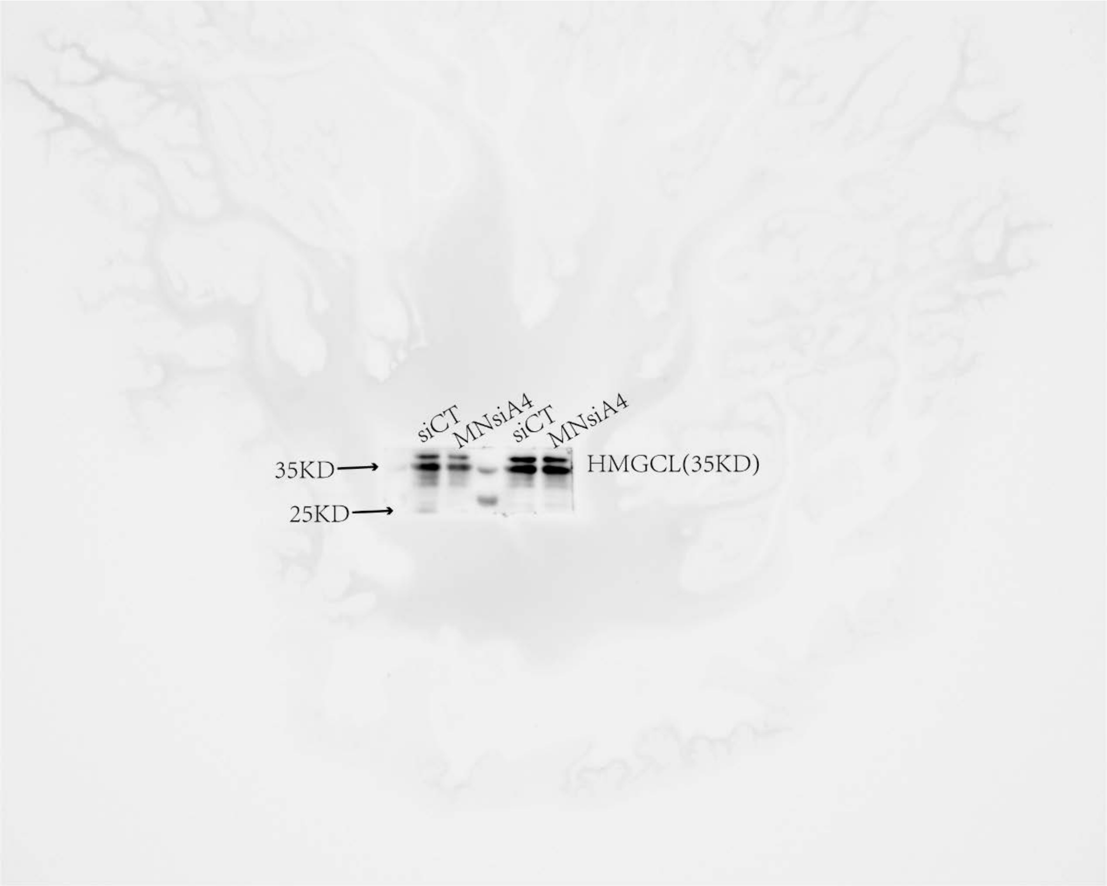

HMGCL_5μm

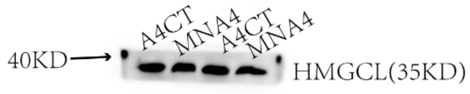

HMGCL_a4

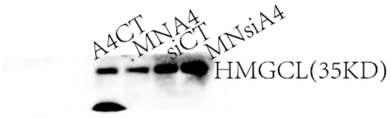

HMGCL_sia4

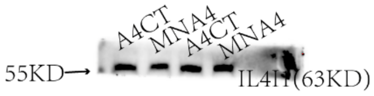

IL4I1_ a4

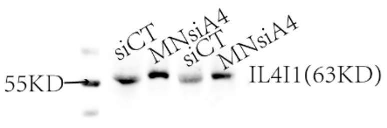

IL4I1_ sia4

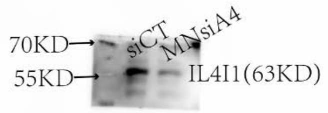

IL4I1_5μm

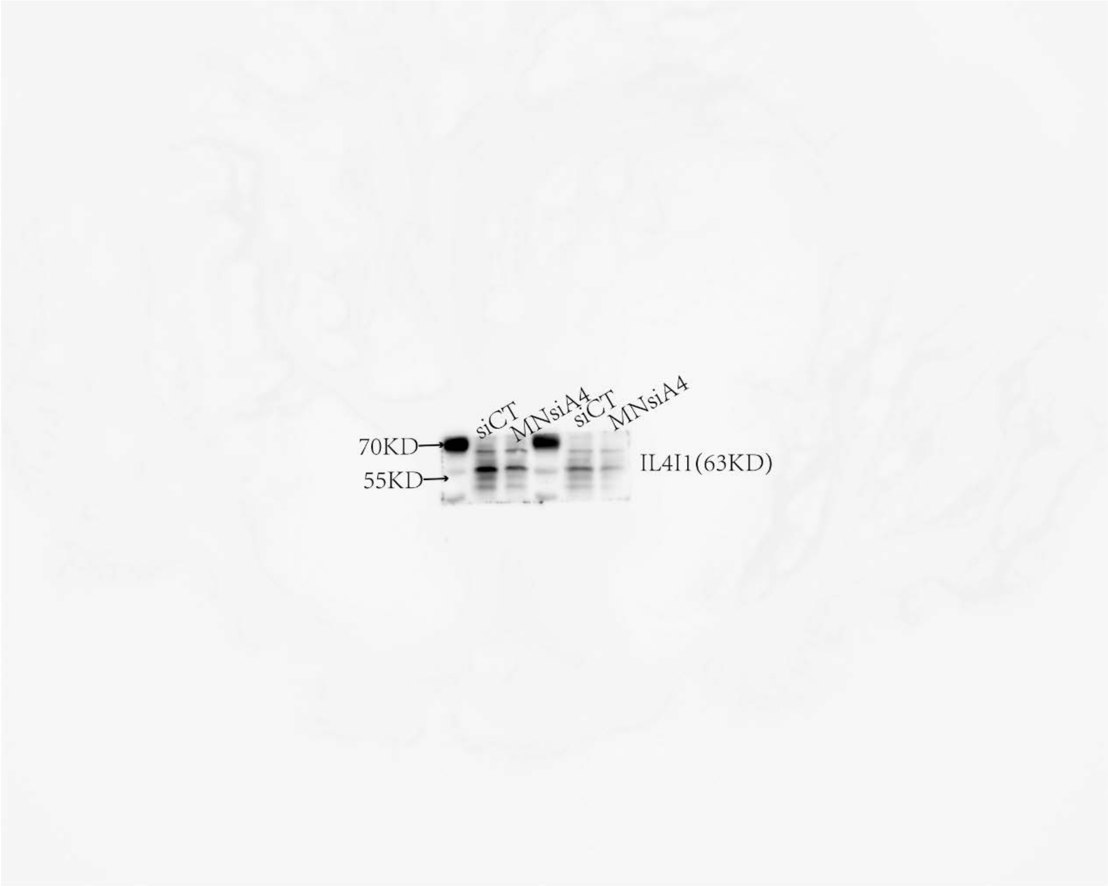

IL4I1_5μm_1

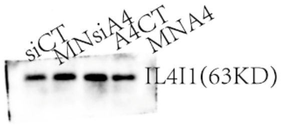

IL4I1 a4 & sia4

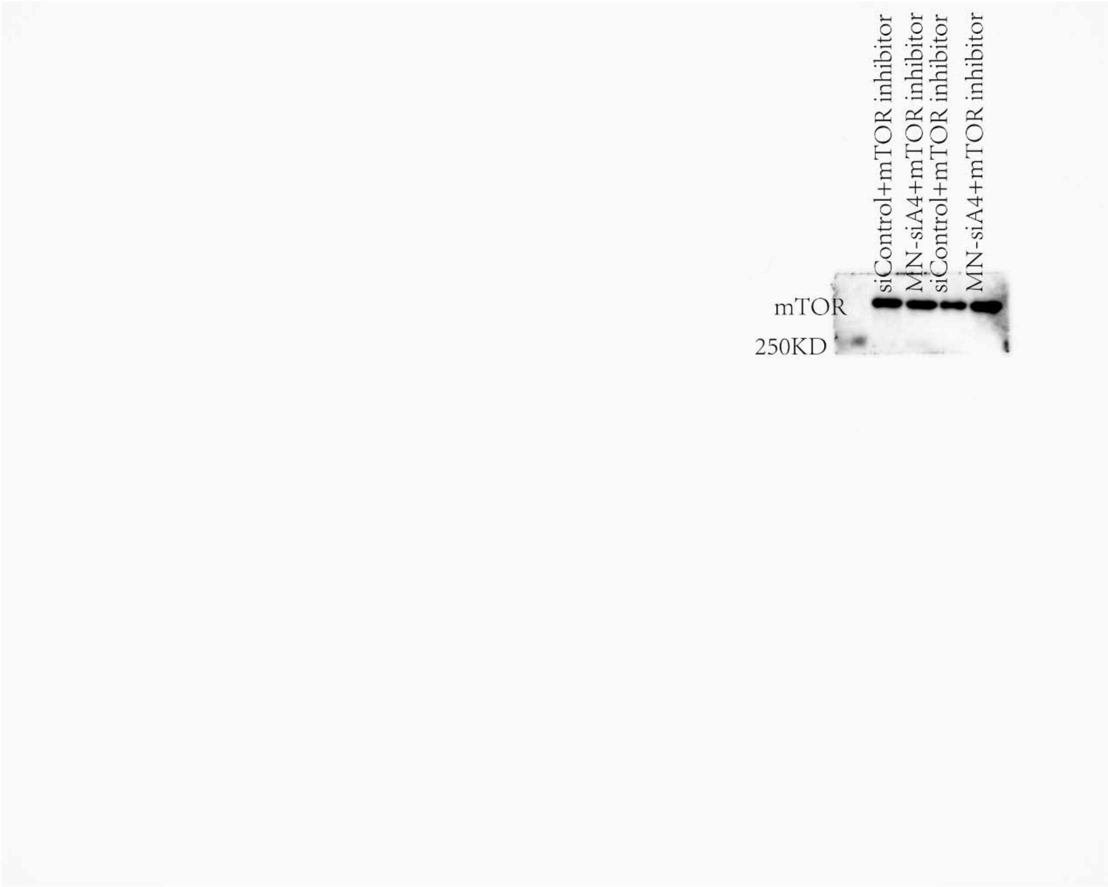

mTOR for Fig.6

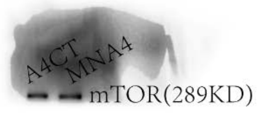

mTOR_A4

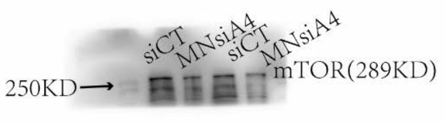

mTOR_sia4

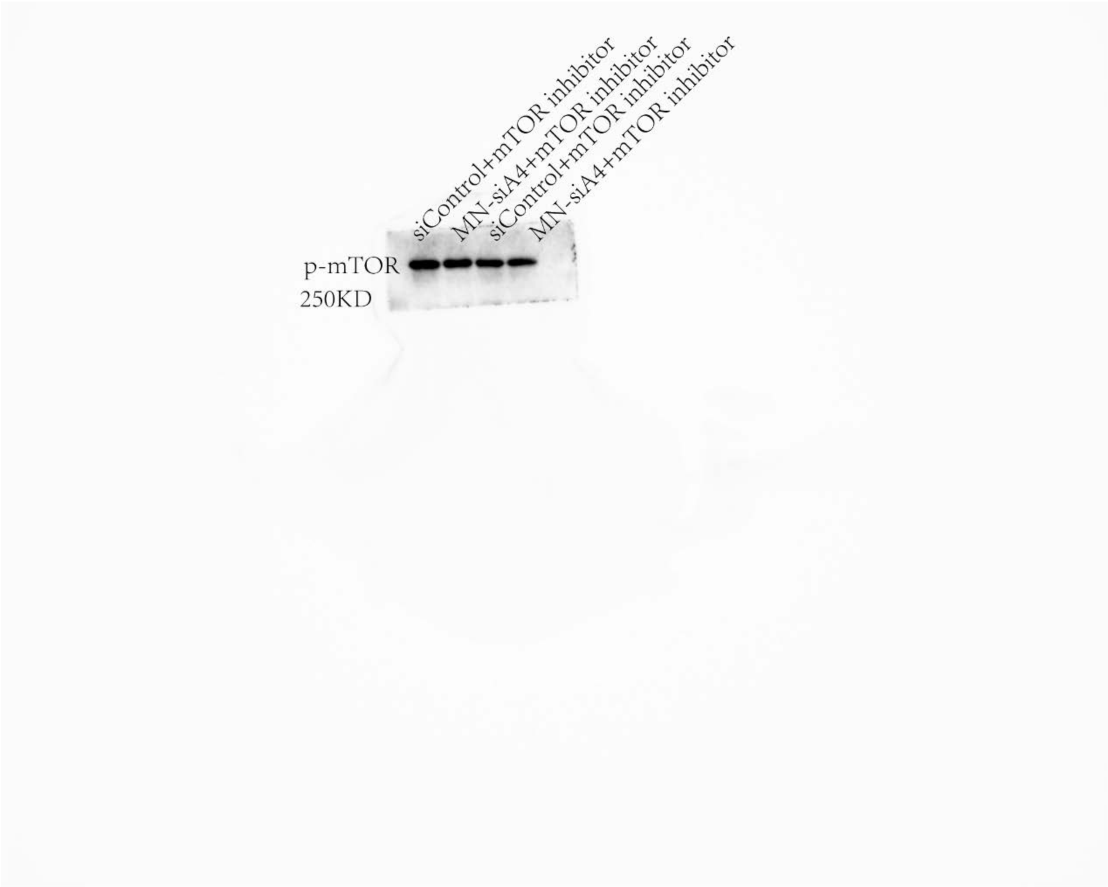

p-mTOR for Fig.6

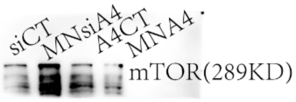

p-mTOR

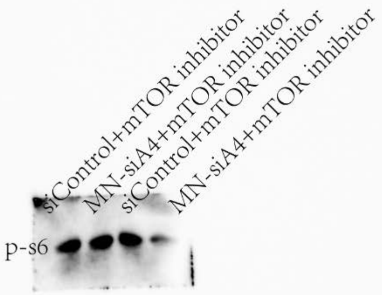
siControl+mTOR inhibitor
MN-siA4+mTOR inhibitor
siControl+mTOR inhibitor
MN-siA4+mTOR inhibitor
p-s6

p-s6 for Fig.6

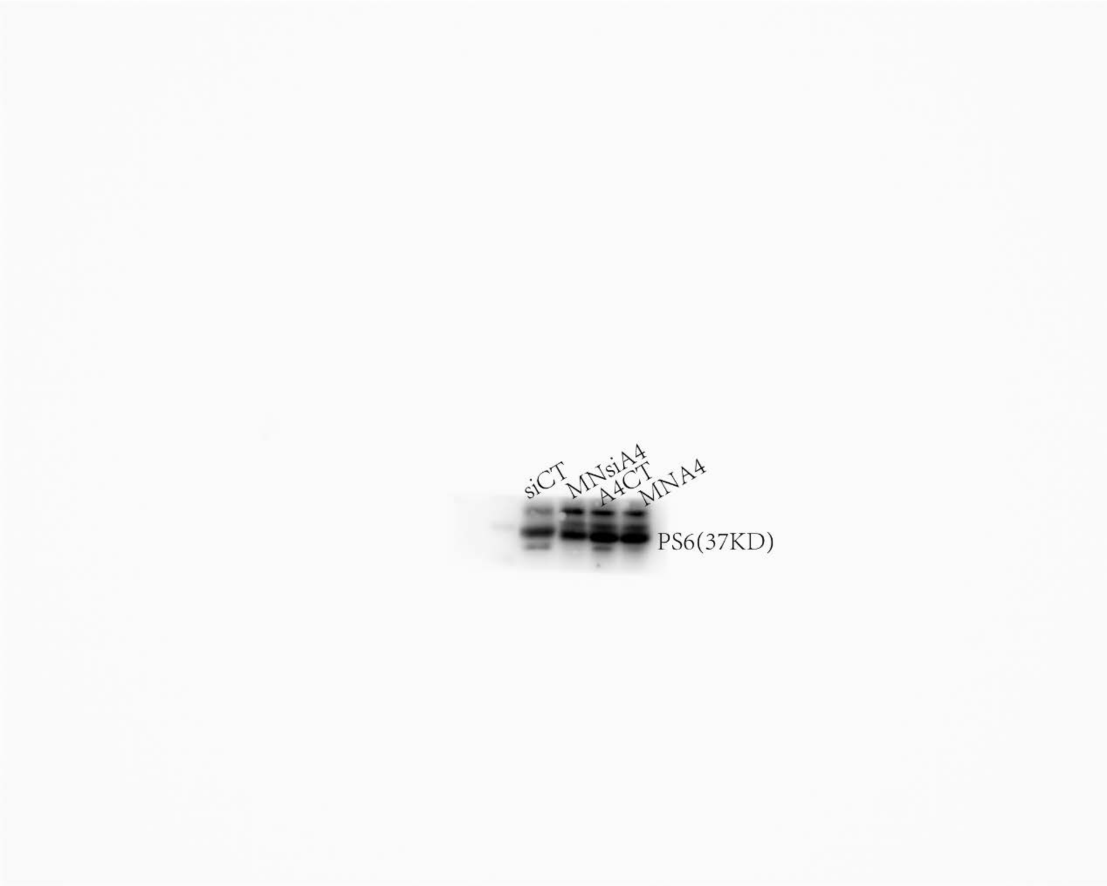

p-s6_a4 and sia4

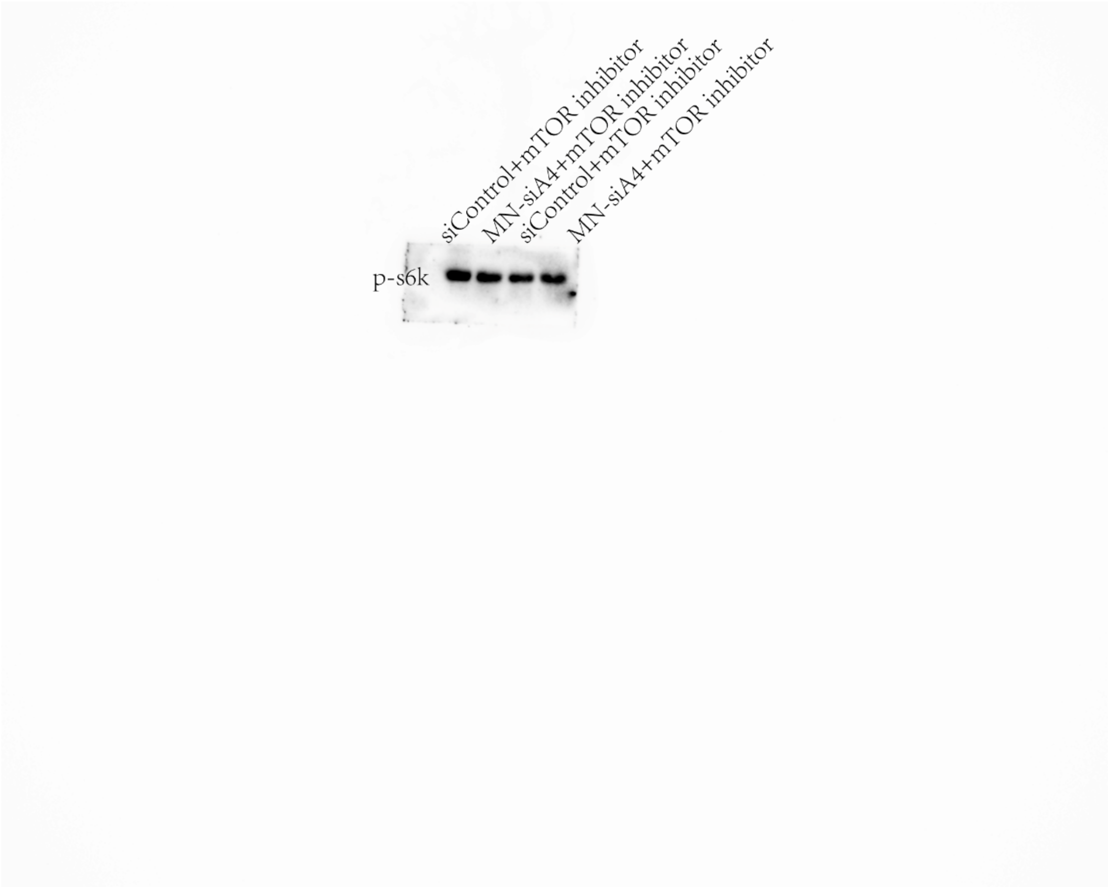

p-s6k for Fig.6

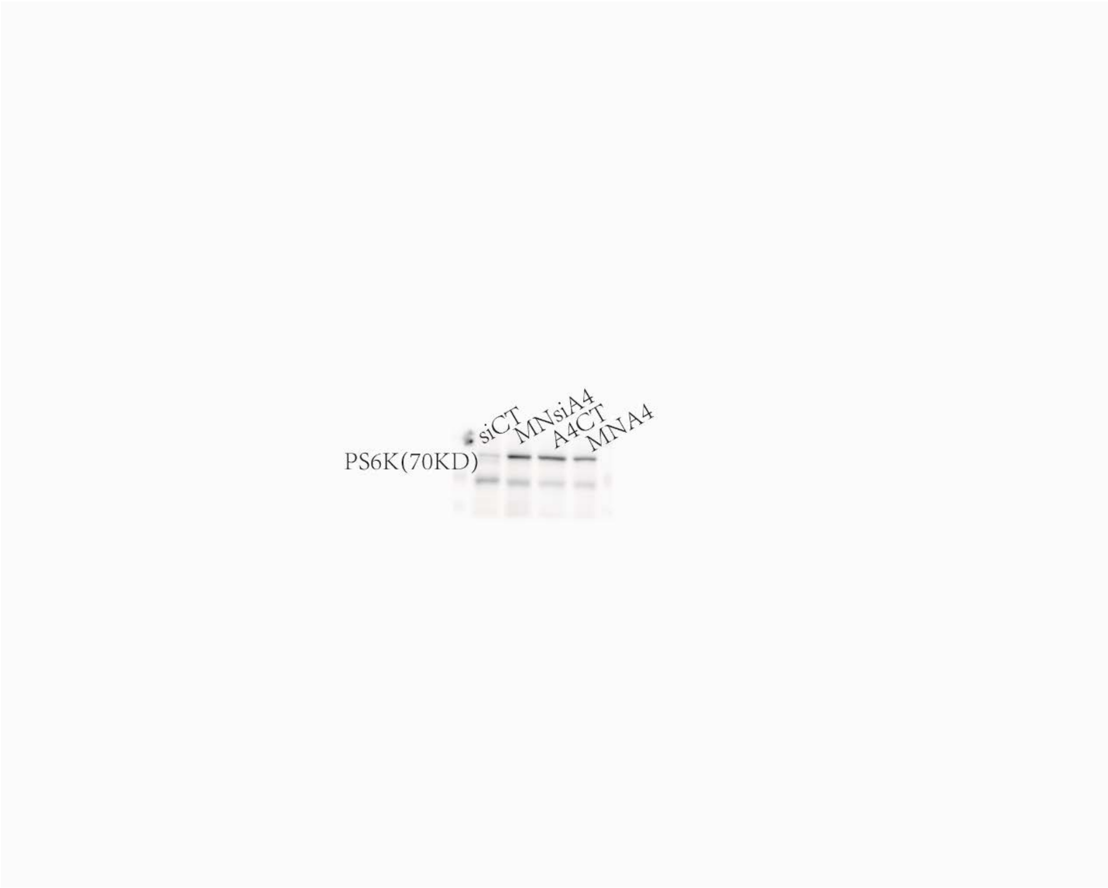

p-s6k_a4 and sia4

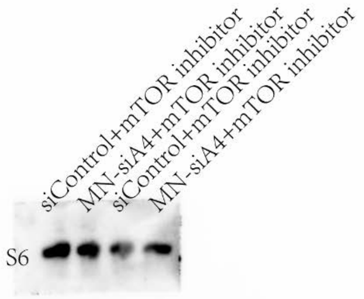
siControl+mTOR inhibitor
MN-siA4+mTOR inhibitor
siControl+mTOR inhibitor
MN-siA4+mTOR inhibitor
S6

s6 for Fig. 6

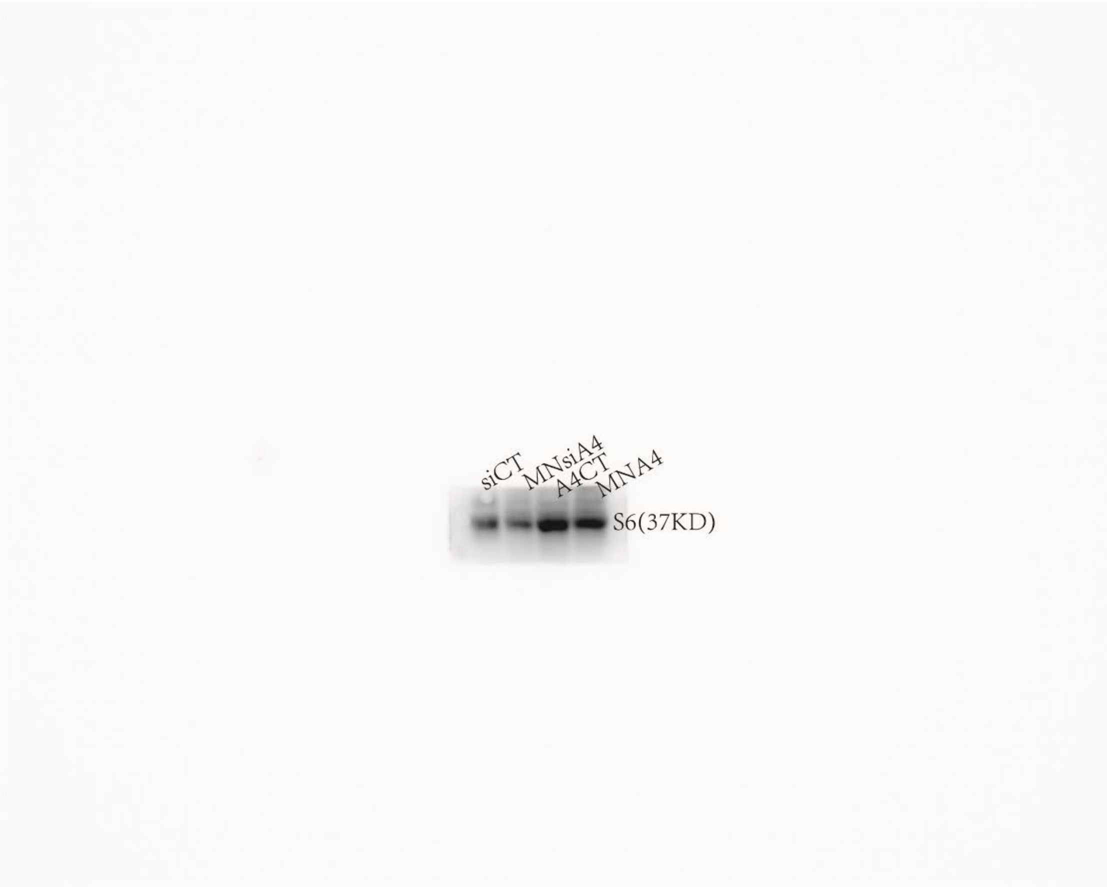

s6_a4 and sia4

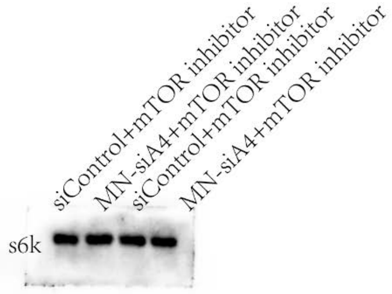
siControl+mTOR inhibitor
MN-siA4+mTOR inhibitor
siControl+mTOR inhibitor
MN-siA4+mTOR inhibitor
s6k

s6k for Fig.6

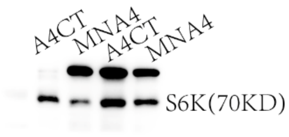

s6k_a4

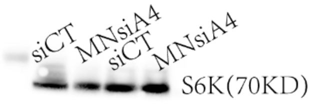

s6k_sia4

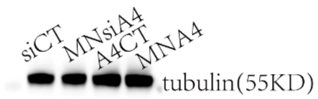

tubulin_Fig.4

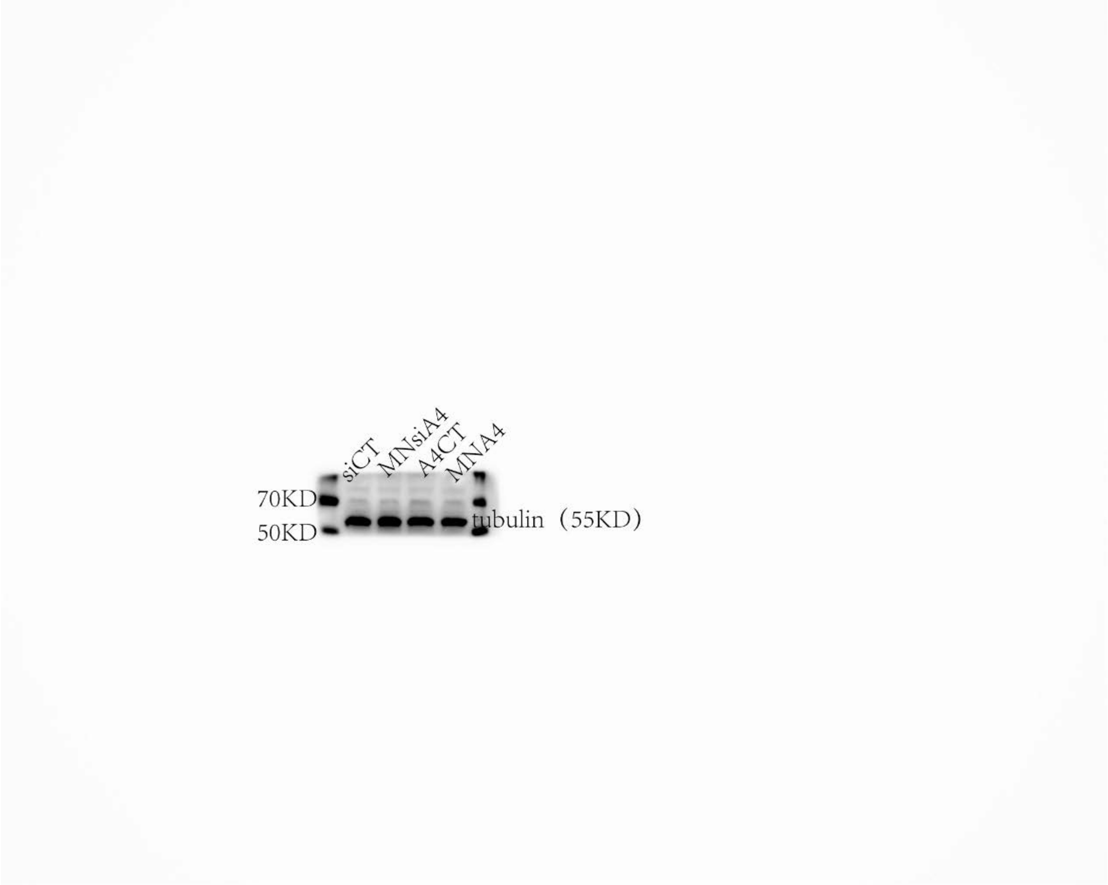

tubulin_fig4_1
