# Supplementary material for: ANGPTL4 negatively regulates the progression of osteosarcoma by remodeling branched-chain amino acid metabolism
Source: Cell Death Discov. 2022 Apr 23;8:225. doi: 10.1038/s41420-022-01029-x (PMC9035178; doi:10.1038/s41420-022-01029-x)
Supplement: Supplementary file 8 — Original Data File [file 41420_2022_1029_MOESM8_ESM.pdf]

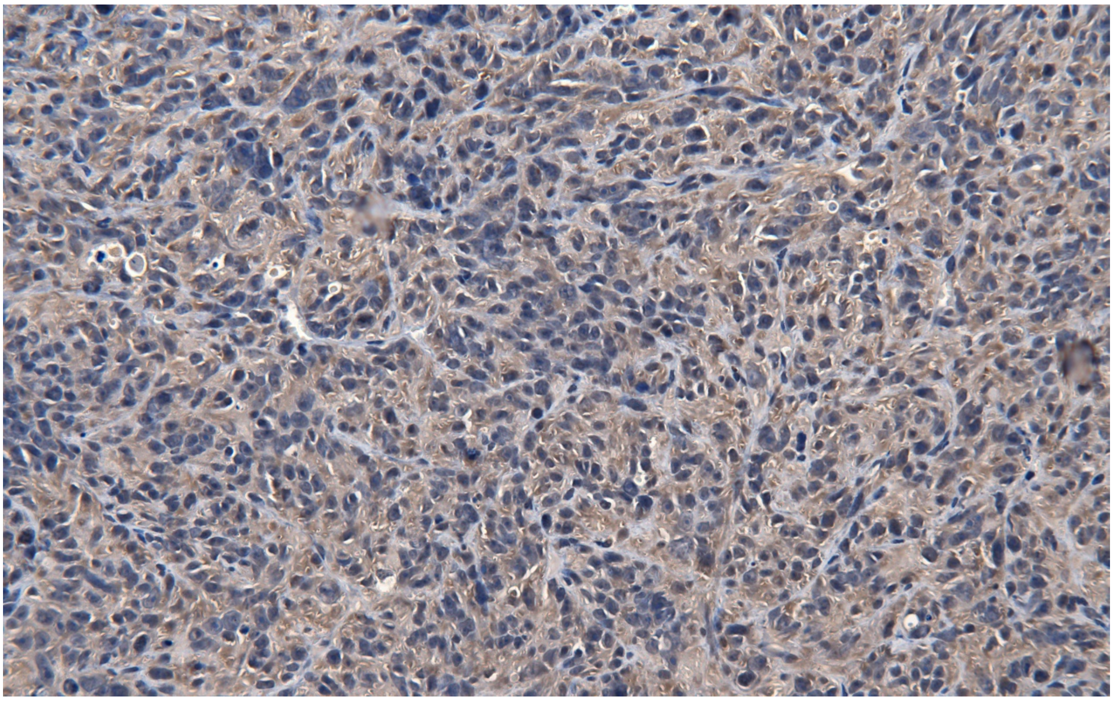

ANG4_A4Control_1

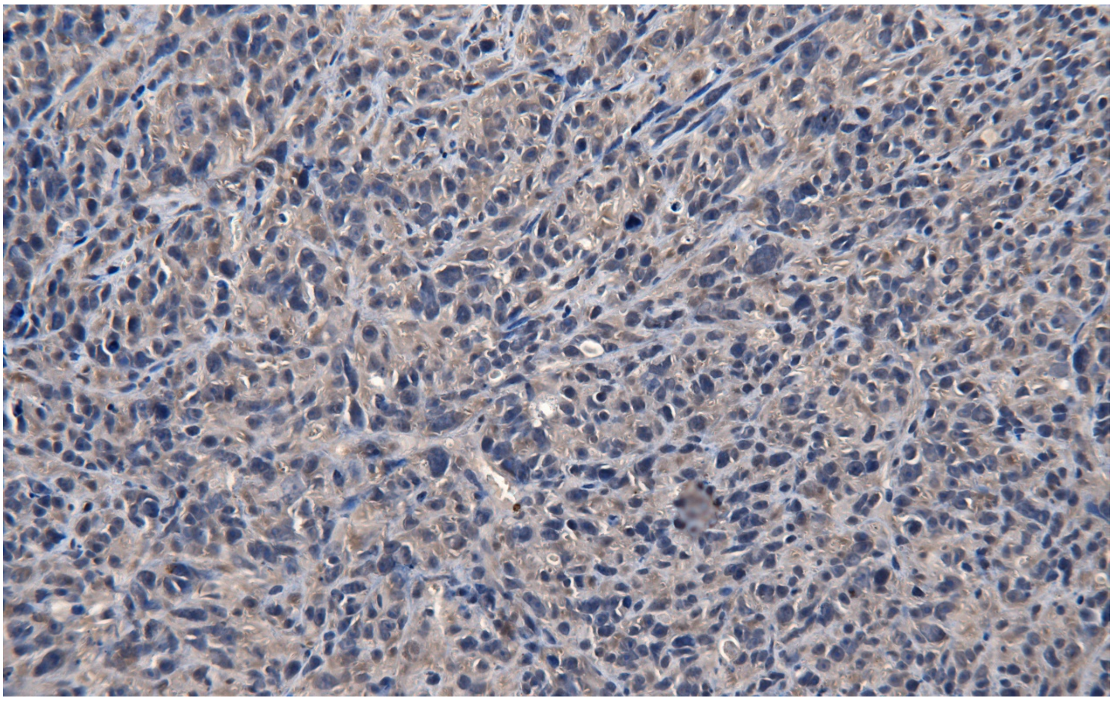

ANG4_A4Control_2

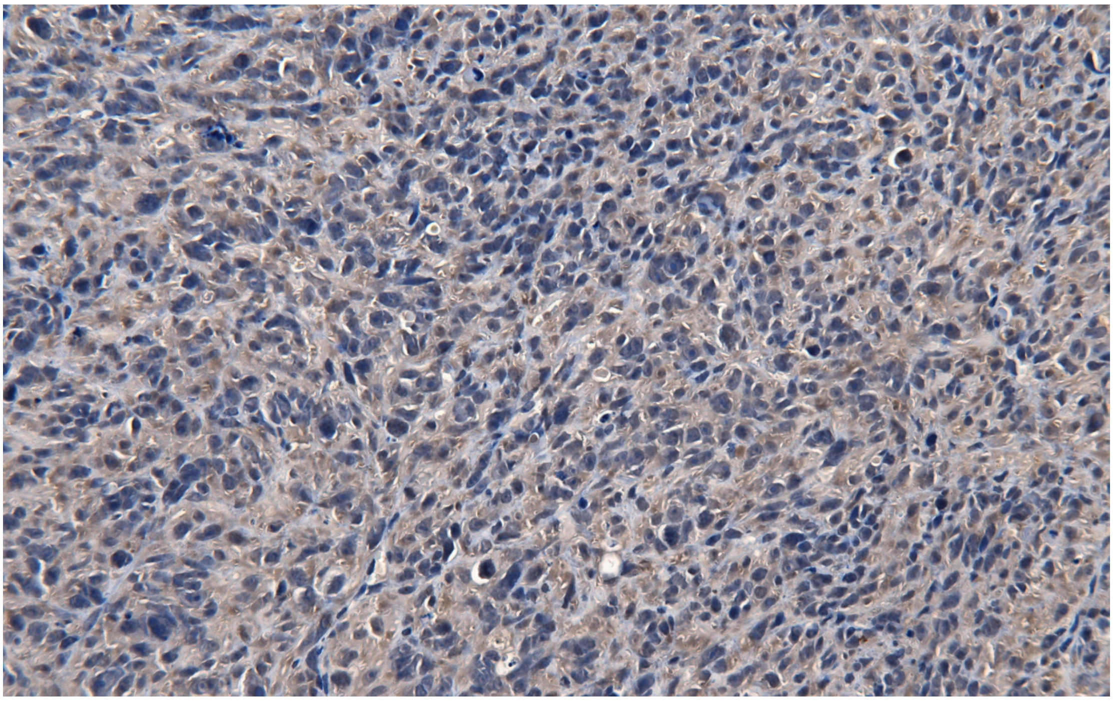

ANG4_A4Control_3

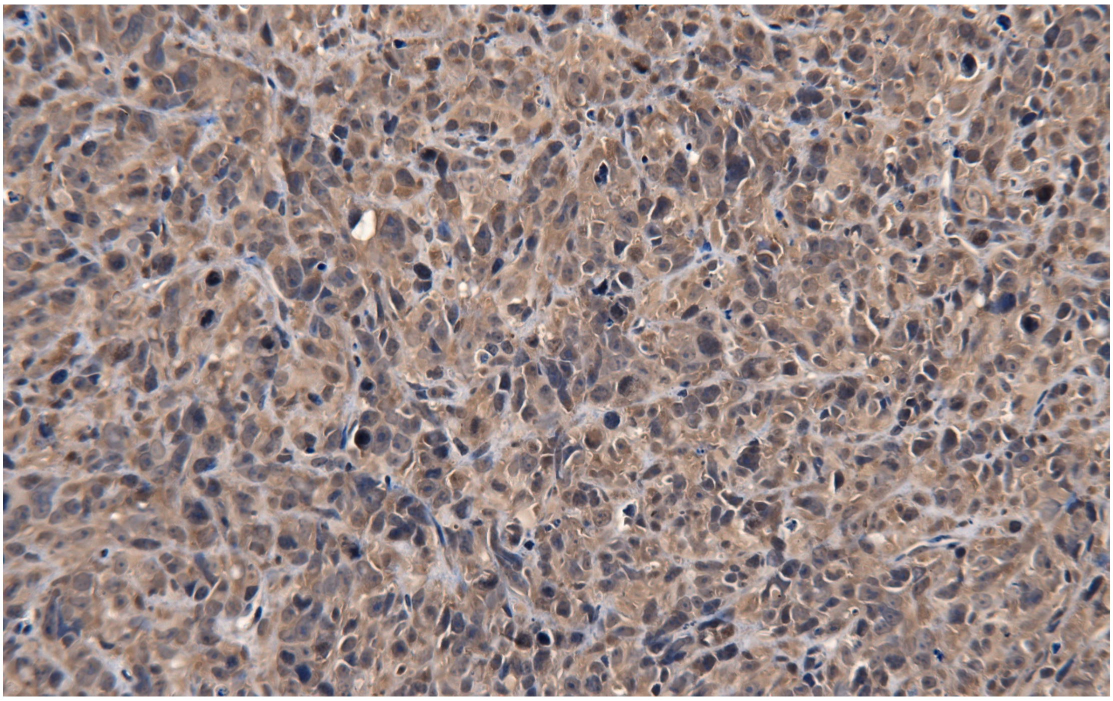

ANG4_MNA4_1

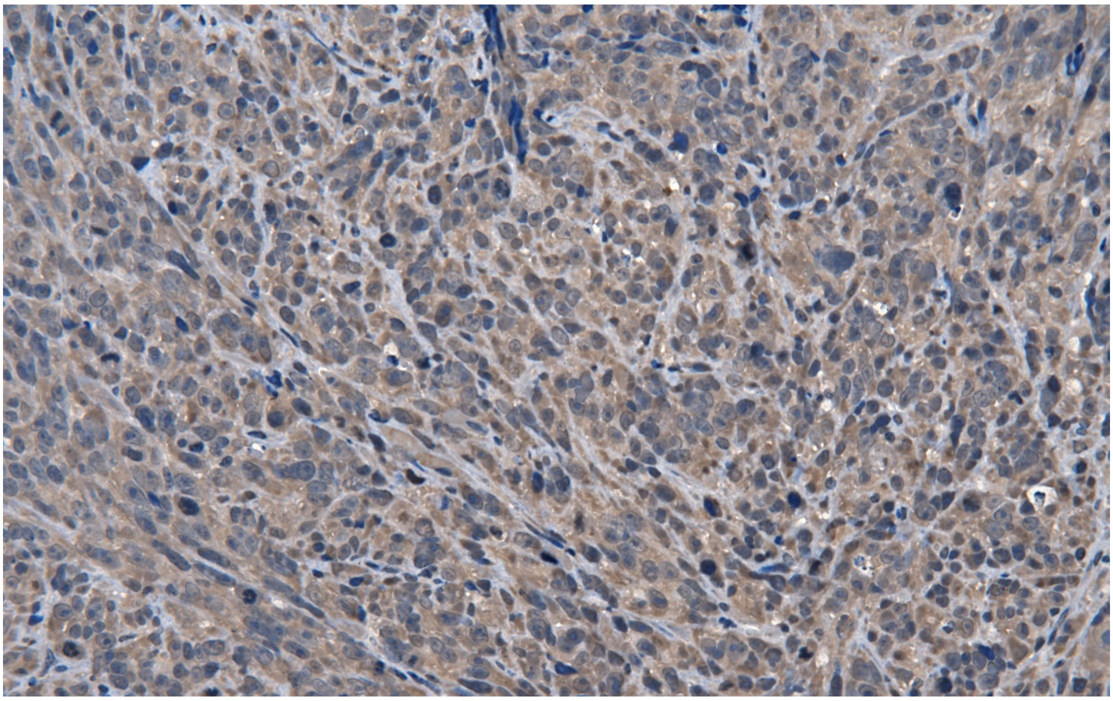

ANG4_MNA4_2

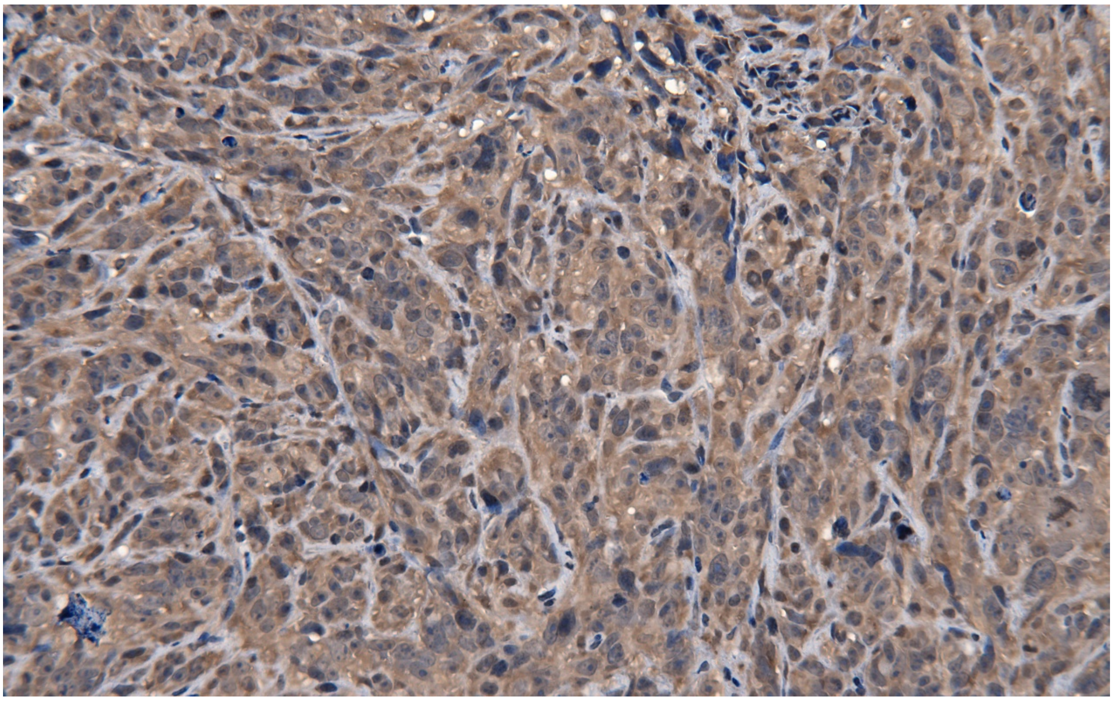

ANG4_MNA4_3

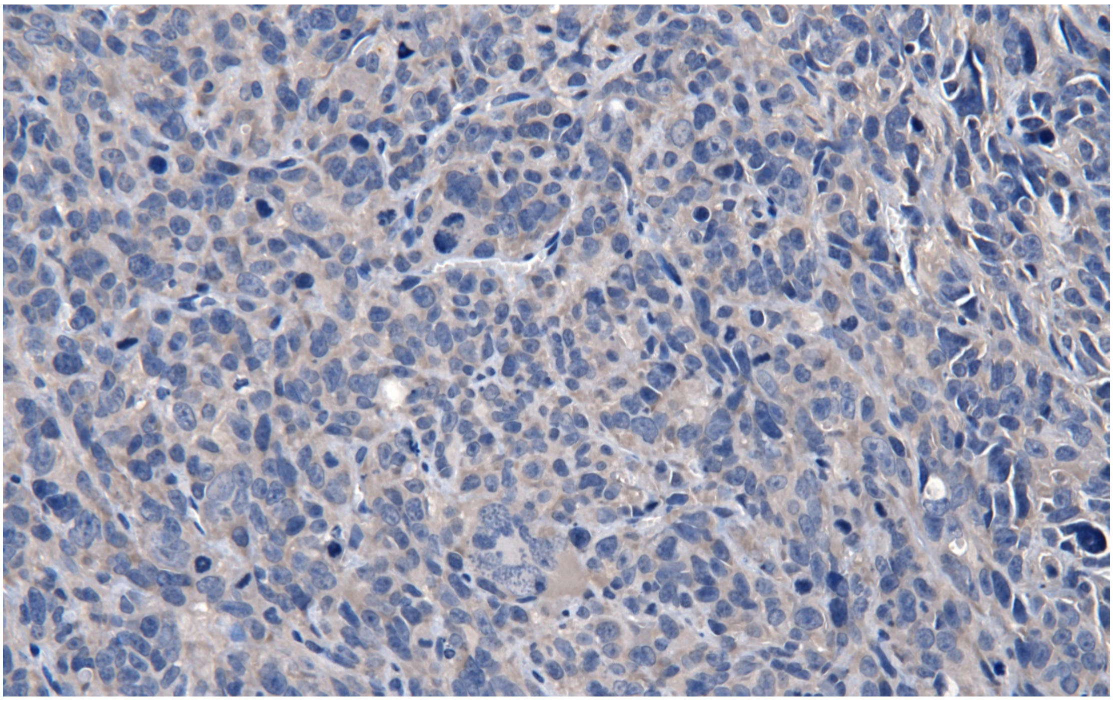

ANG4_MNsiA4_1

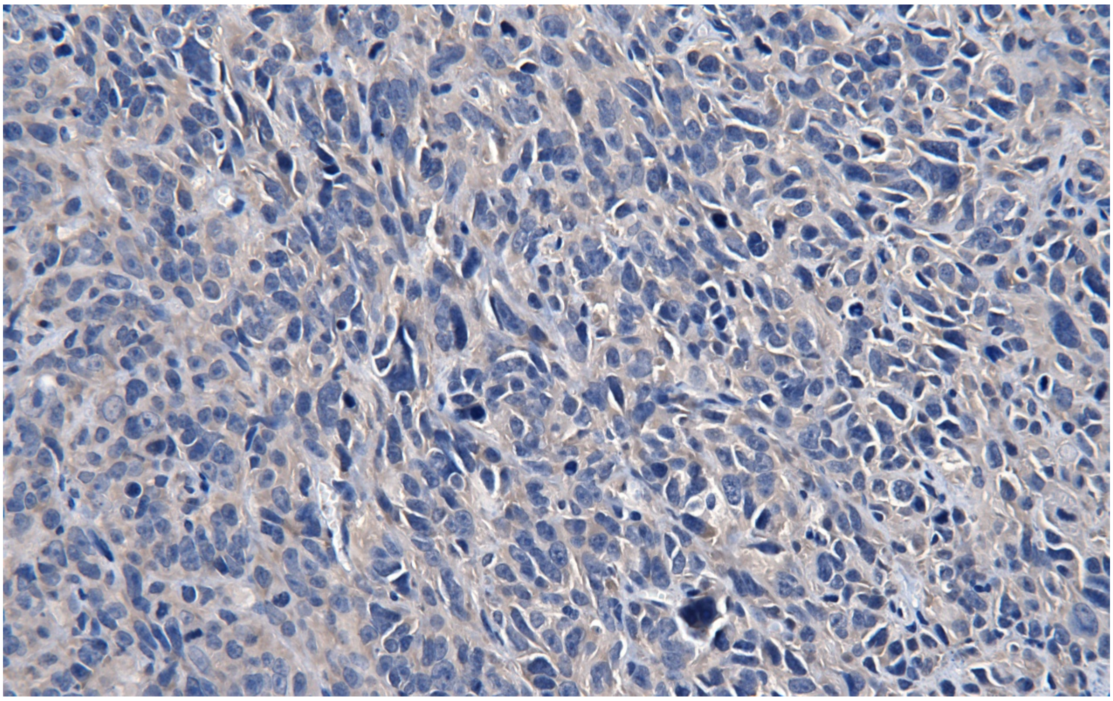

ANG4_MNsiA4_2

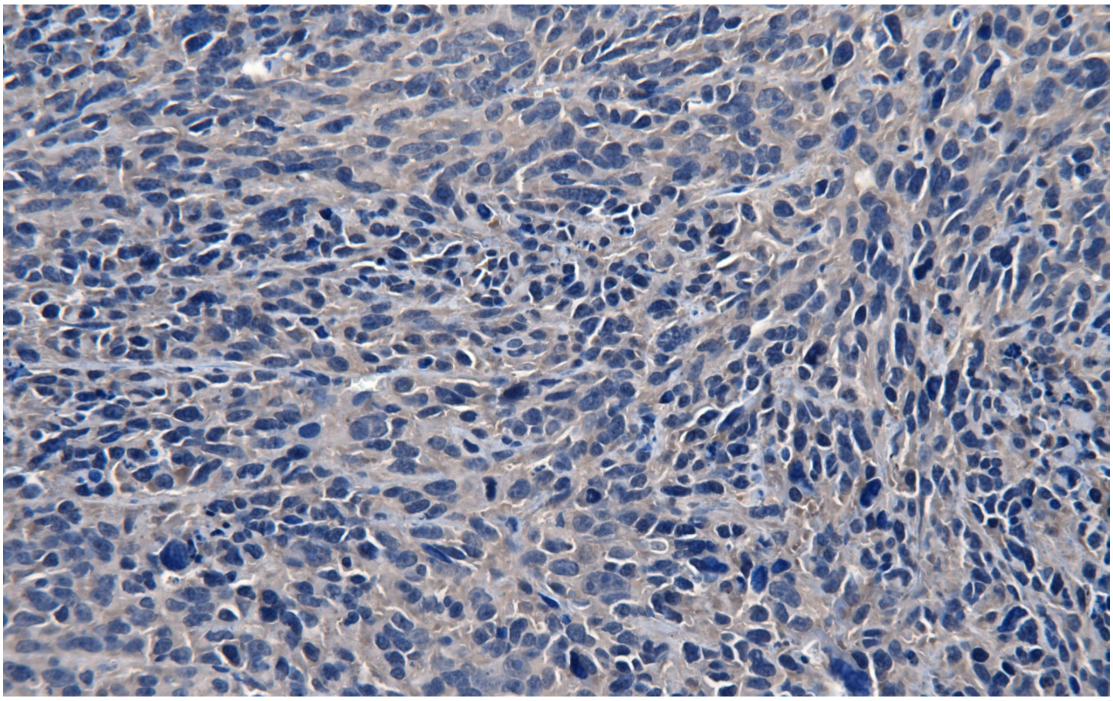

ANG4_MNsiA4_3

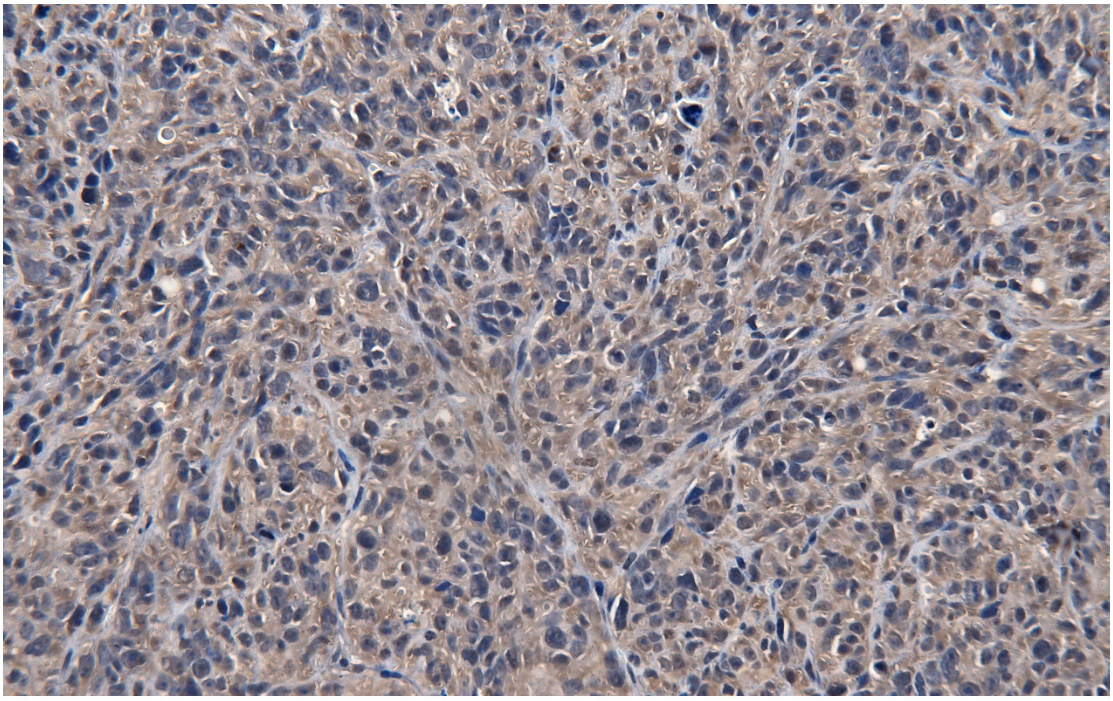

ANG4_siControl_1

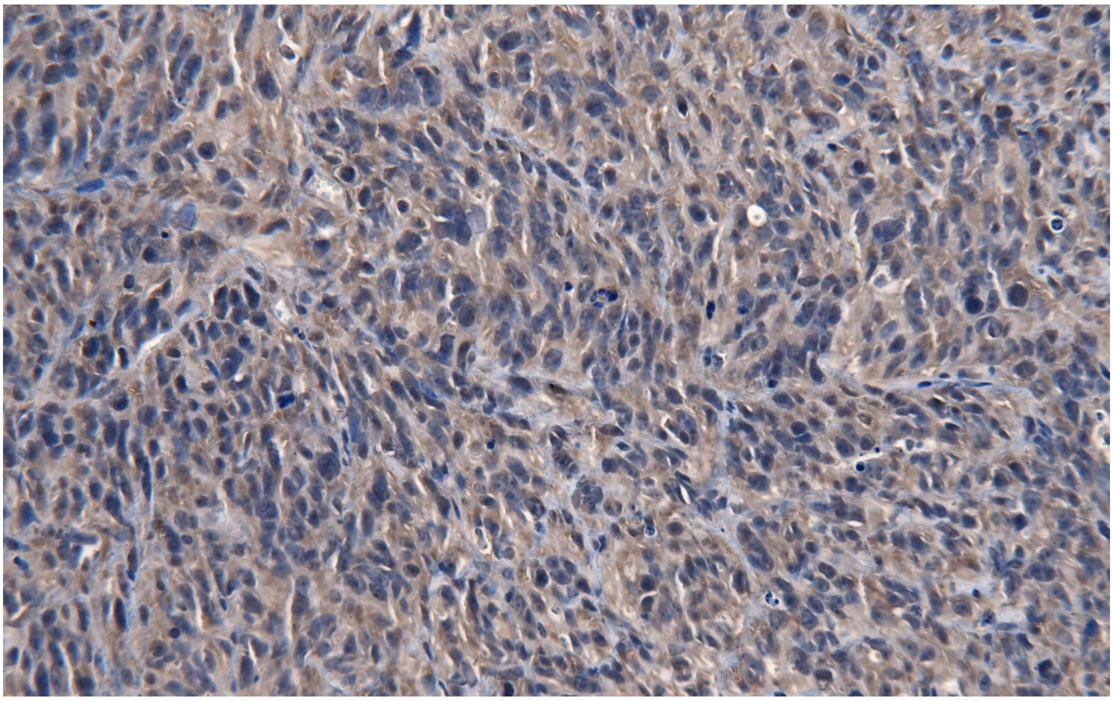

ANG4_siControl_2

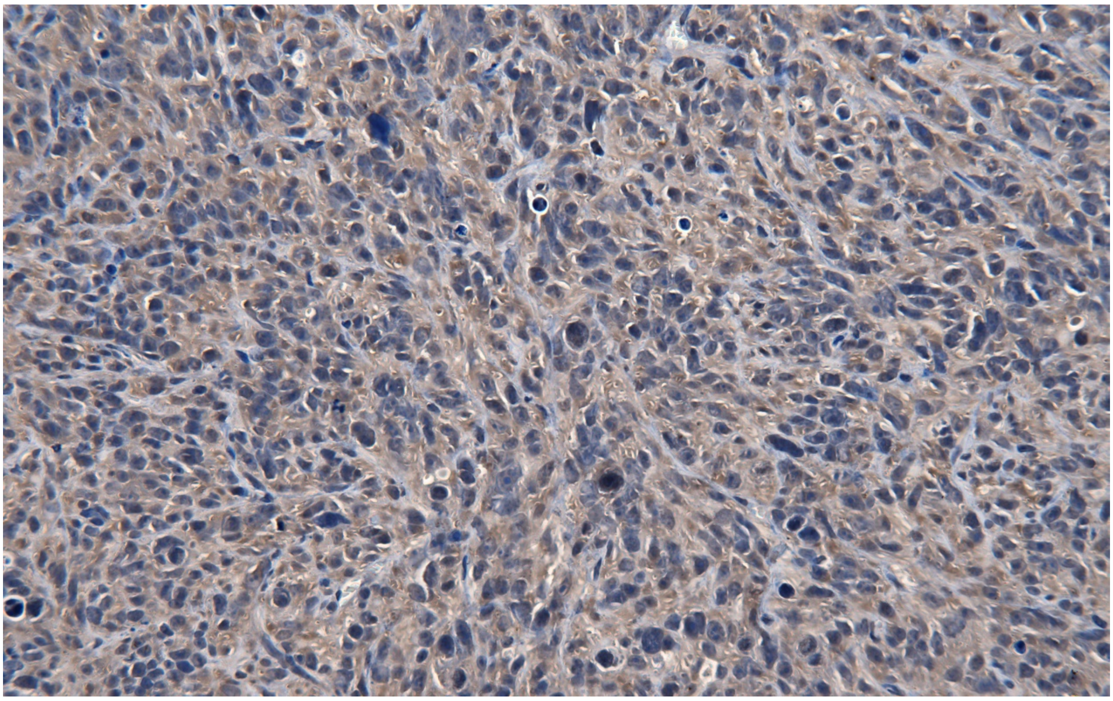

ANG4_siControl_3

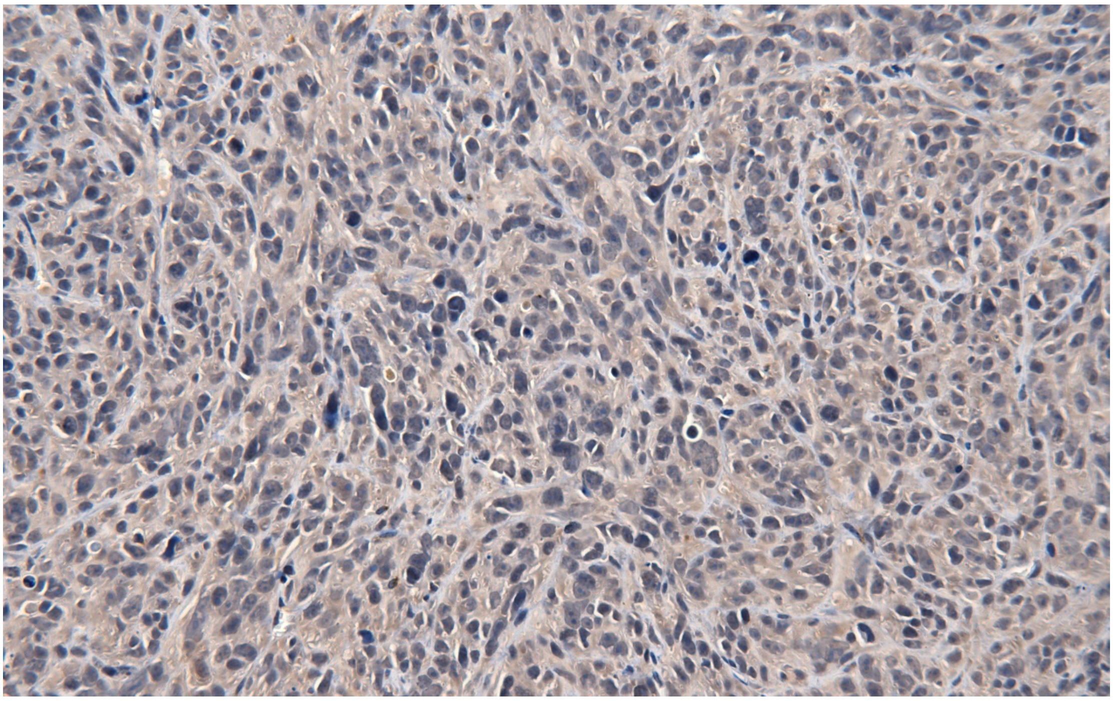

BCAT1_A4Control_1

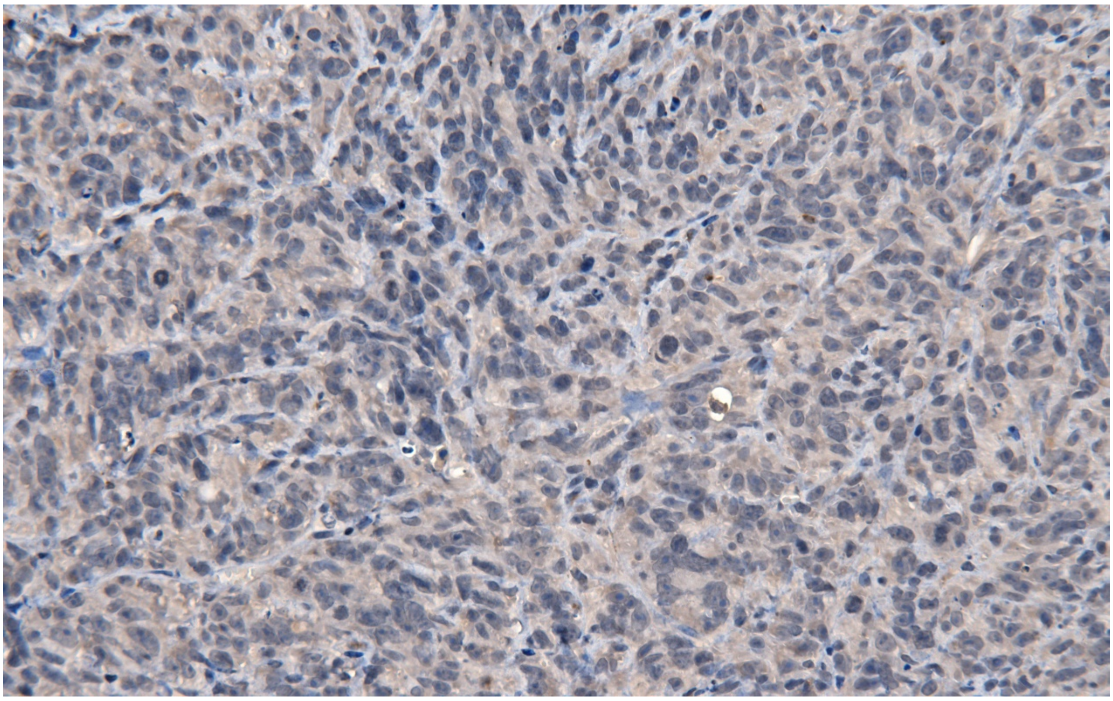

BCAT1_A4Control_2

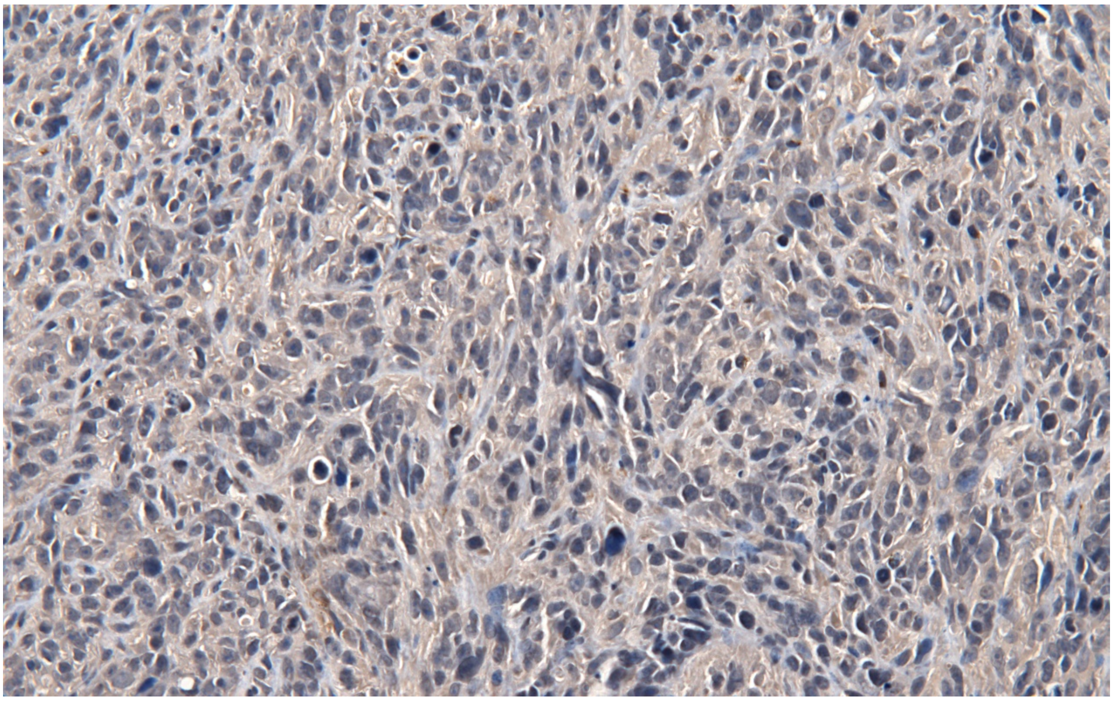

BCAT1_A4Control_3

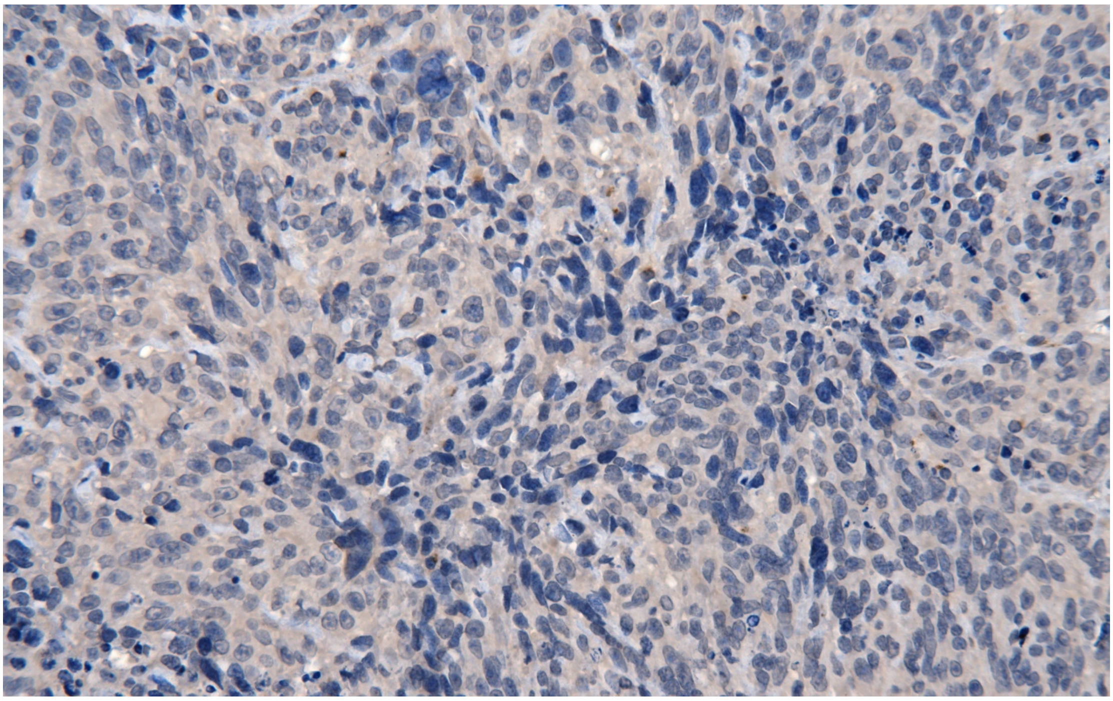

BCAT1_MNA4_1

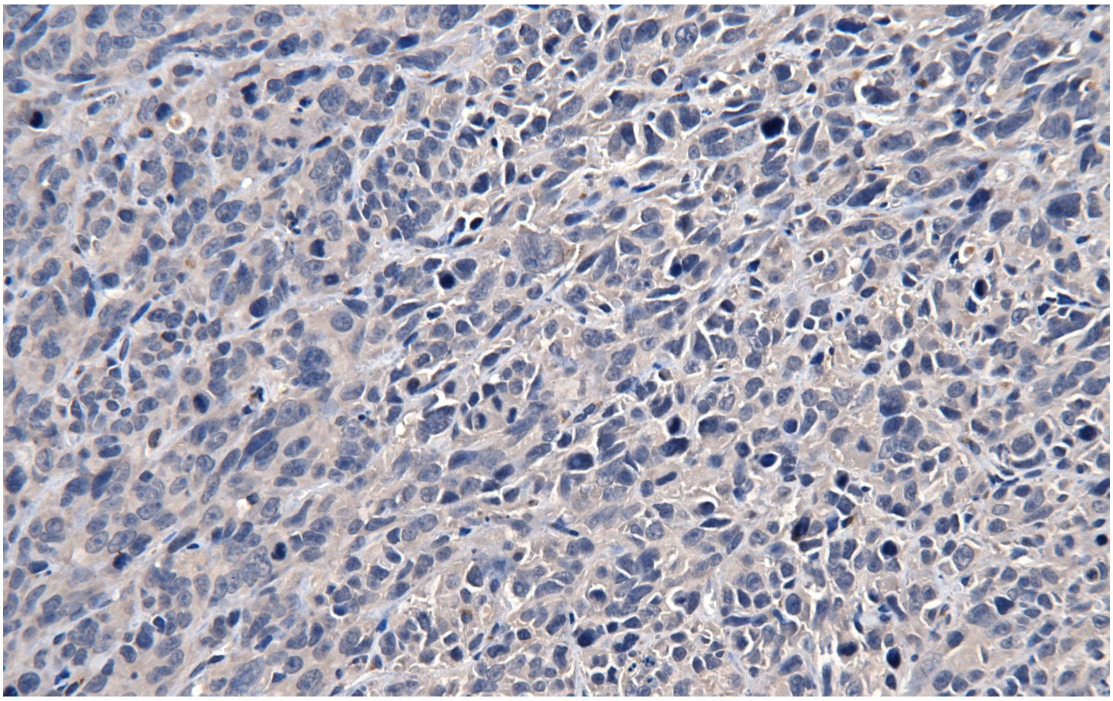

BCAT1_MNA4_2

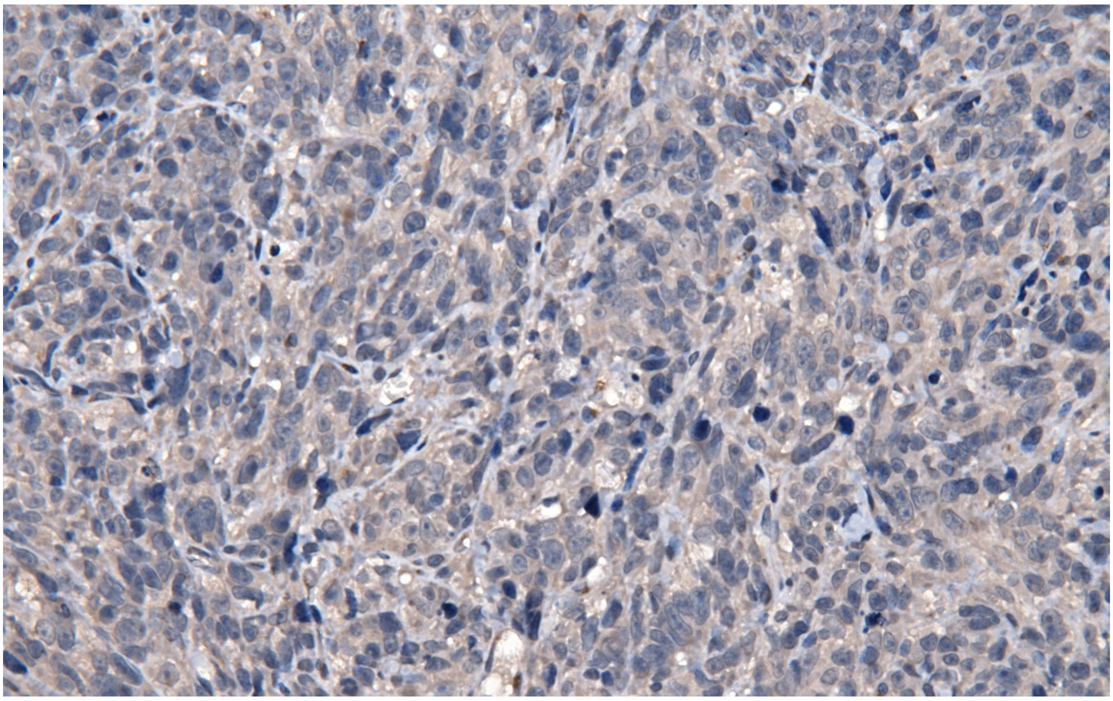

BCAT1_MNA4_3

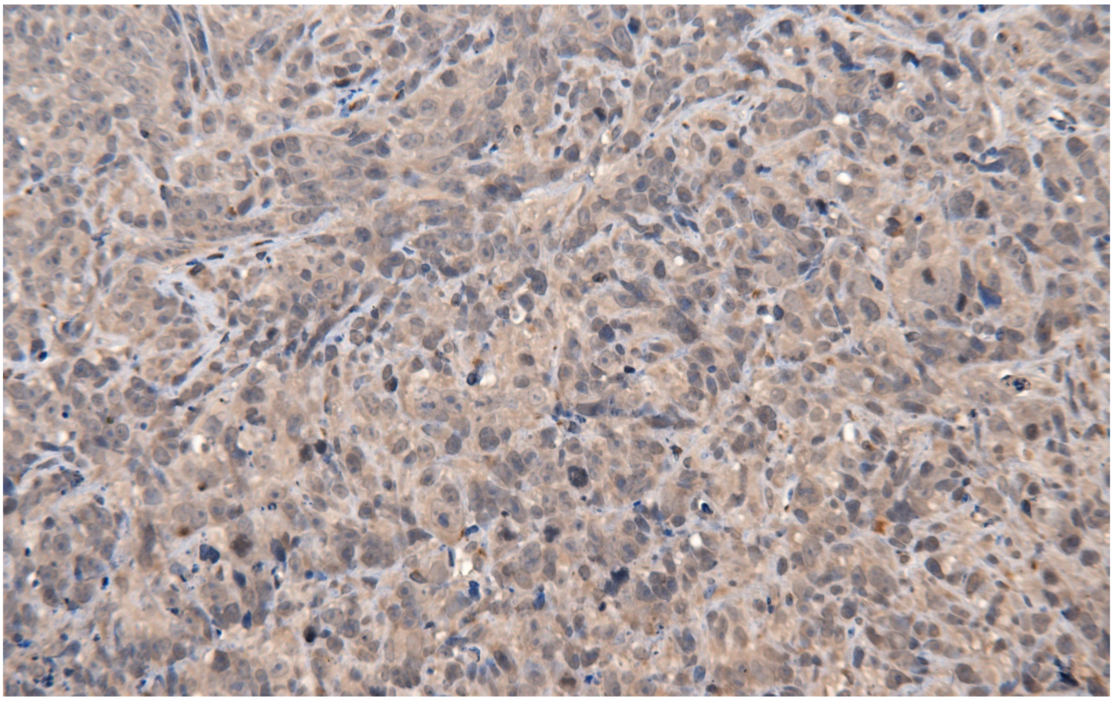

BCAT1_MNsiA4_1

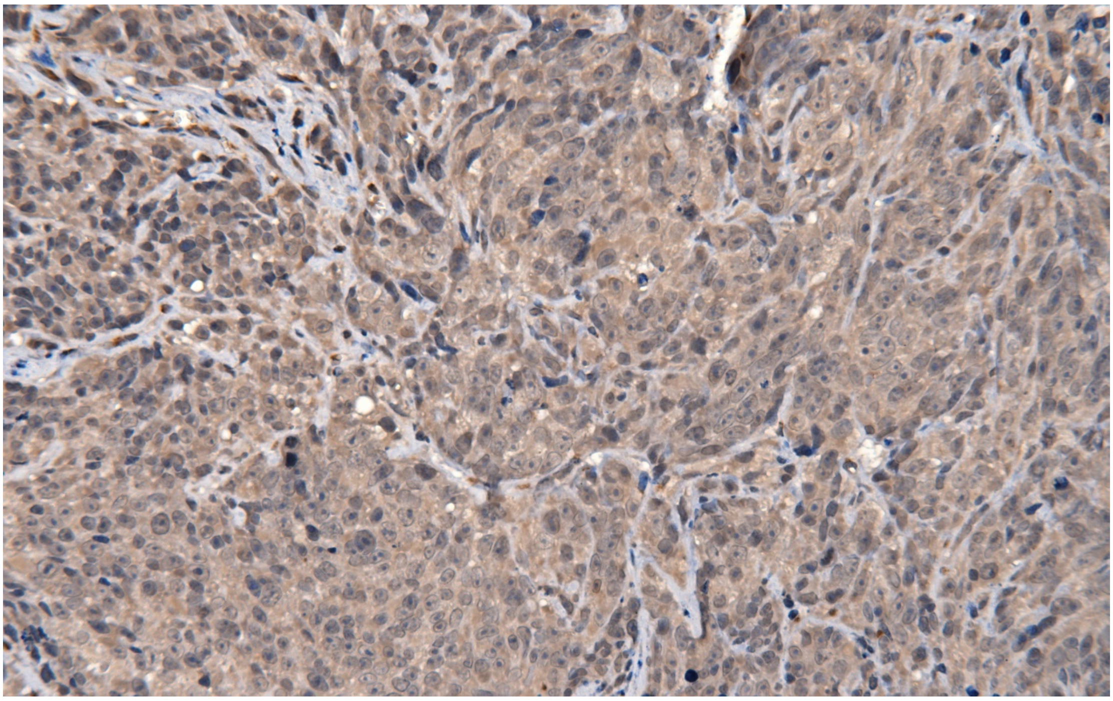

BCAT1_MNsiA4_2

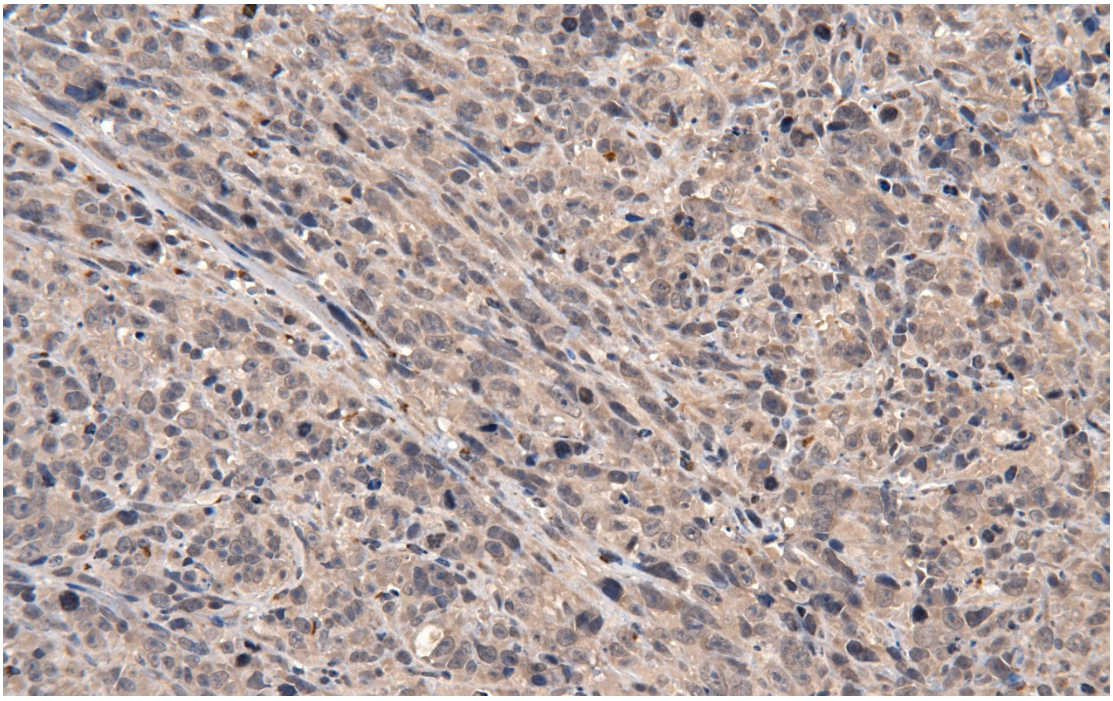

BCAT1_MNsiA4_3

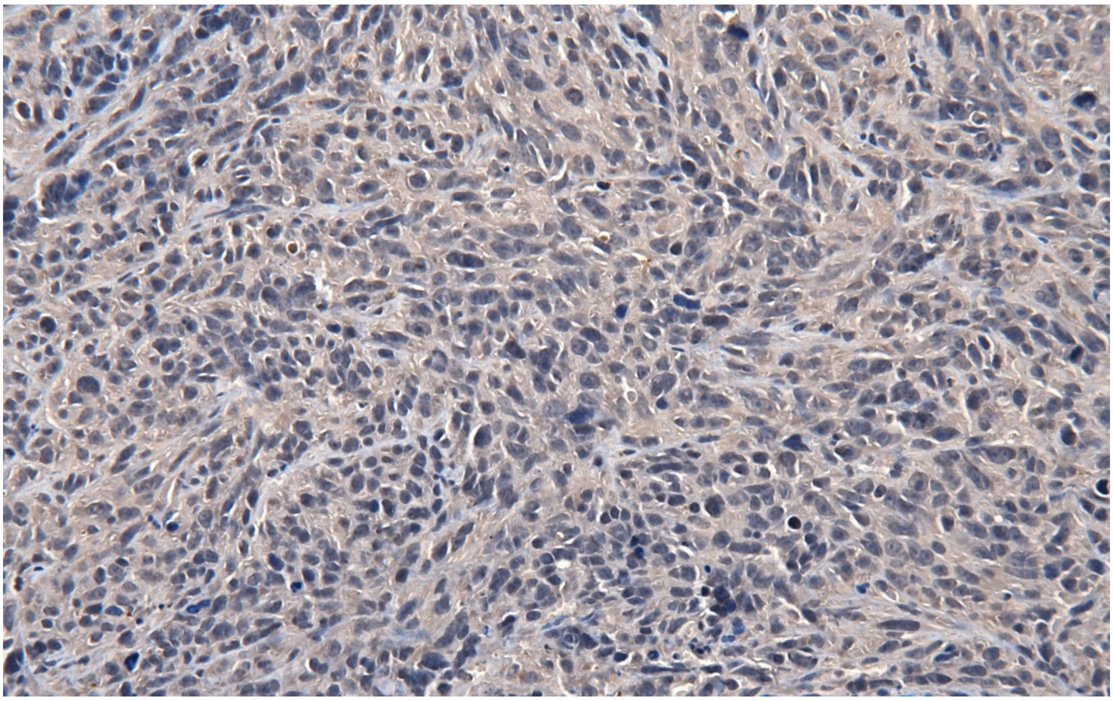

BCAT1_siControl_1

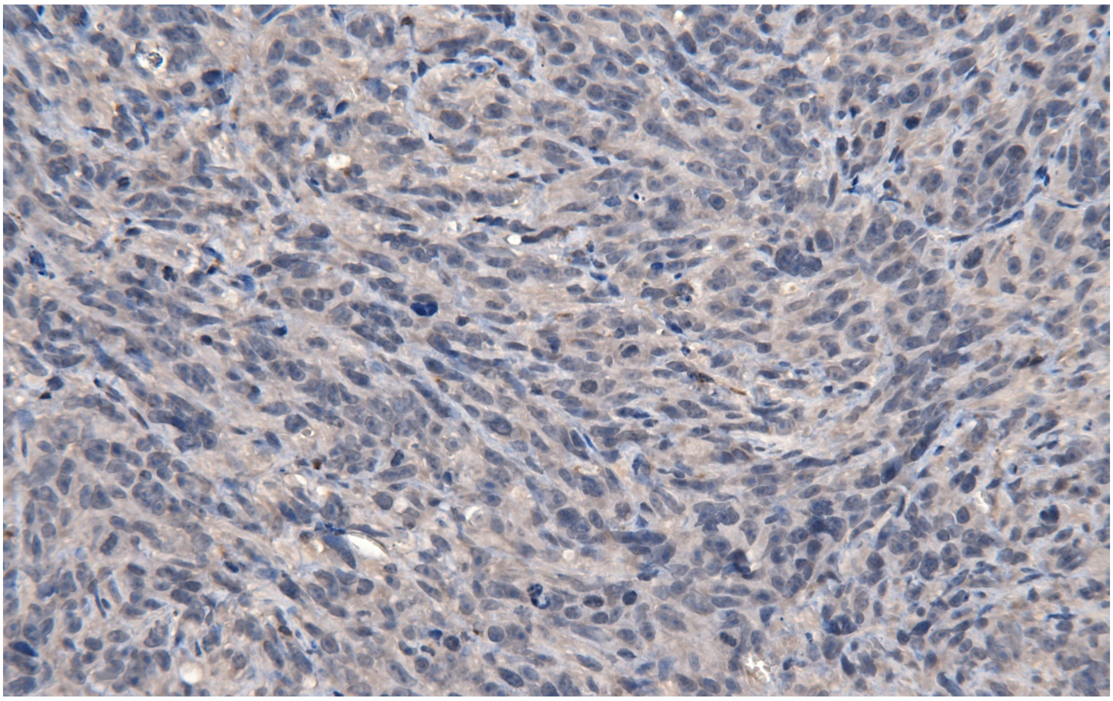

BCAT1_siControl_2

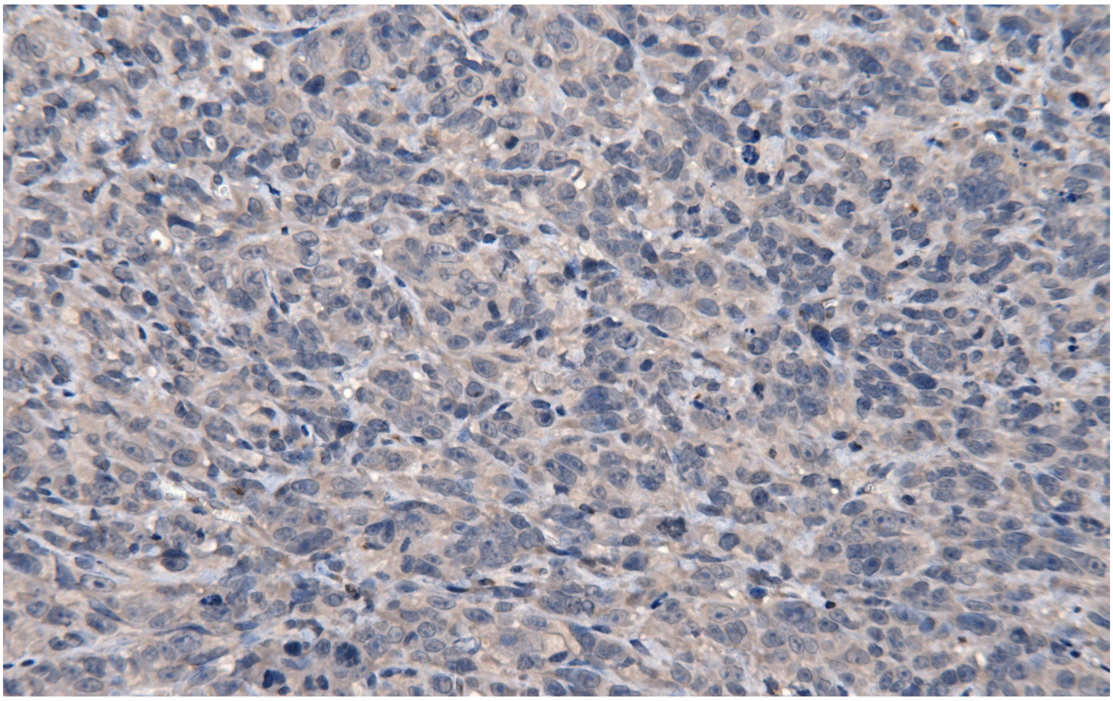

BCAT1_siControl_3

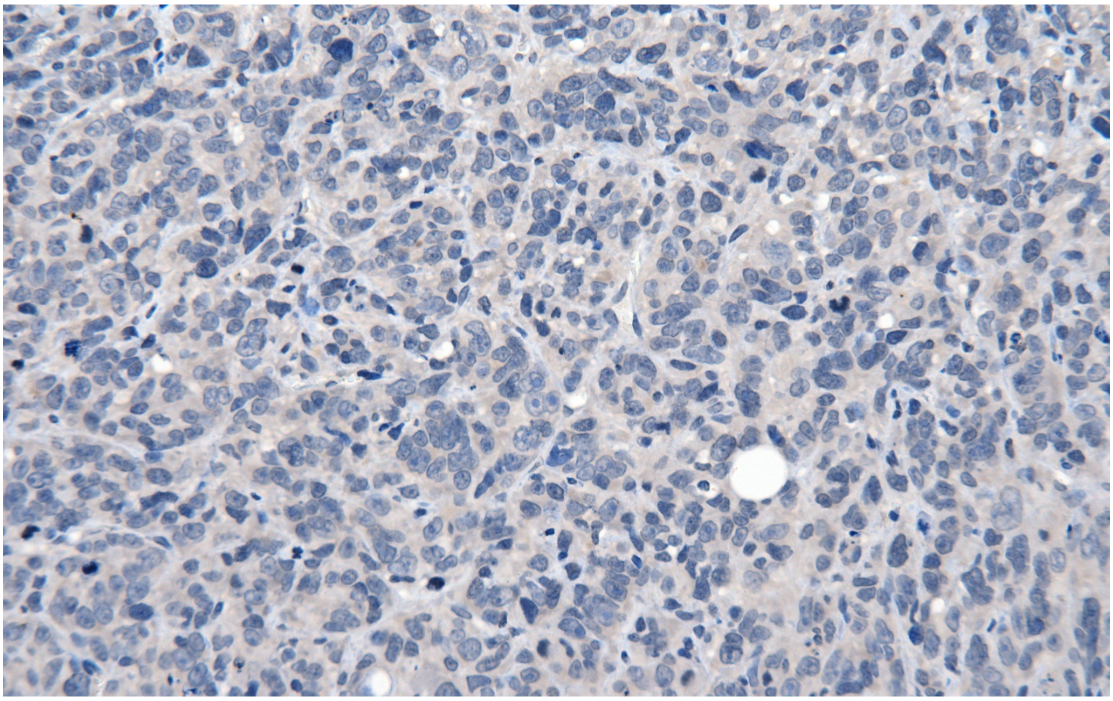

BCKDHA_A4Control_1

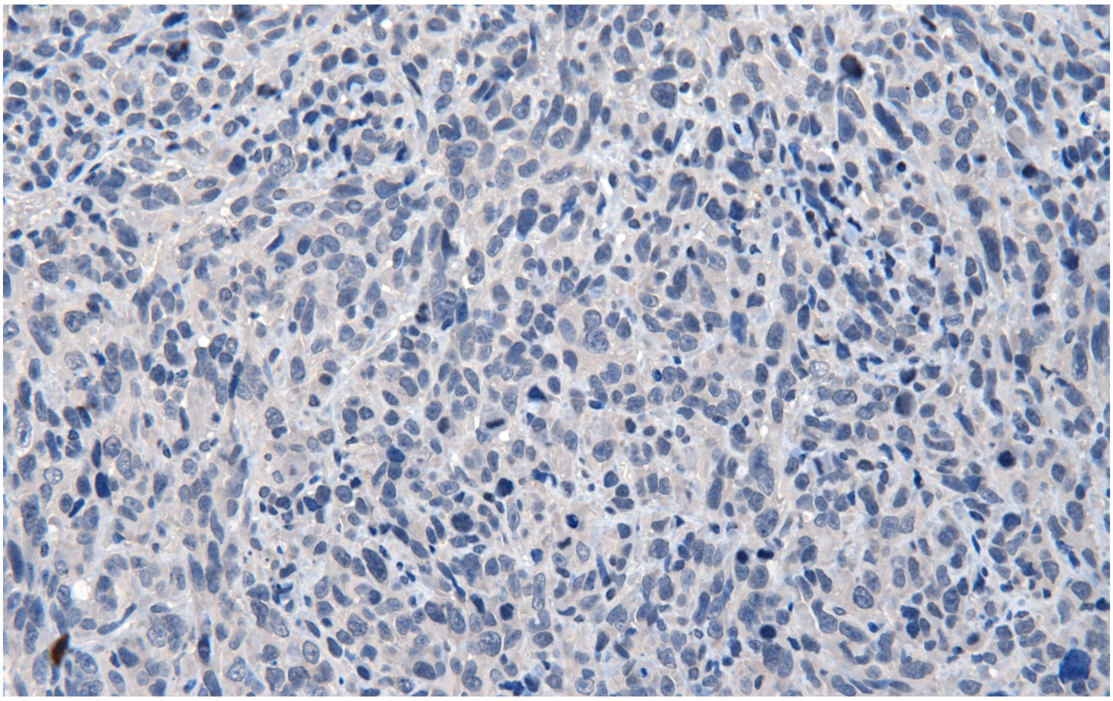

BCKDHA_A4Control_2

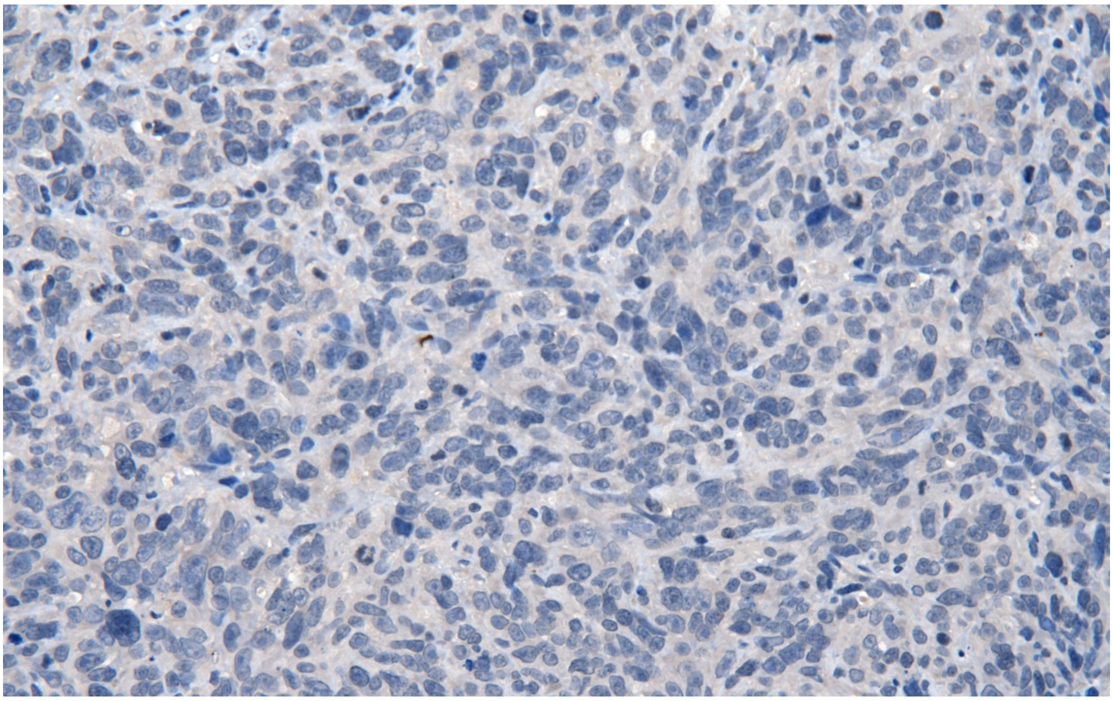

BCKDHA_A4Control_3

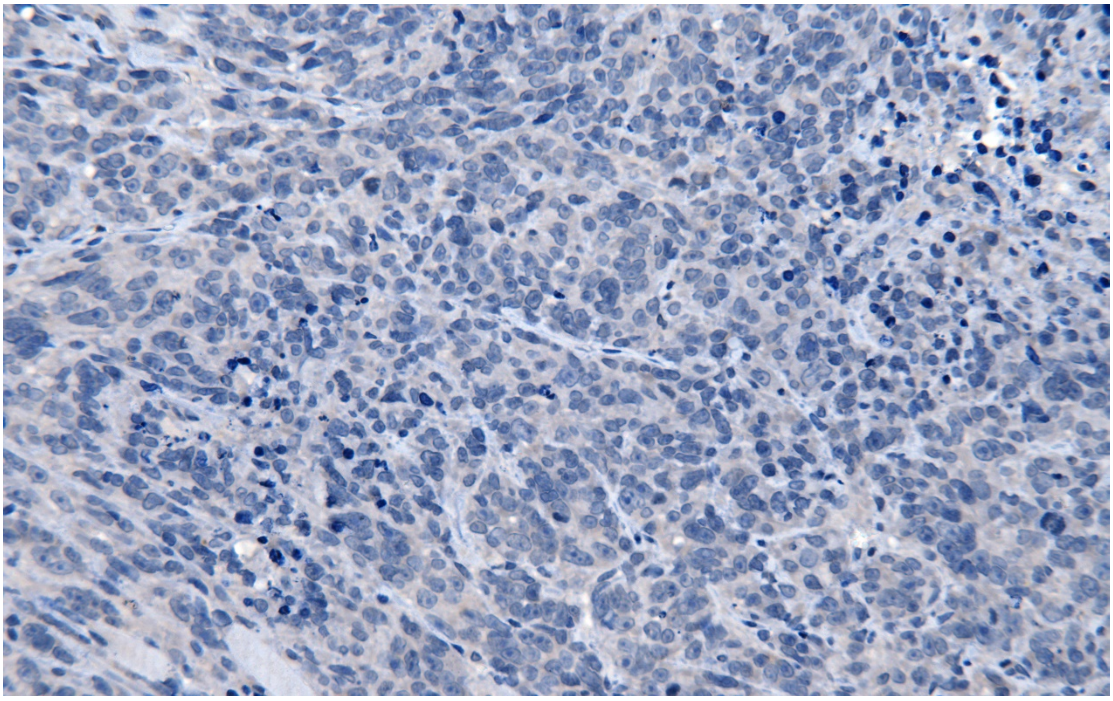

BCKDHA_MNA4_1

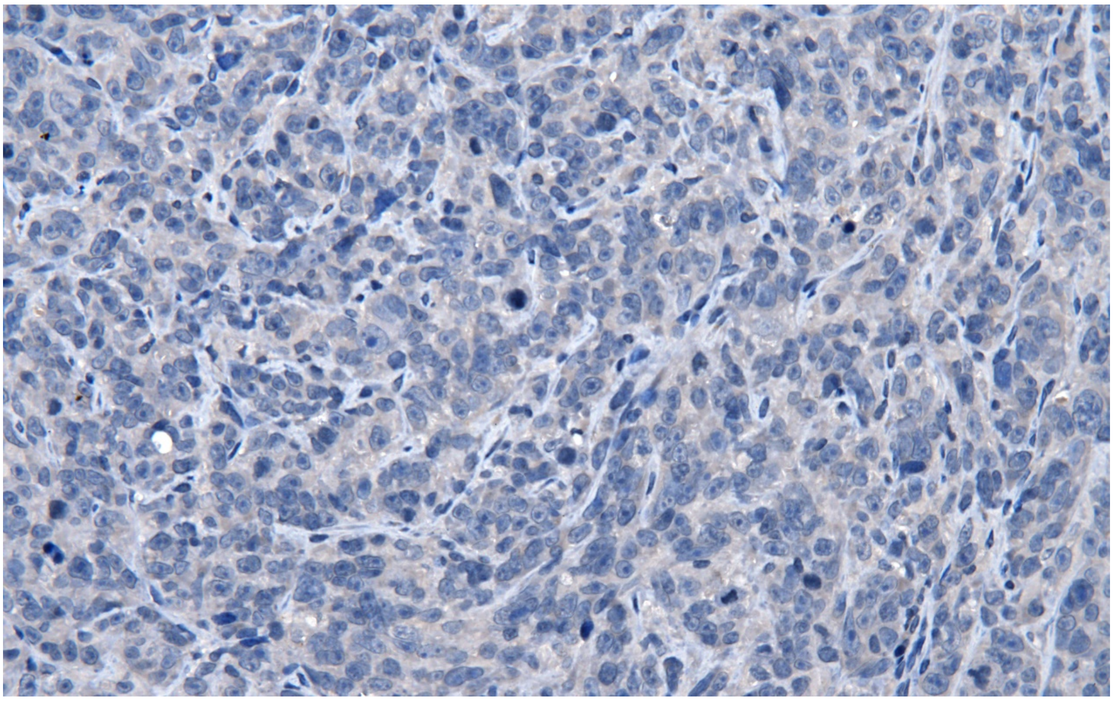

BCKDHA_MNA4_2

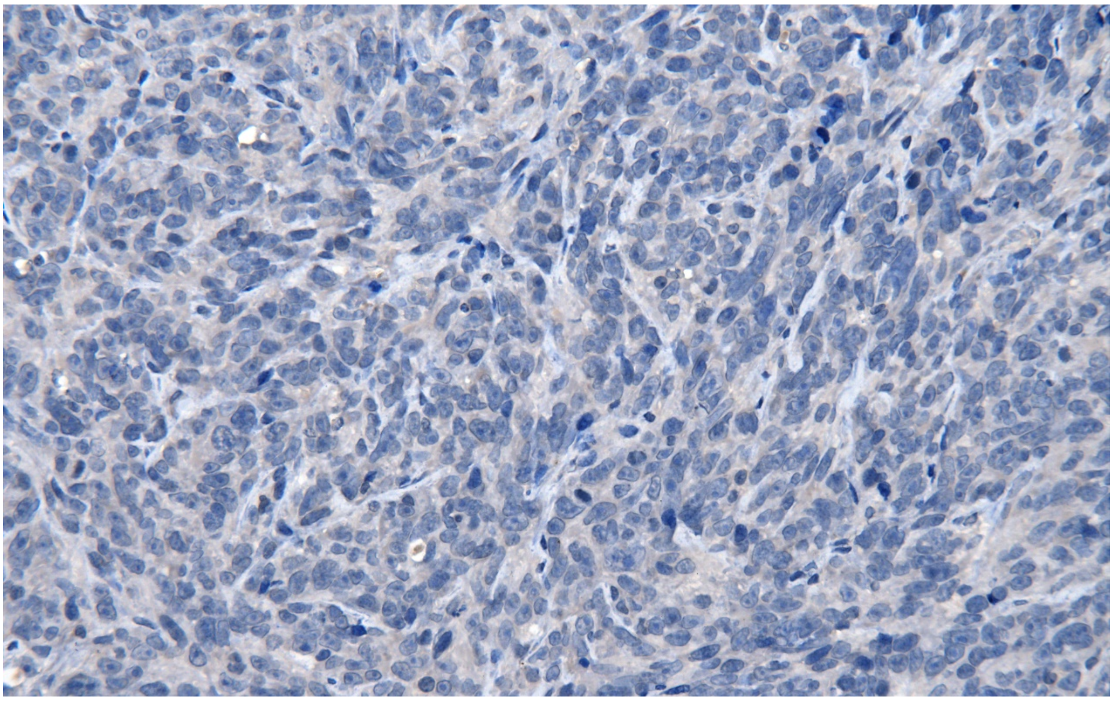

BCKDHA_MNA4_3

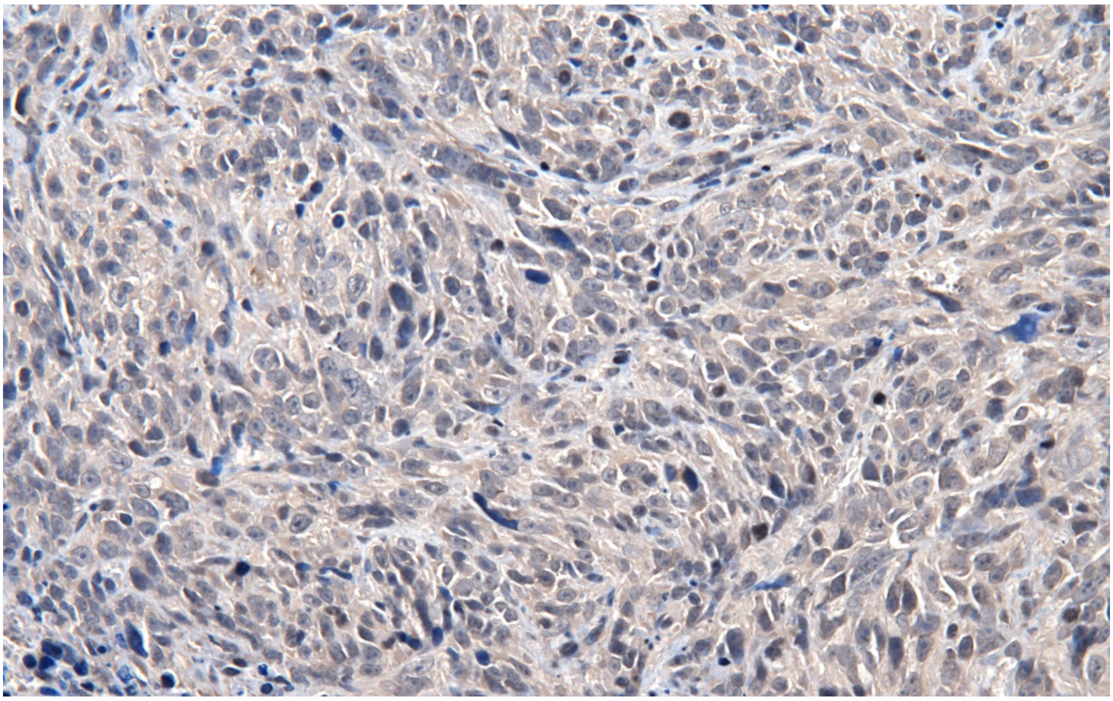

BCKDHA_MNsiA4_1

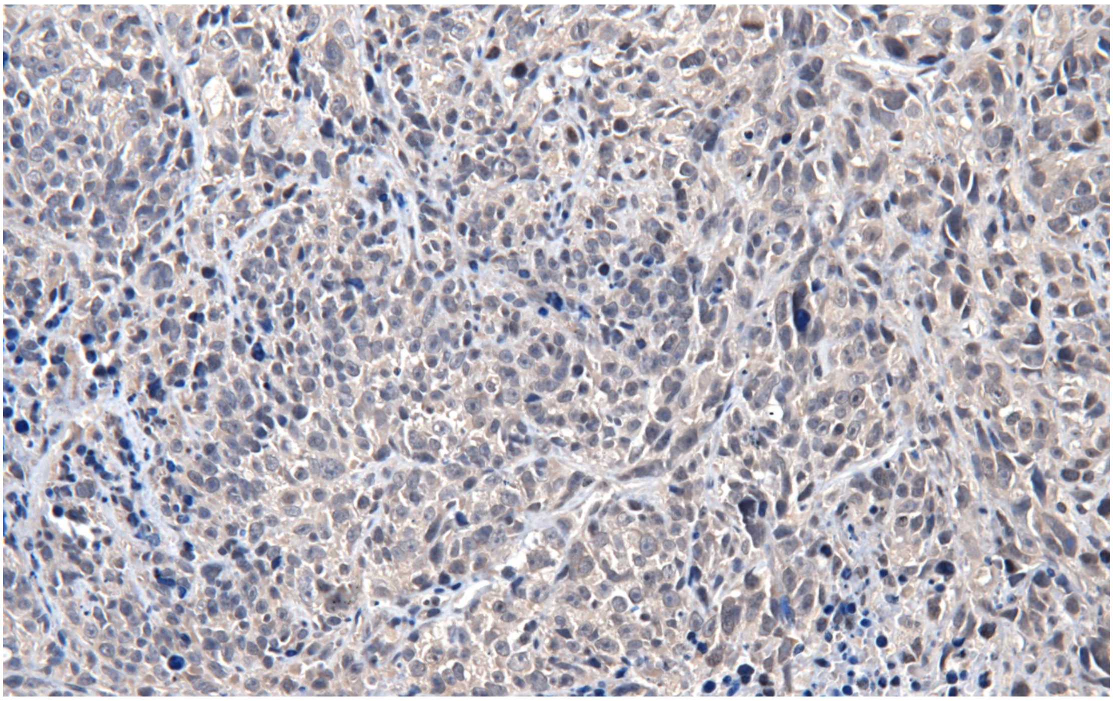

BCKDHA_MNsiA4_2

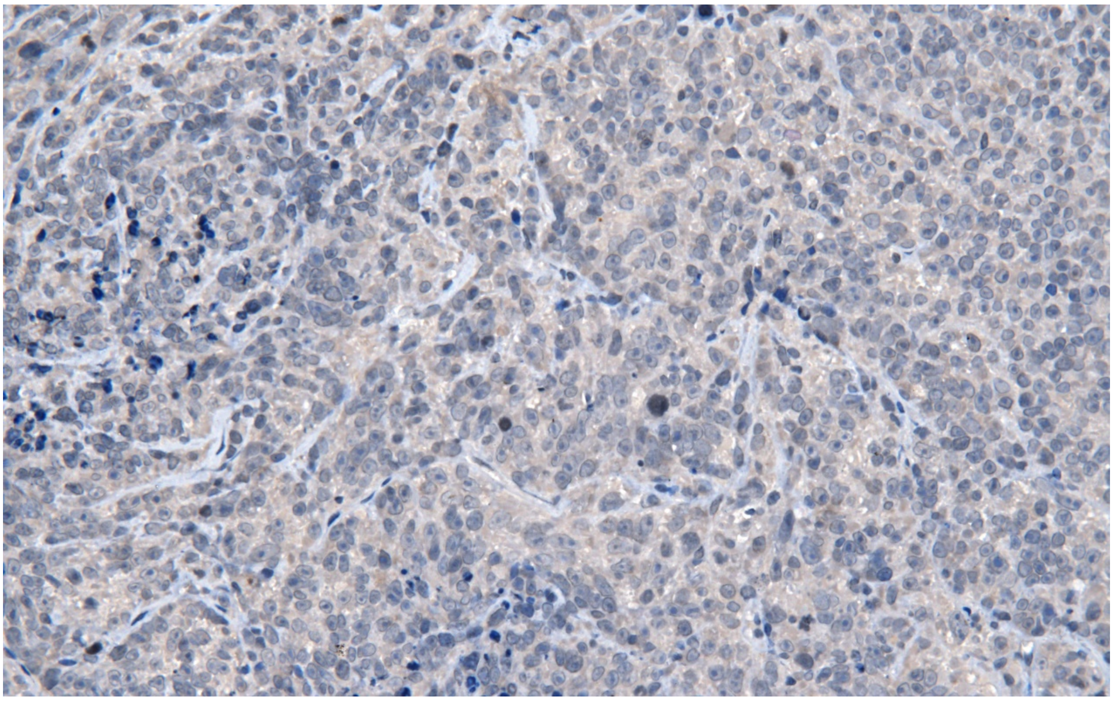

BCKDHA_MNsiA4_3

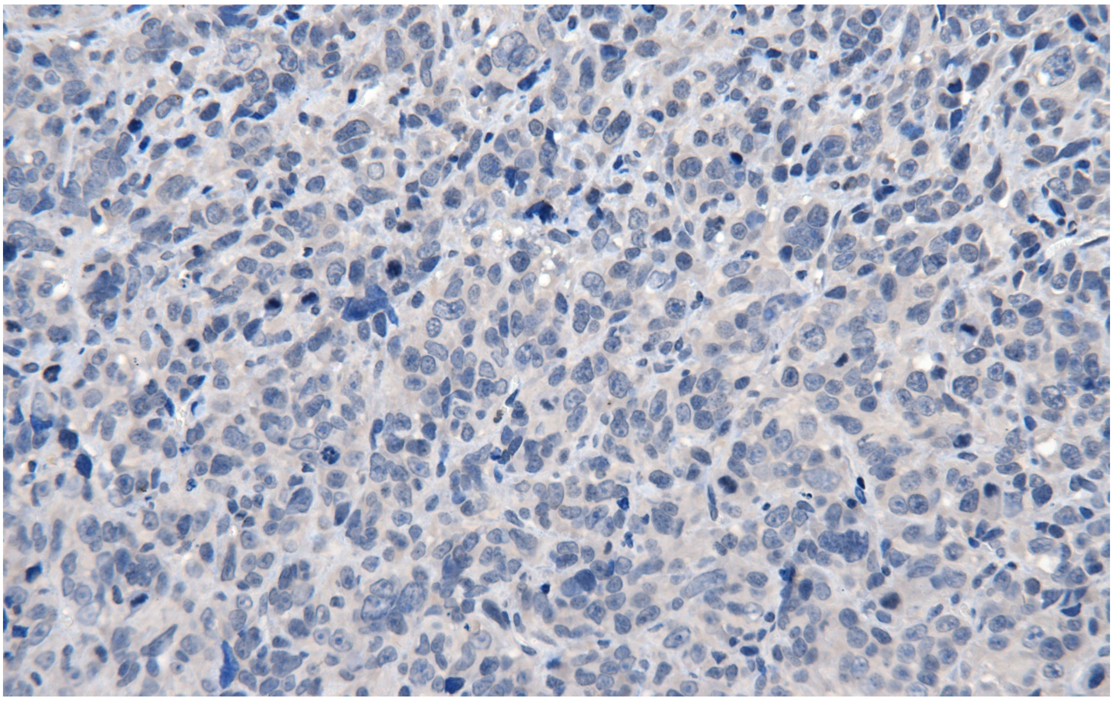

BCKDHA_siControl_1

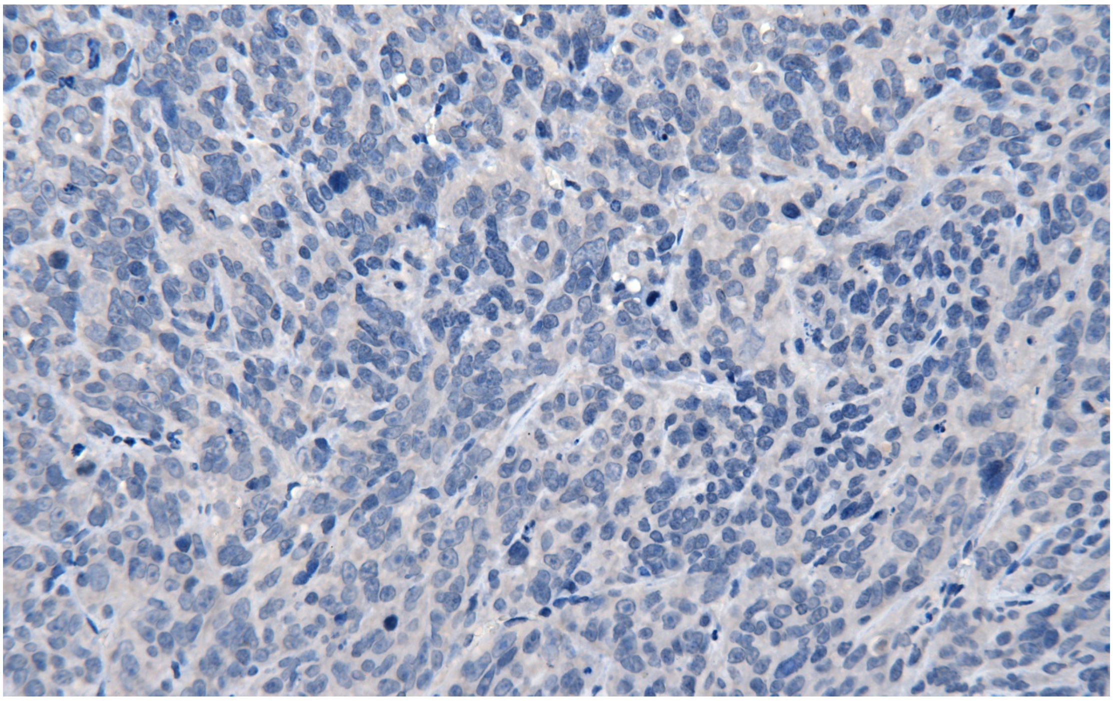

BCKDHA_siControl_2

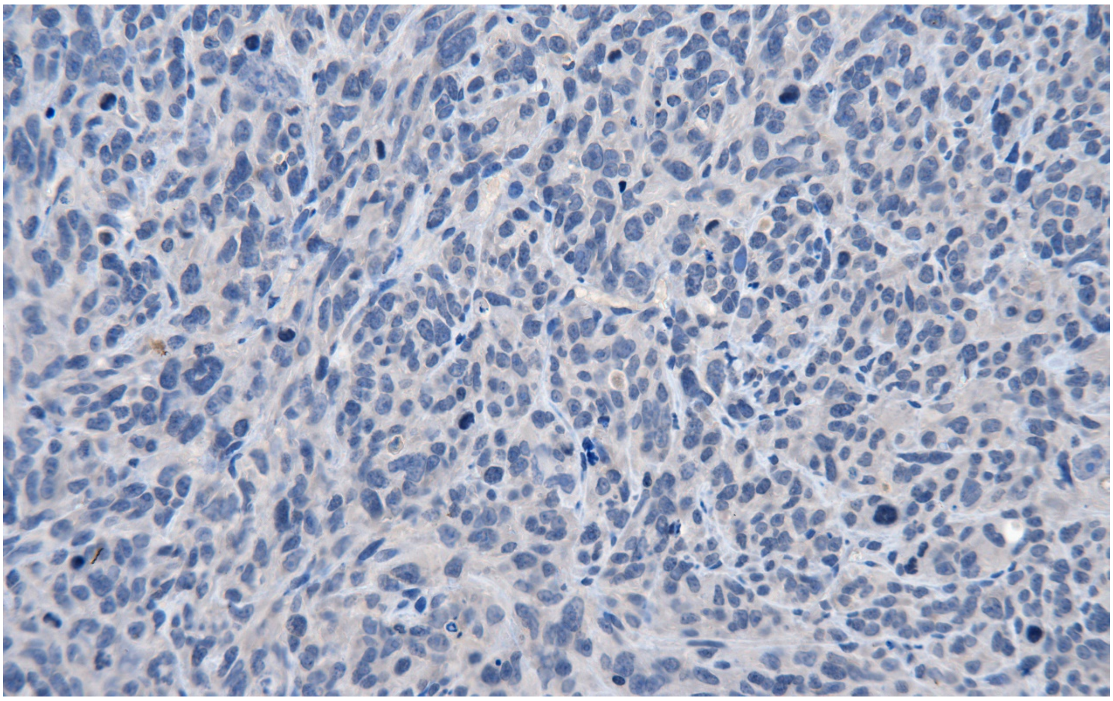

BCKDHA_siControl_3

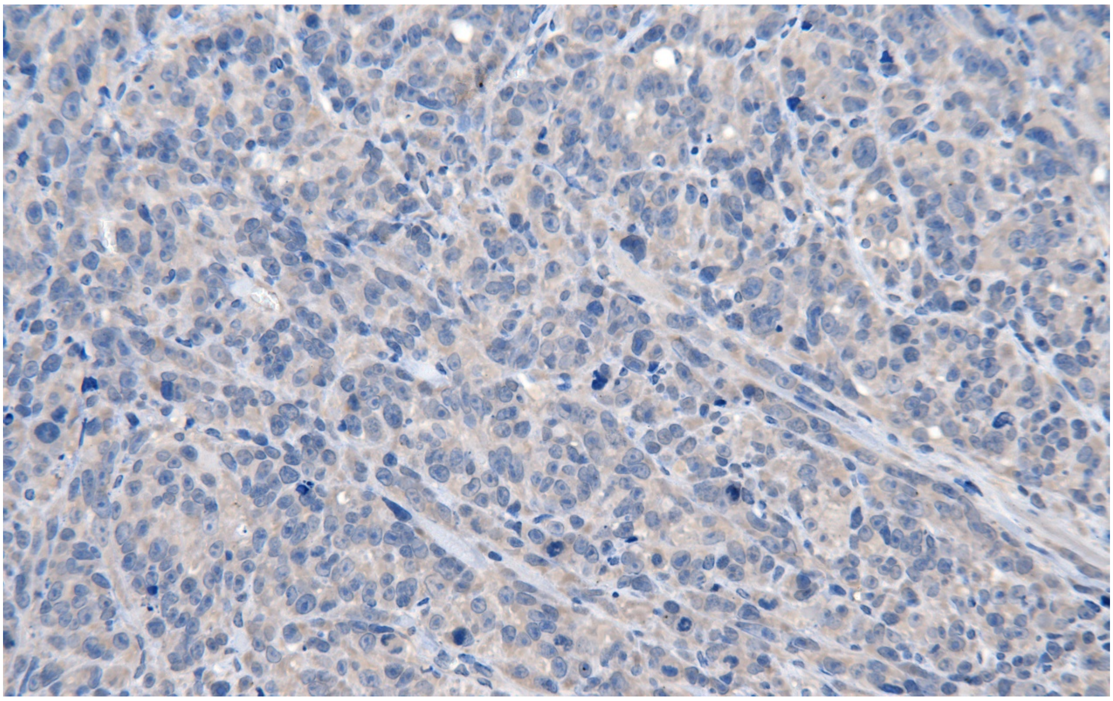

p-mTOR_A4Control_1

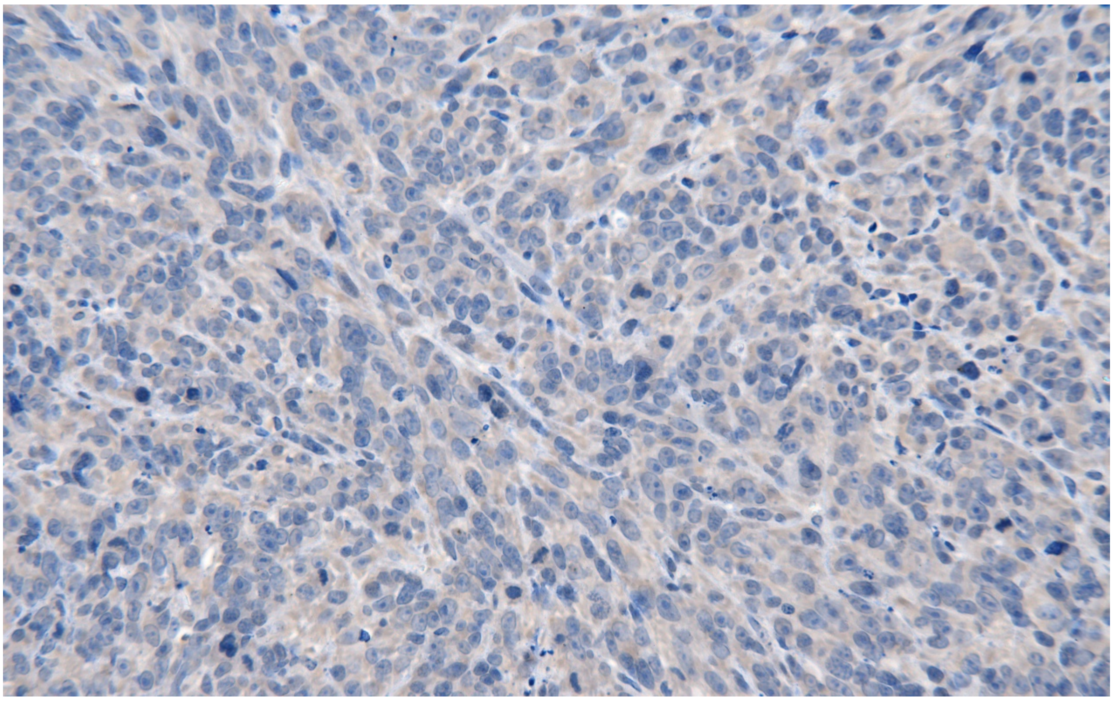

p-mTOR_A4Control_2

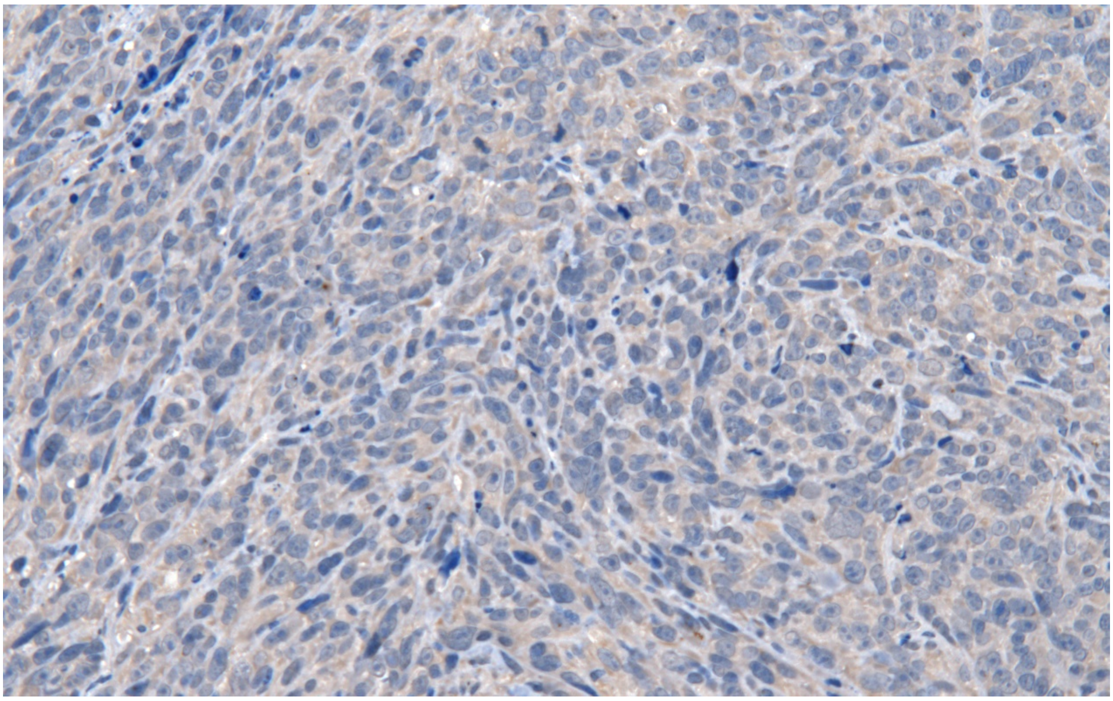

p-mTOR_A4Control_3

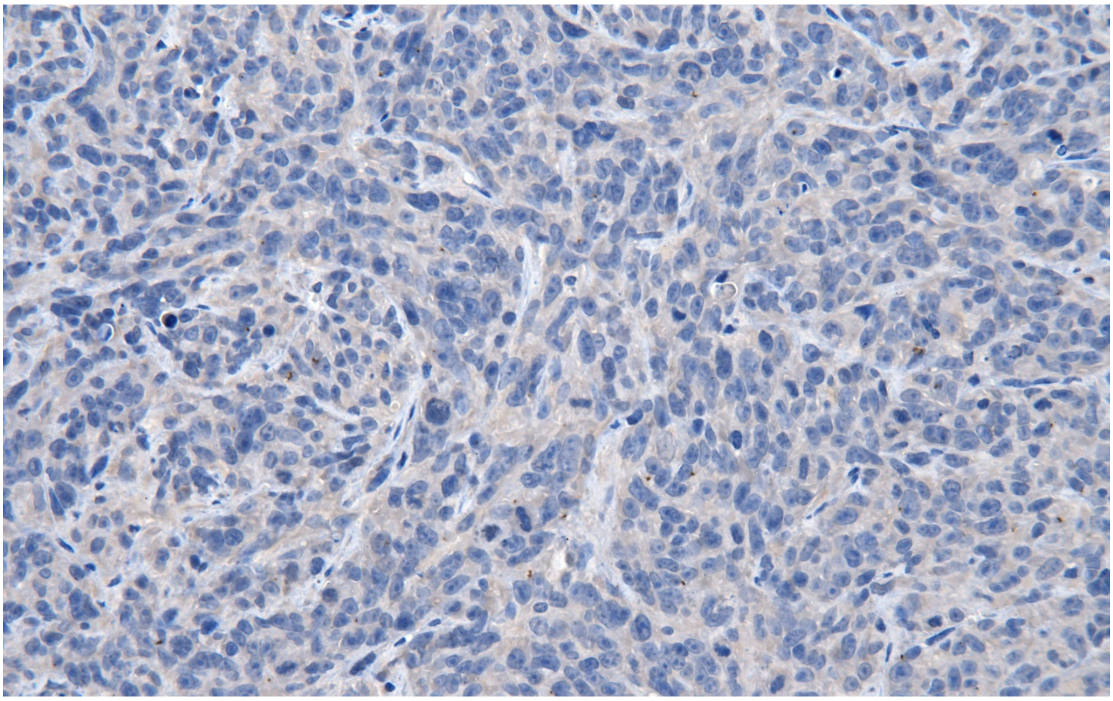

p-mTOR_MNA4_1

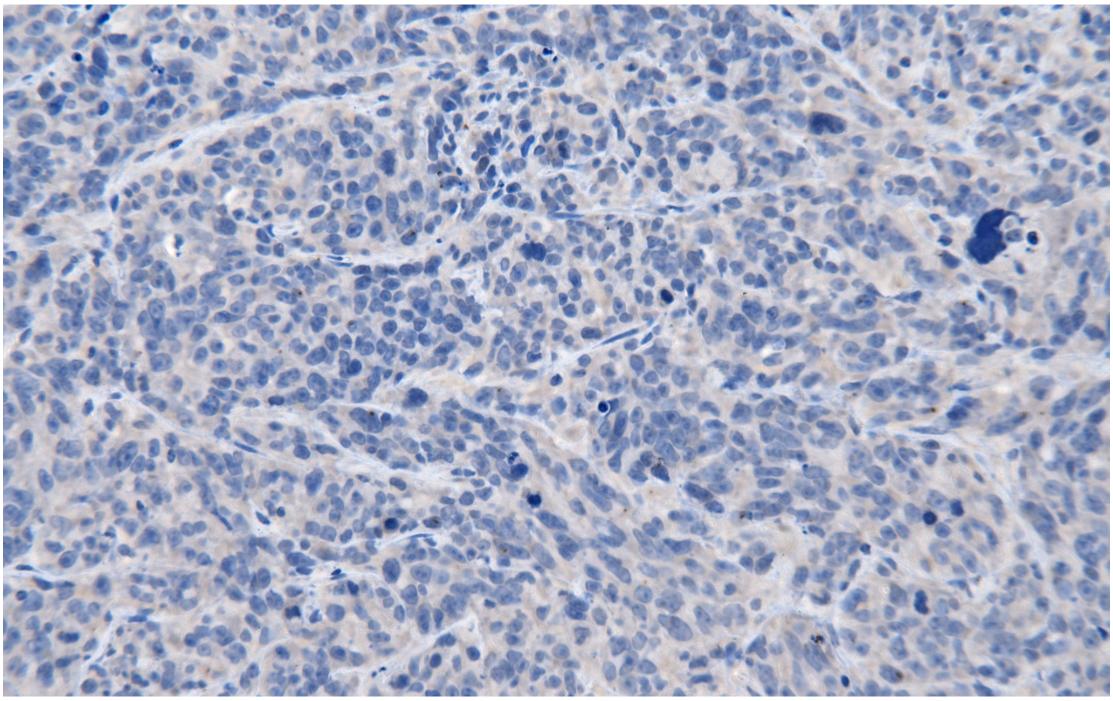

p-mTOR_MNA4_2

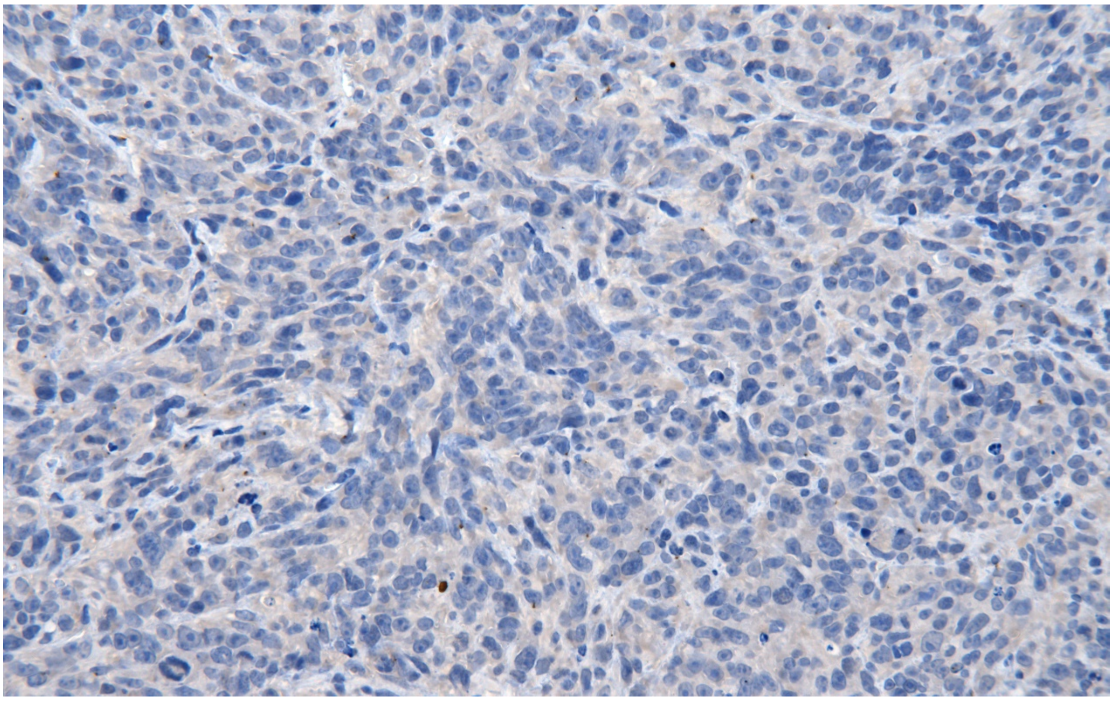

p-mTOR_MNA4_3

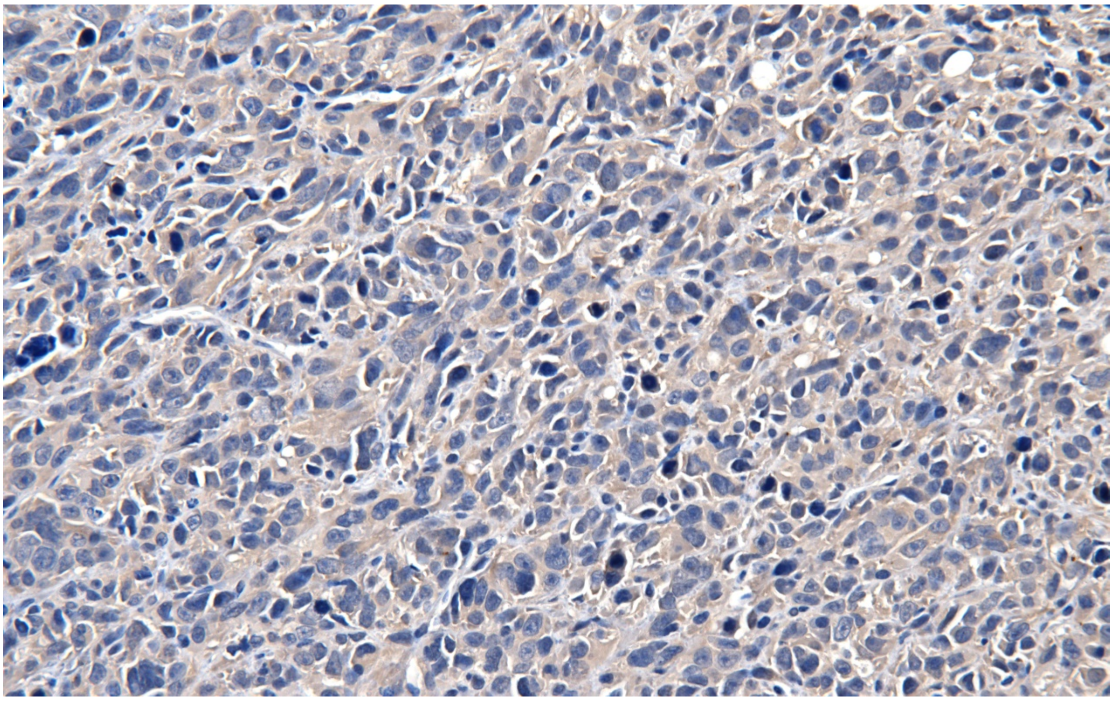

p-mTOR_MNsiA4_1

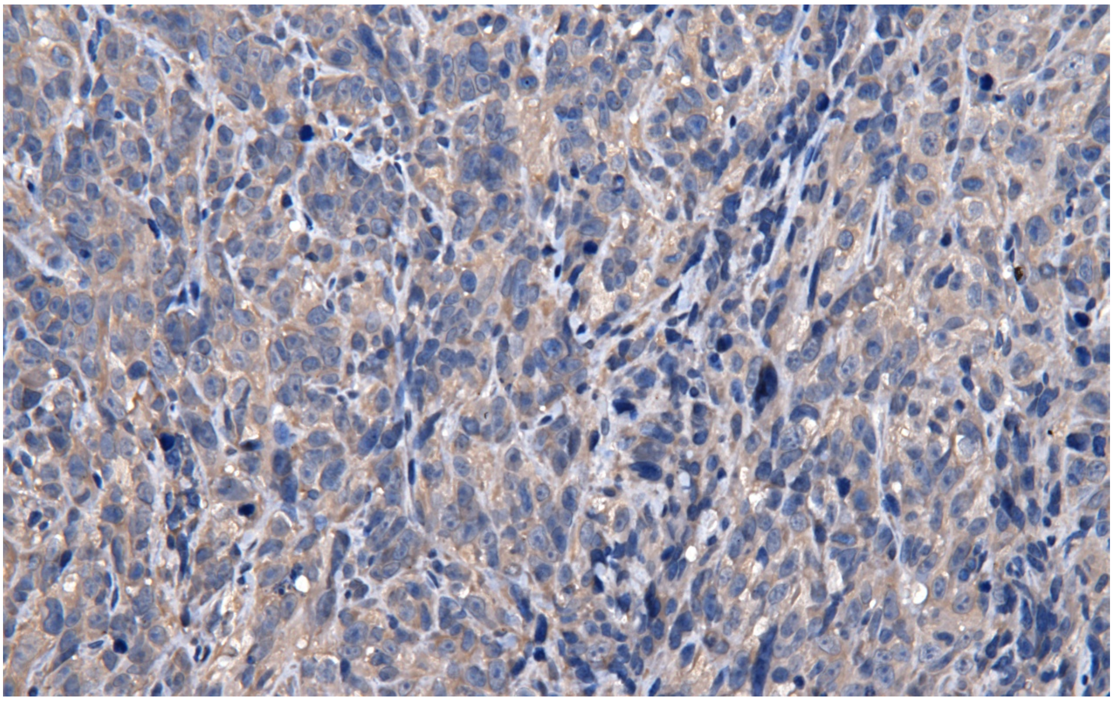

p-mTOR_MNsiA4_2

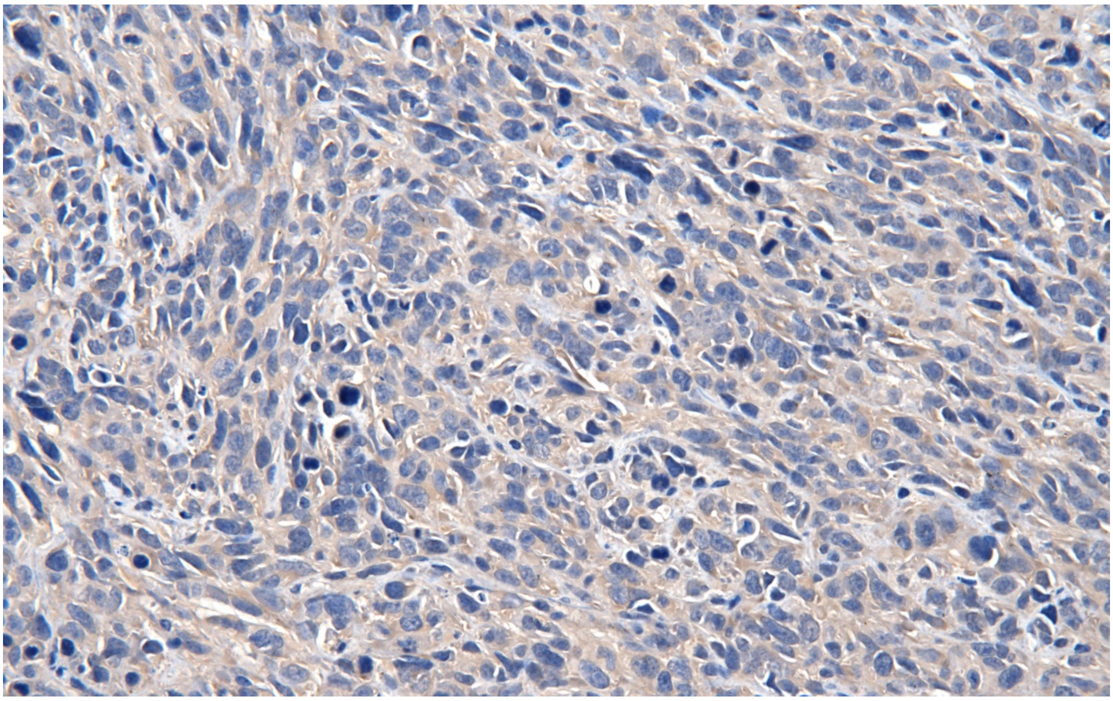

p-mTOR_MNsiA4_3

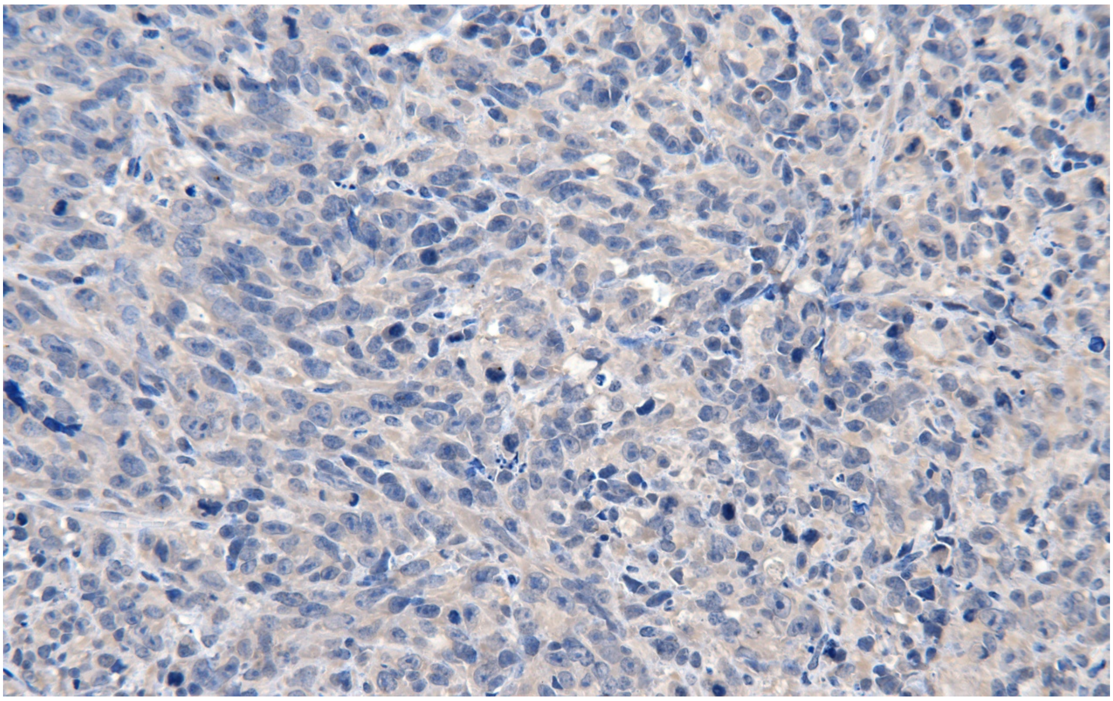

p-mTOR_siControl_1

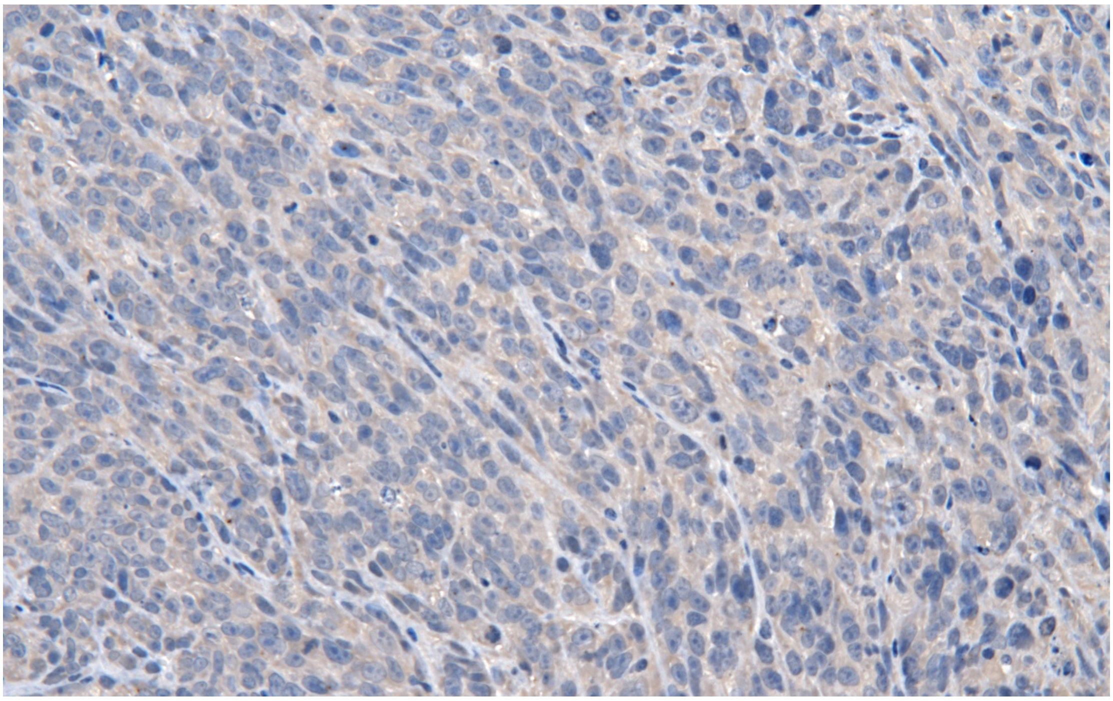

p-mTOR_siControl_2

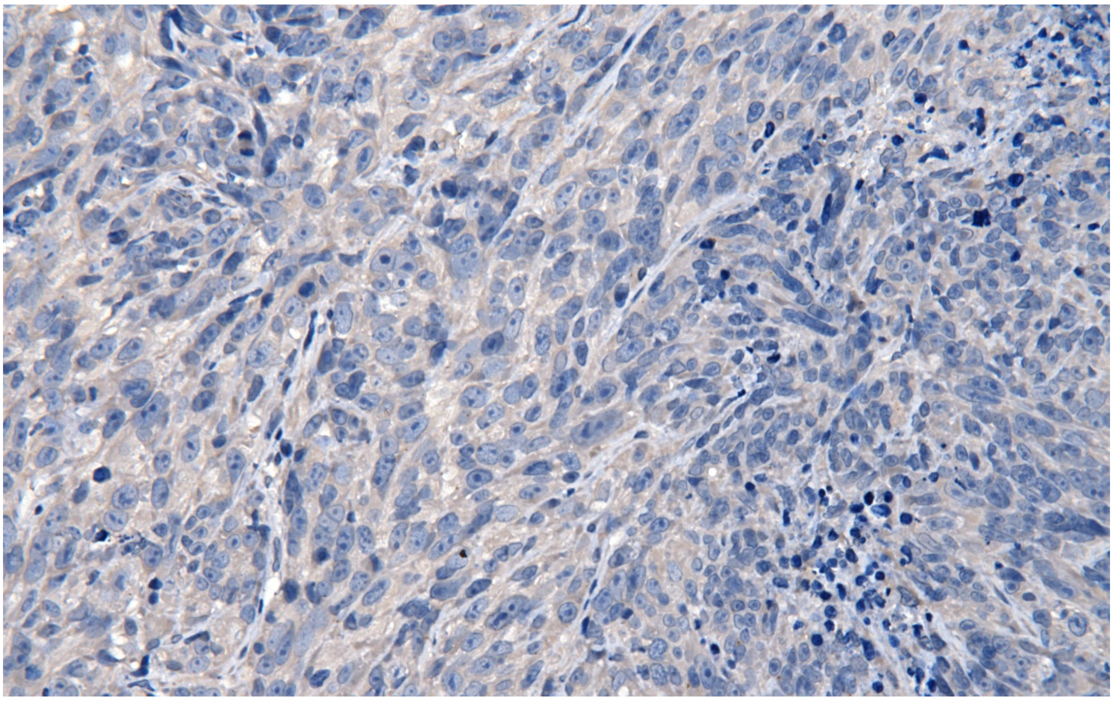

p-mTOR_siControl_3

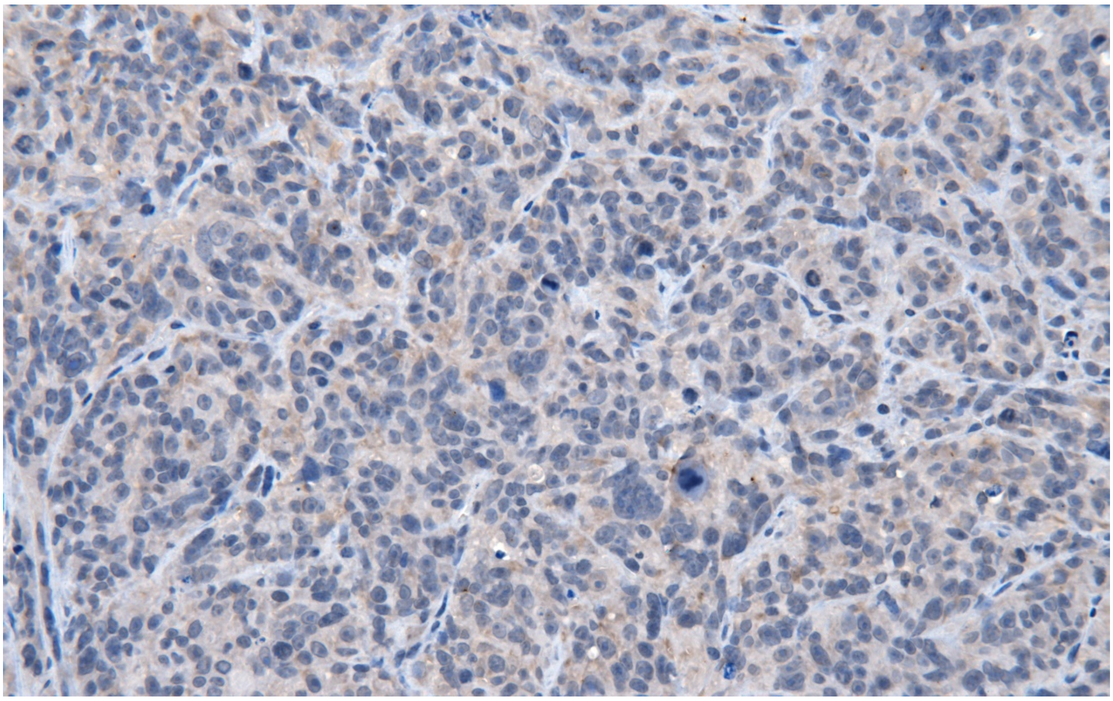

p-S6_A4Control_1

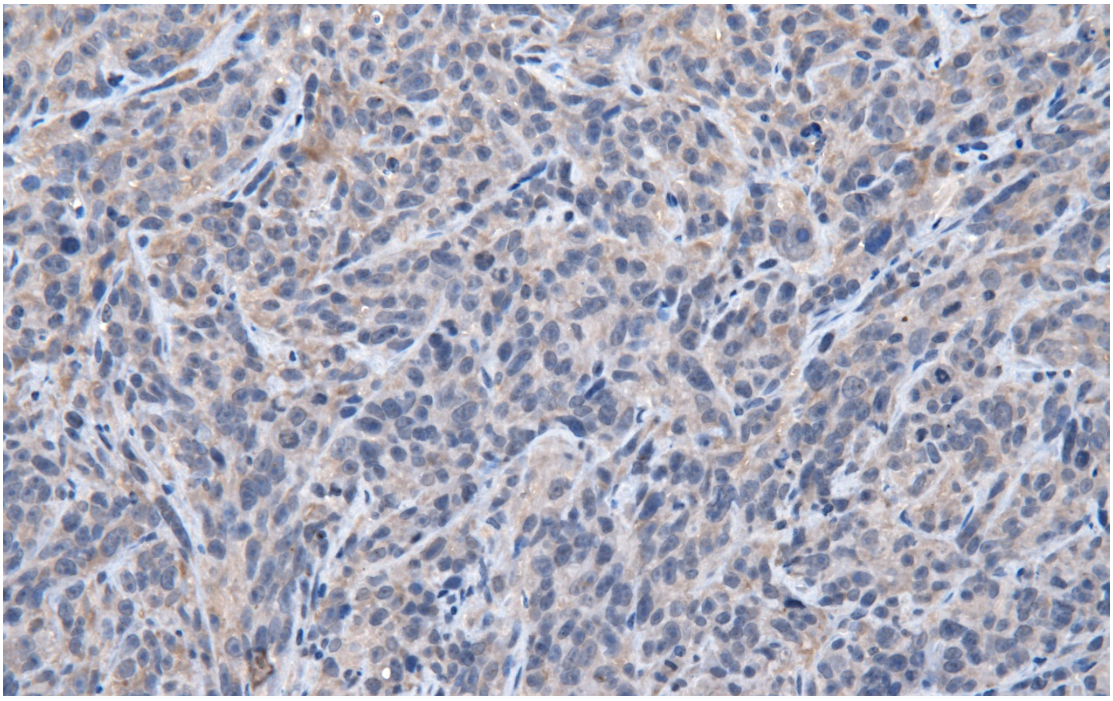

p-S6_A4Control_2

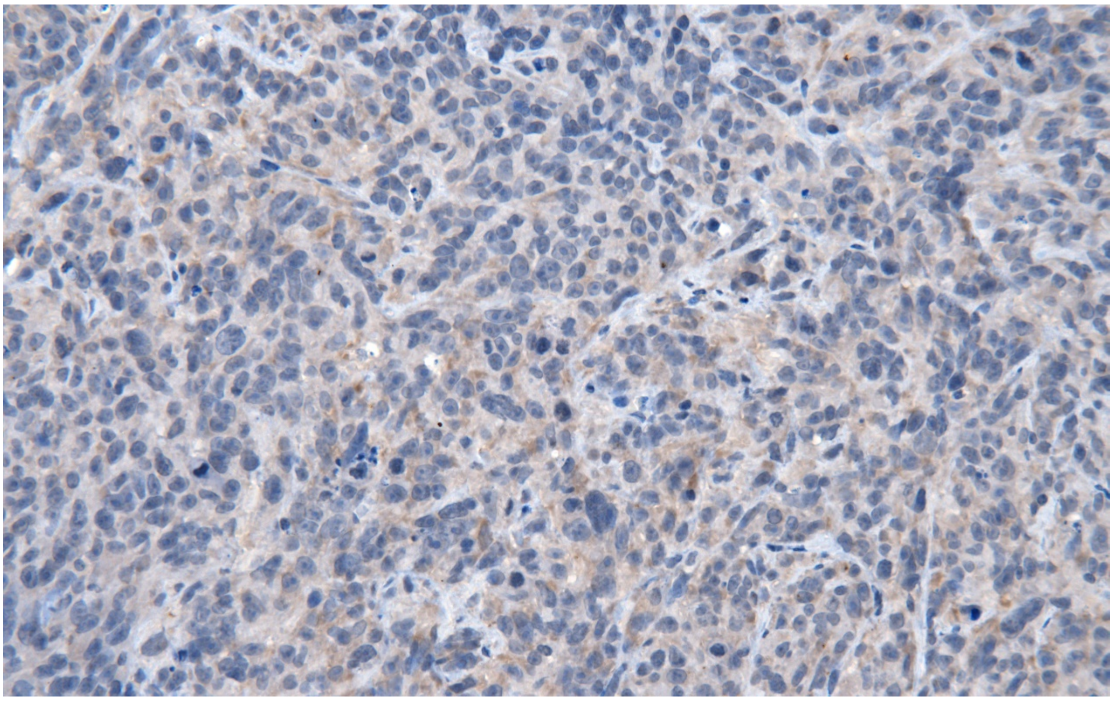

p-S6_A4Control_3

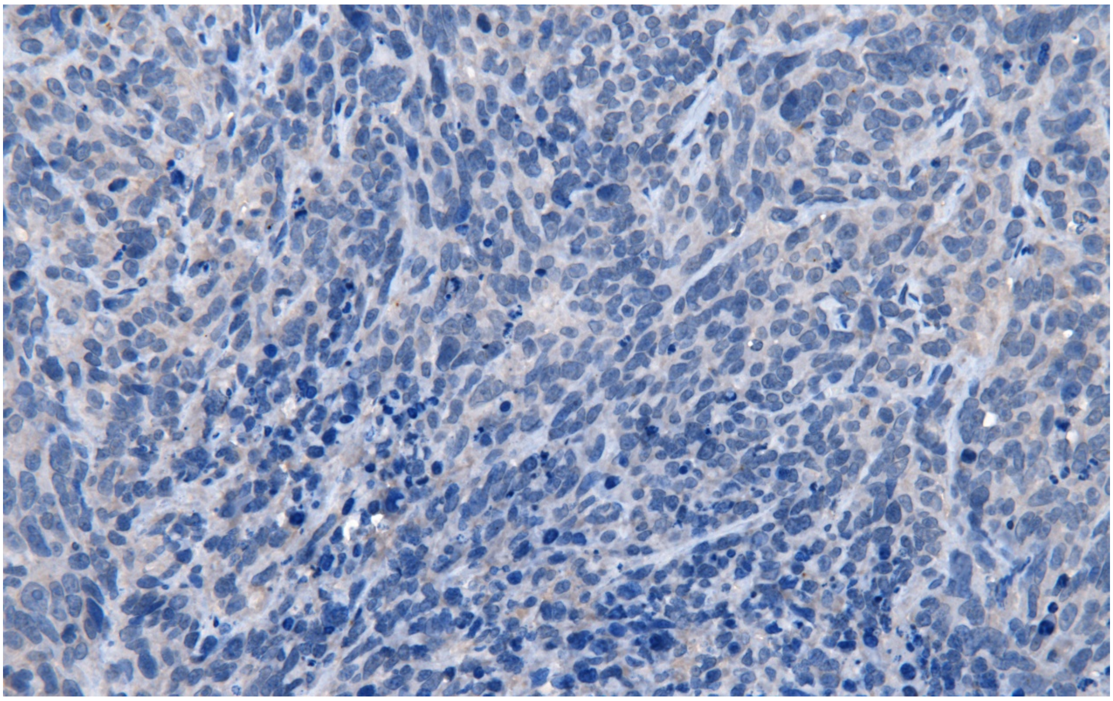

p-S6_MNA4_1

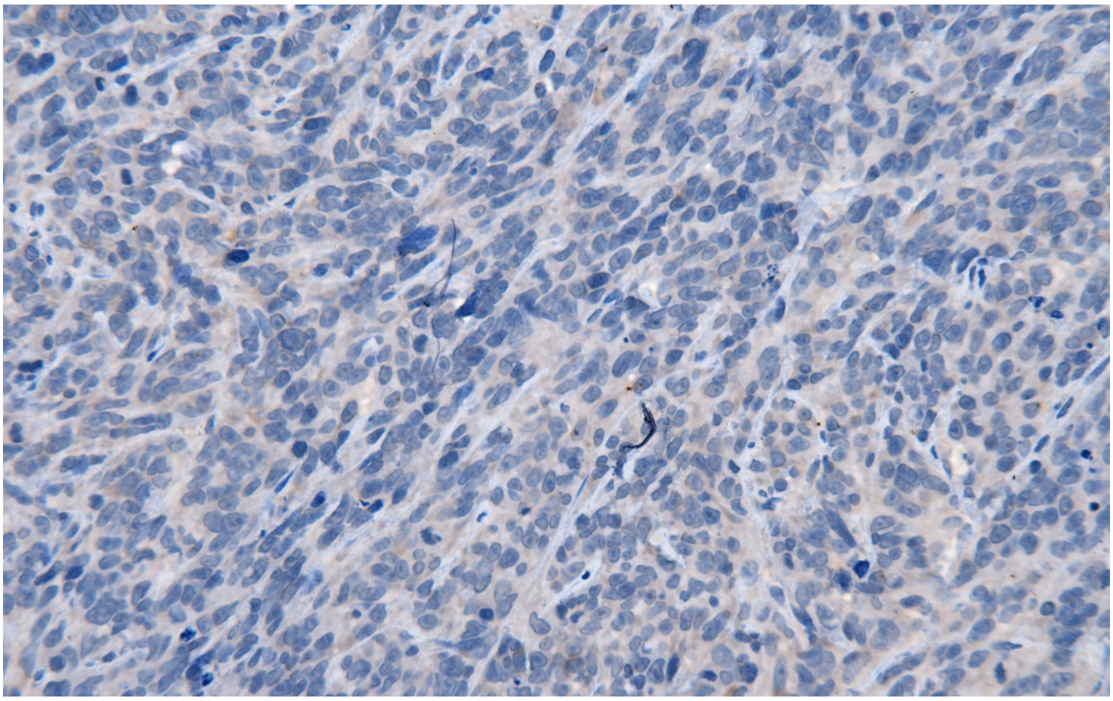

p-S6_MNA4_2

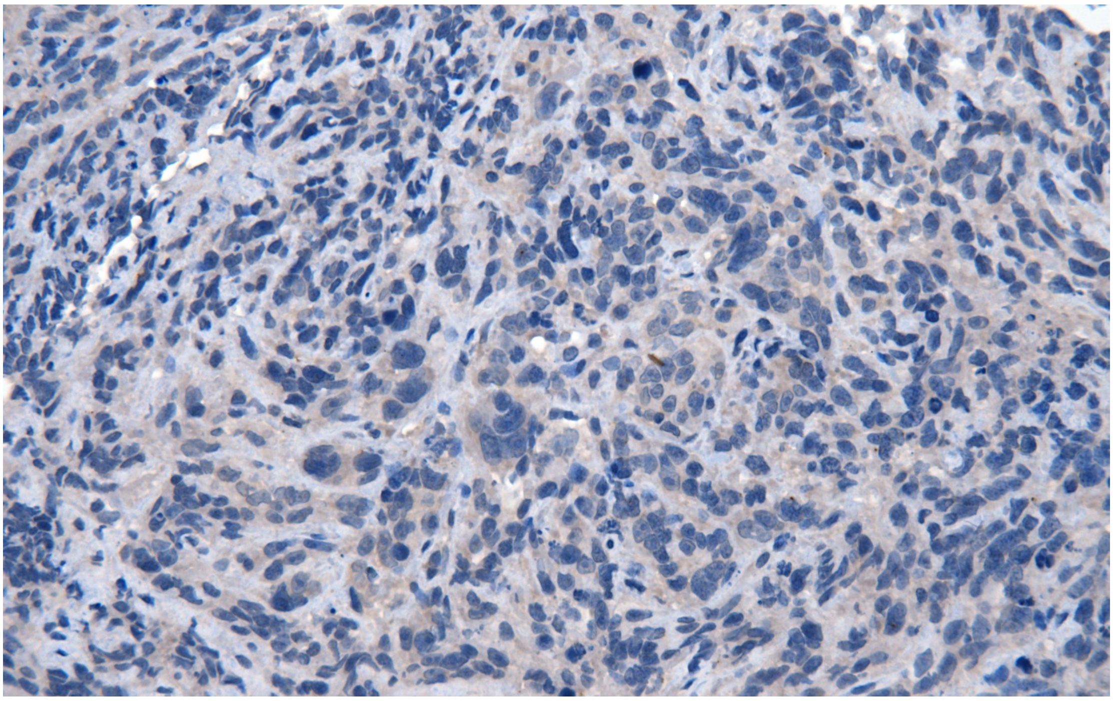

p-S6_MNA4_3

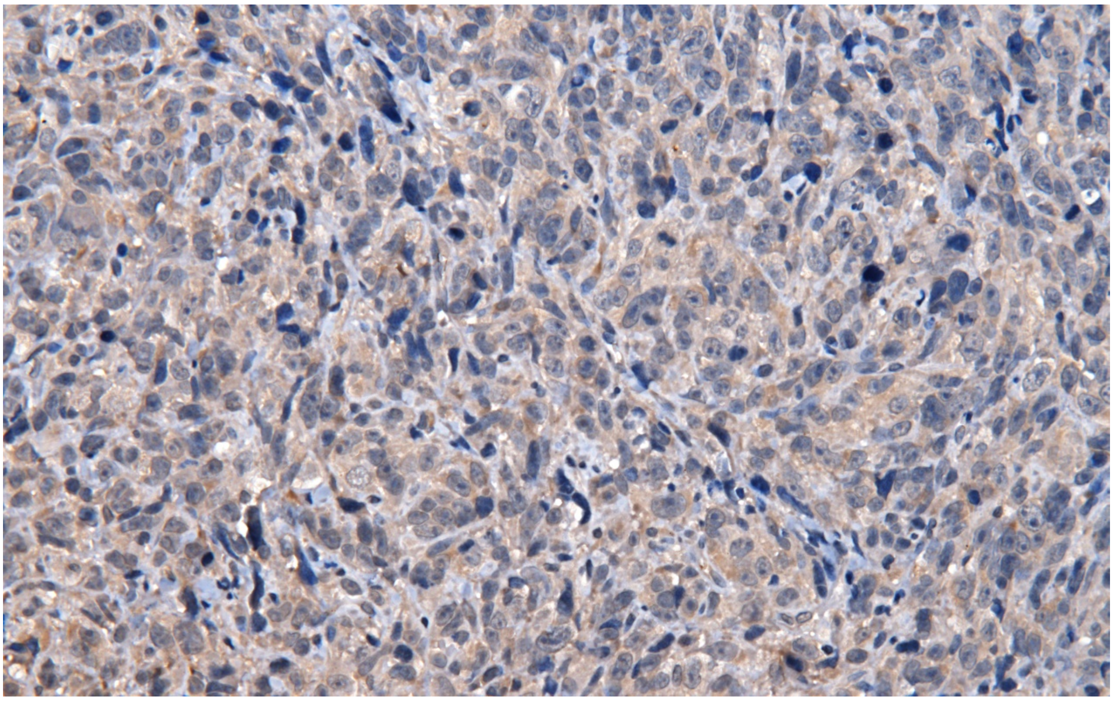

p-S6_MNsiA4_1

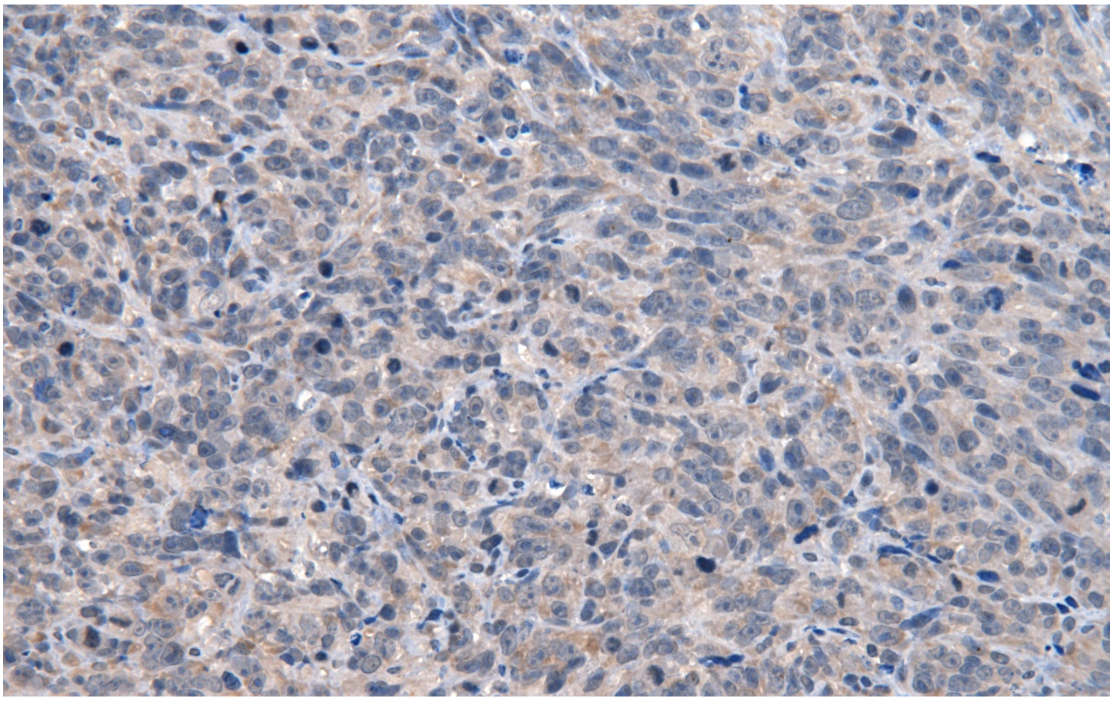

p-S6_MNsiA4_2

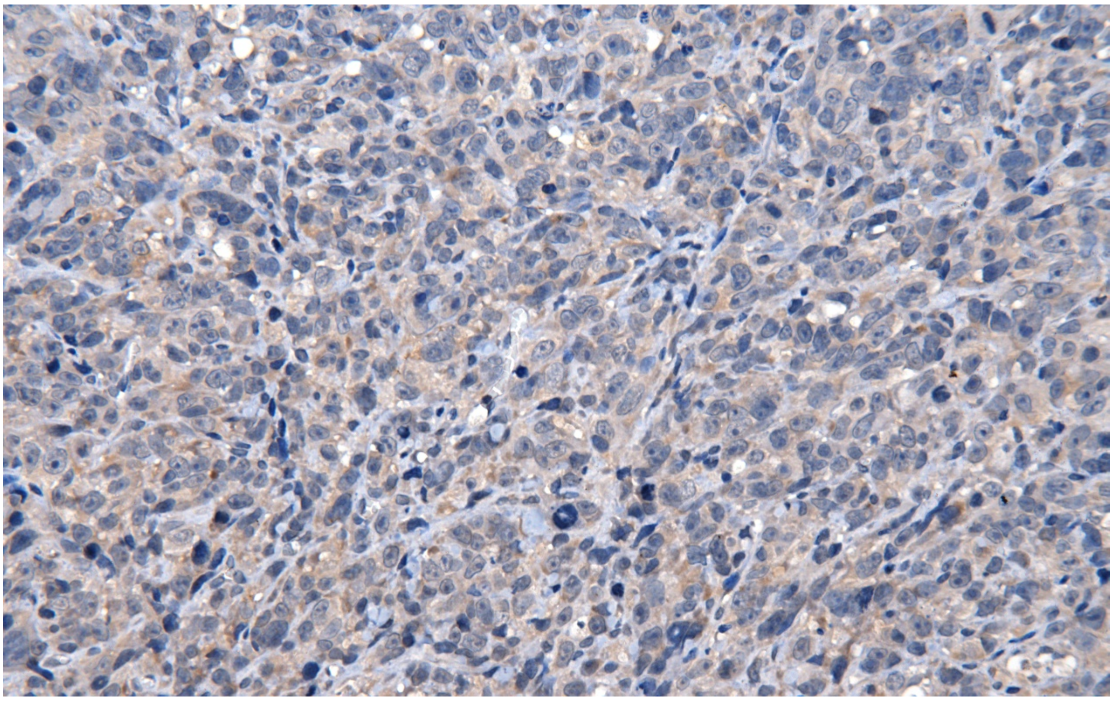

p-S6_MNsiA4_3

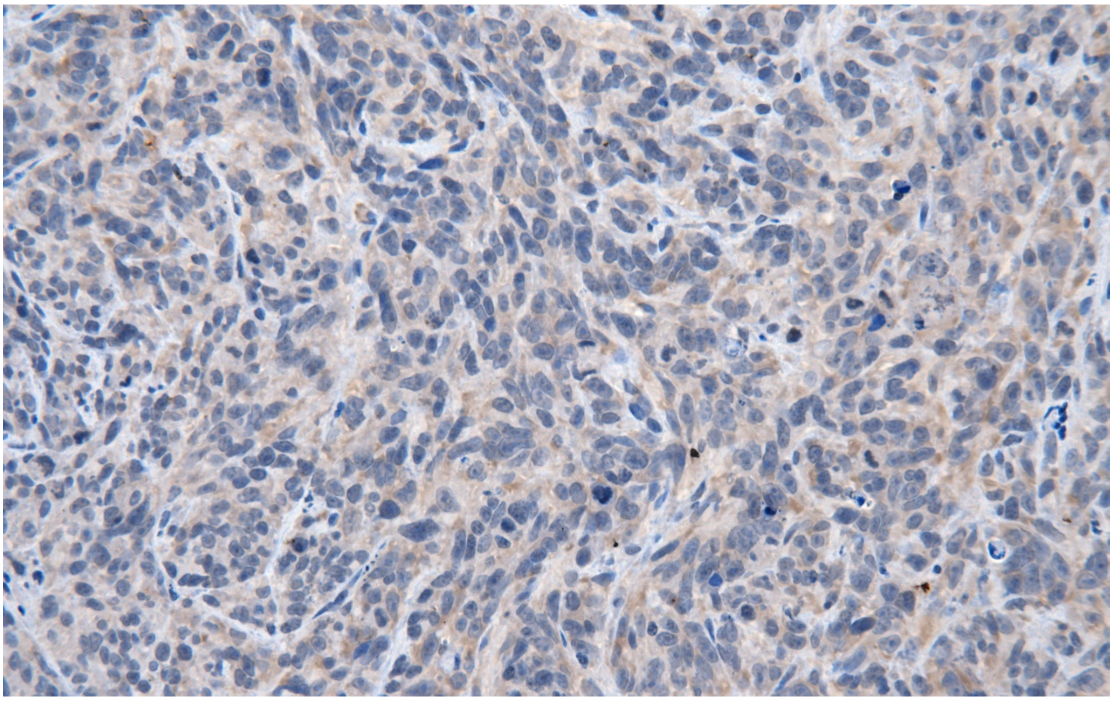

p-S6_siControl_1

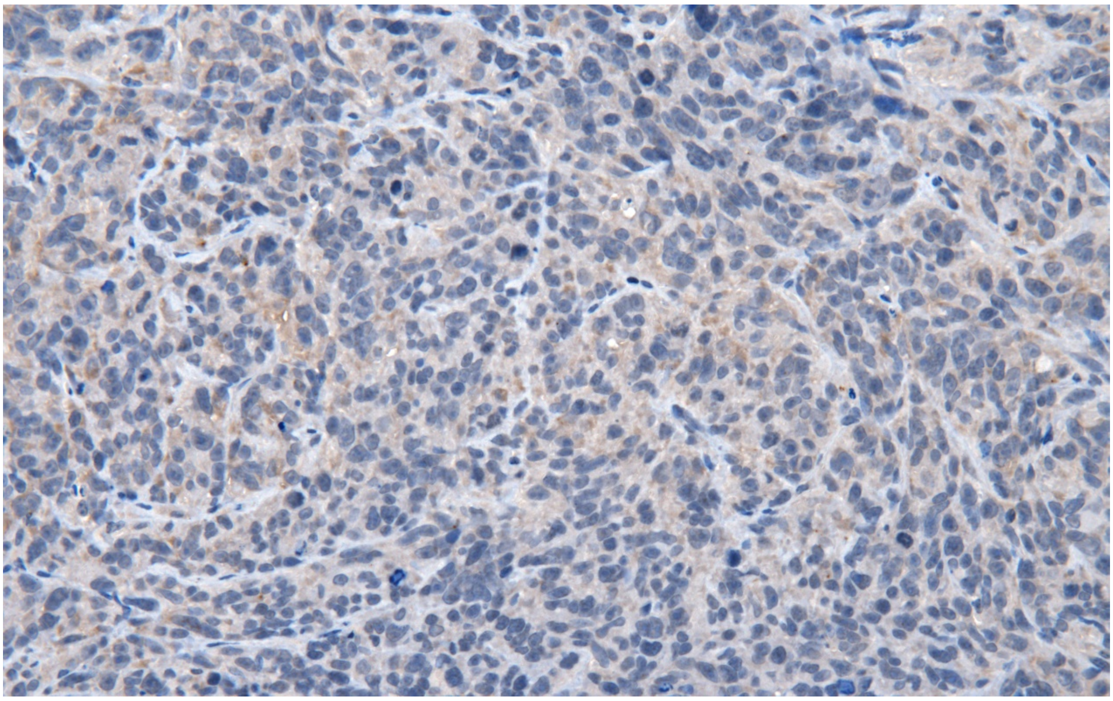
p-S6_siControl_2

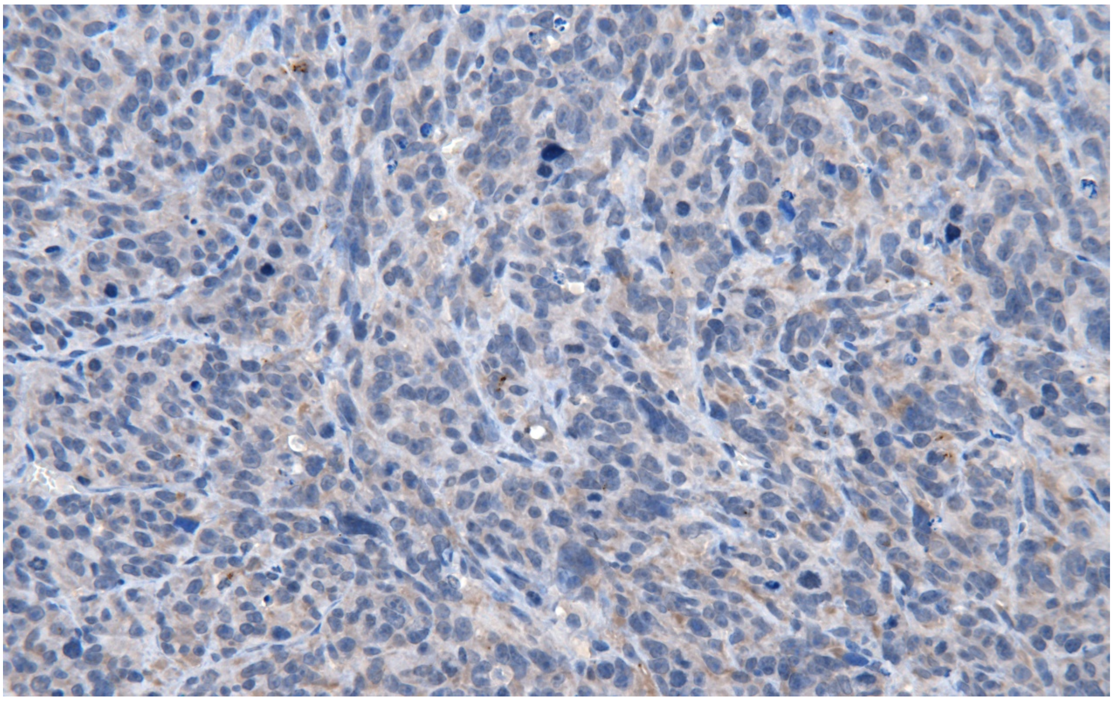
p-S6_siControl_3
